# Supplementary material for: Modulating CCTG repeat expansion toxicity in DM2 Drosophila model through TDP1 inhibition
Source: EMBO Mol Med. 2025 Mar 25;17(5):967–92. doi: 10.1038/s44321-025-00217-3 (PMC12081759; doi:10.1038/s44321-025-00217-3)
Supplement: Supplementary file 4 — Appendix [file 44321_2025_217_MOESM4_ESM.pdf]

Table of Contents

1. Appendix Figure S1: CCTG repeat expansion did not affect *gkt* expression………………… 2
2. Appendix Figure S2: *TDP1/gkt* knockdown restored the abnormal flight ability of DM2 flies……………………………………………………………………………………… 3
3. Appendix Figure S3: *TDP1/gkt* knockdown corrected the abnormal localization of MBNL1/mbl……………………………………………………………………………... 5
4. Appendix Figure S4: *TDP1*/*gkt* loss-of-function rescued alternative splicing in DM2………. 6
5. Appendix Figure S5: Validation of the CCTG Repeat Amplification PCR Protocol…………. 7
6. Appendix Figure S6: *TDP1/gkt* overexpression did not affect CCTG repeat size……………. 8
7. Appendix Figure S7: *TDP1/gkt* knockdown reduced CUG toxic RNA aggregation…………. 9

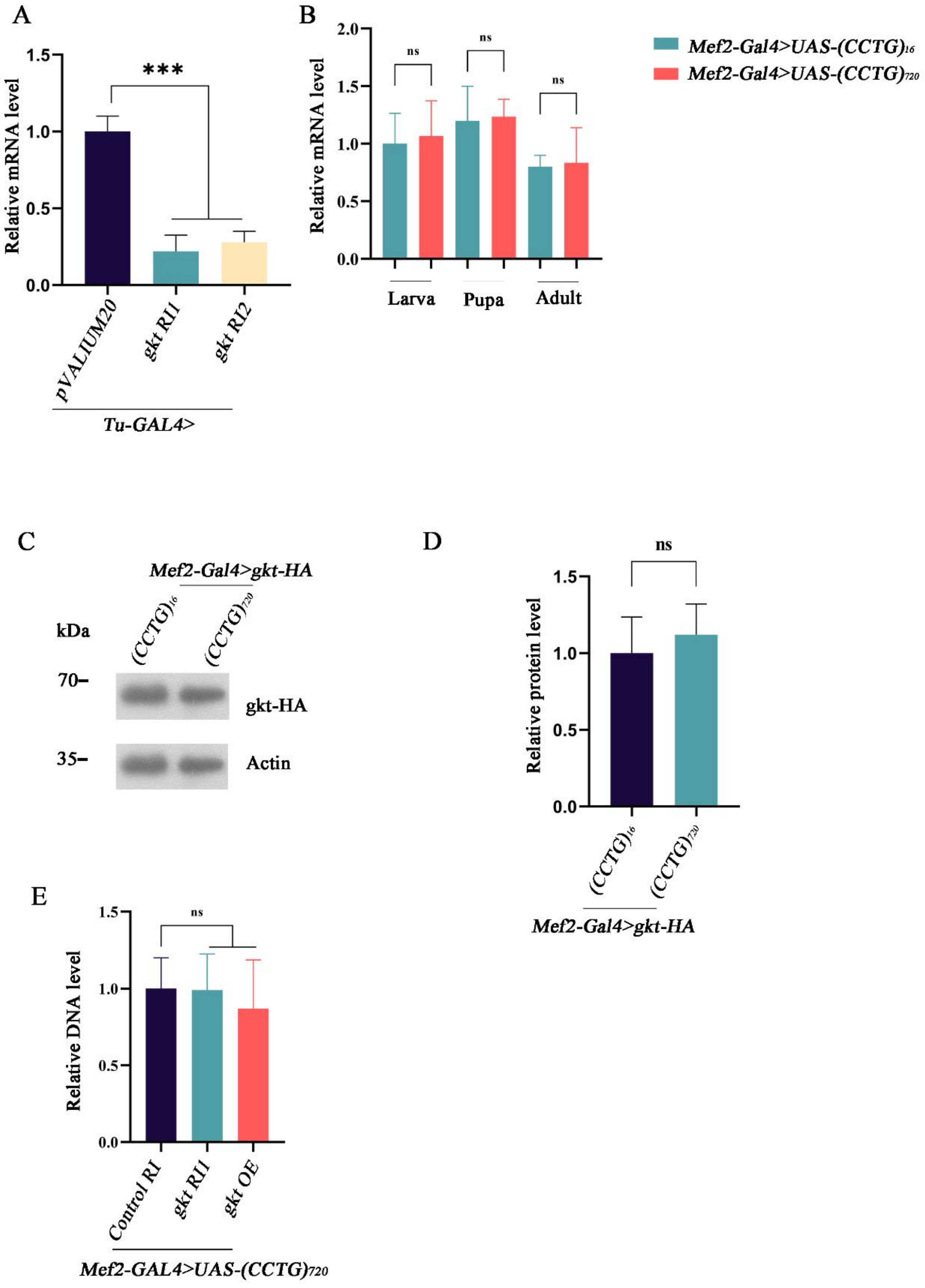

Appendix S1. CCTG repeat expansion did not affect the expression of *gkt*. (A) Relative mRNA level of *gkt* across different genotypes at 7-days-old from five biological replicates. Data are mean ± SD. Two-tailed, unpaired t-test. ****P* = 0.0007 *pWALIUM20* versus *gkt RI 1*, ****P* = 0.0005 *pWALIUM20* versus *gkt RI 2*. (B) Relative mRNA level of *gkt* across *Mef2-Gal4>UAS-(CCTG)*$_{16}$ and *Mef2-Gal4>UAS-(CCTG)*$_{720}$ at 3rd larva, pupa and Adult from five biological replicates. Data are mean ± SD. Two-tailed, unpaired t-test. (C) Immunoblot against HA across

Mef2-Gal4>UAS-(CCTG)16+UAS-gkt-HA and *Mef2-Gal4>UAS-(CCTG)720+UAS-gkt-HA* at 7-days-old. (D) Quantification of (A) from five biological replicates. Data are mean ± SD. Two-tailed, unpaired t-test. (E) Relative DNA level of CCTG transgene across different genotypes at 15-days-old from five biological replicates. Data are mean ± SD. Two-tailed, unpaired t-test.

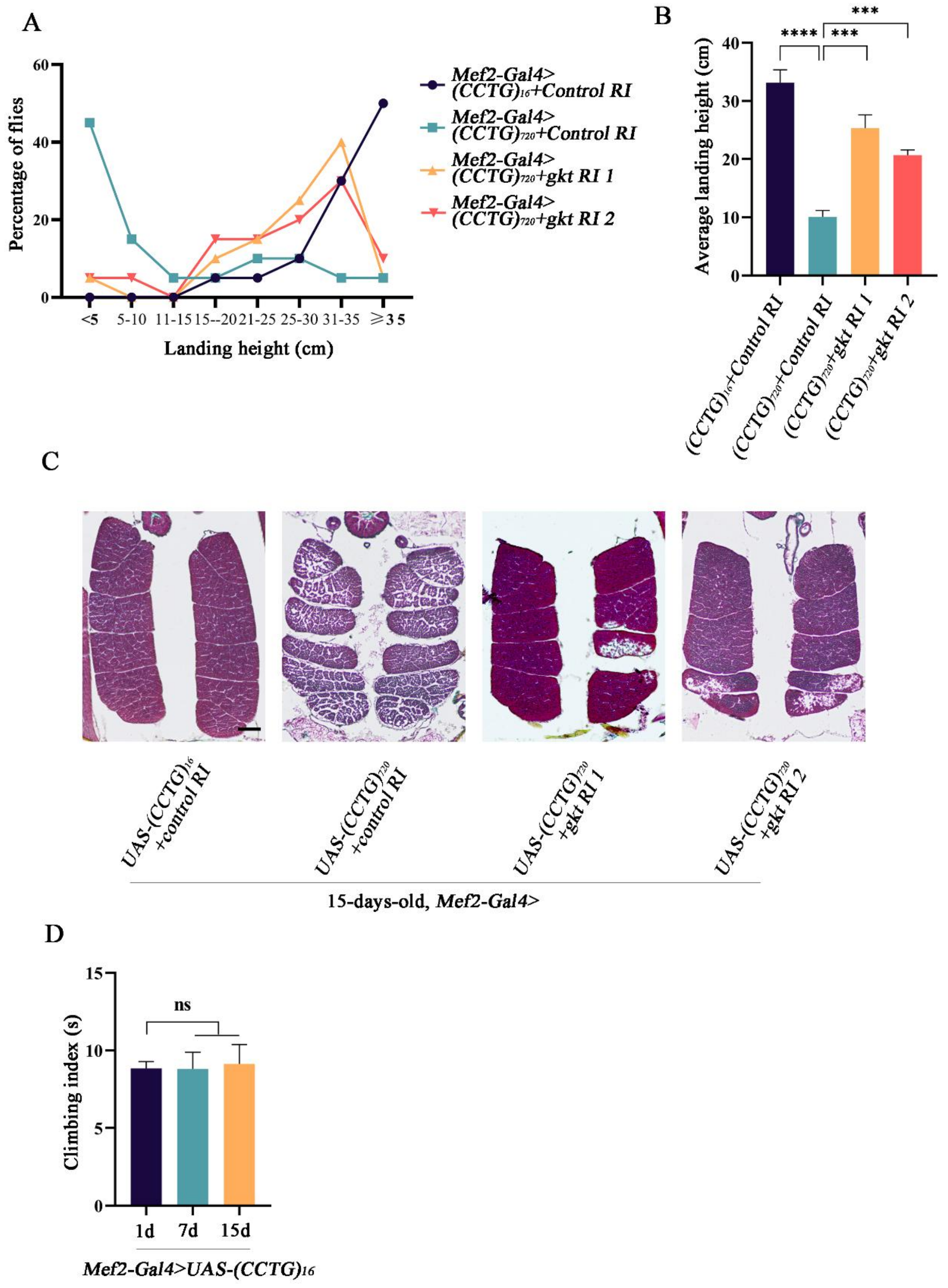

Appendix Figure S2. *TDP1/gkt* knockdown restored the abnormal flight ability of DM2 flies. (A)

The landing height across different genotypes at 15-days-old. Twenty animals per sample. (B) Quantification of mean landing height from five biological replicates. Data are mean ± SD. Two-tailed, unpaired t-test. ****P < 0.0001 $(CCTG)_{16}$ versus $(CCTG)_{720}$, ***P = 0.0005 $(CCTG)_{720}$+Control RI versus $(CCTG)_{720}$ + gkt RI 1, ***P = 0.0002 $(CCTG)_{720}$+Control RI versus $(CCTG)_{720}$ + gkt RI 1. (C) Representative masson staining images of paraffin-embedded adult thoraces showing IFMs of flies at 15-days-old. Scale bars 100 μm. (D) Quantification of climbing index of *Mef2-Gal4>(CCTG)*$_{720}$ at 1-day-old, 7-days-old and 15-days-old from five biological replicates. Data are mean ± SD. Two-tailed, unpaired t-test.

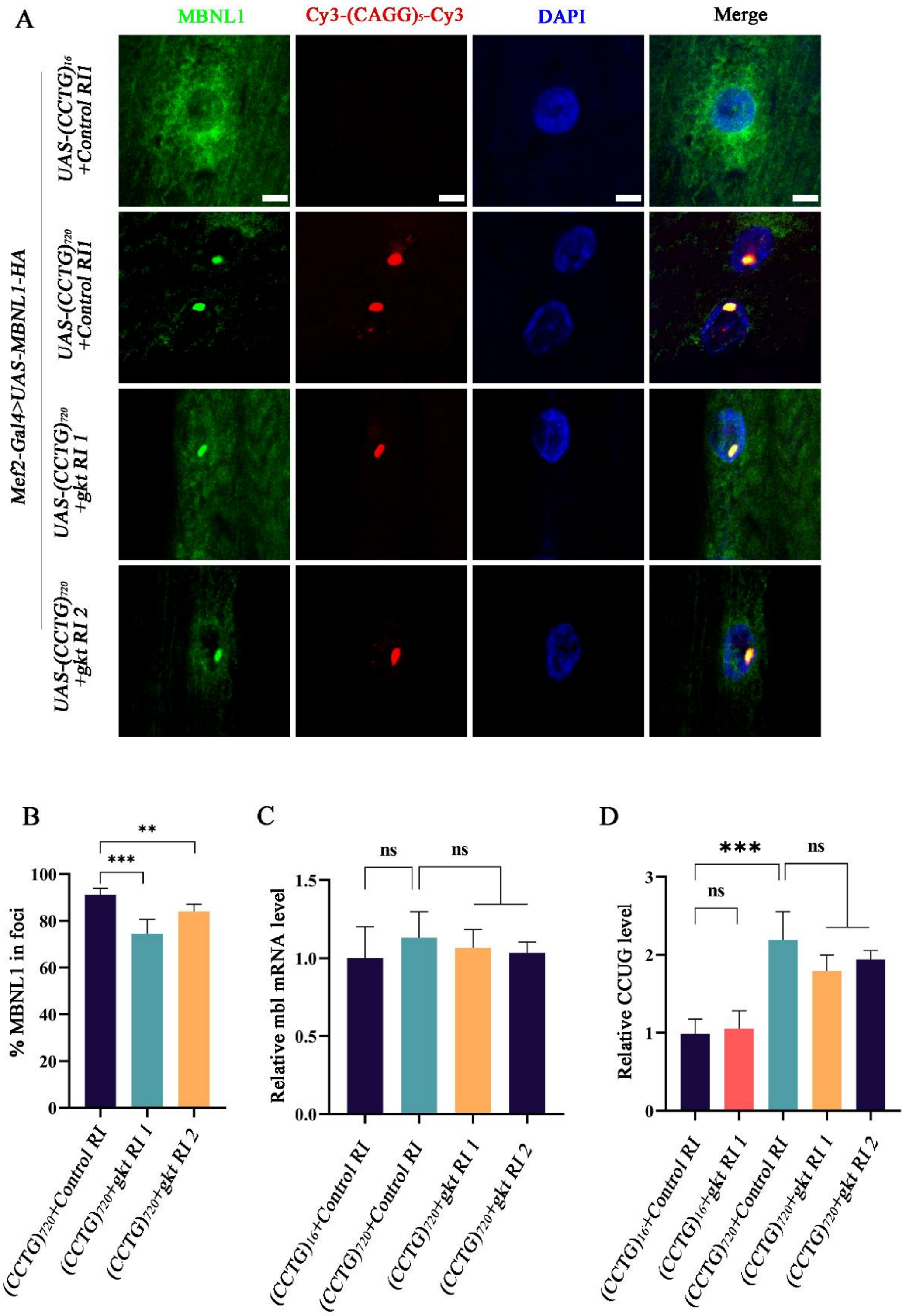

Appendix Figure S3. *TDP1/gkt* knockdown corrected the abnormal localization of MBNL1/mbl. (A) Representative image of colocalized nuclear foci FISH and MBNL1 immunofluorescence. Scale bars 5 µm. (B) Quantification at least ten animals of each genotype. Data are mean ± SD. Two-tailed, unpaired t-test. ****P* = 0.0005, ***P* = 0.005. (C) Relative mRNA level of *mbl* across different genotypes at 15-days-old. Data are mean ± SD. Two-tailed, unpaired t-test. (D) Relative mRNA level of *CCUG* across different genotypes at 15-days-old. Data are mean ± SD. Two-tailed,

unpaired t-test. ***$P = 0.0002$.

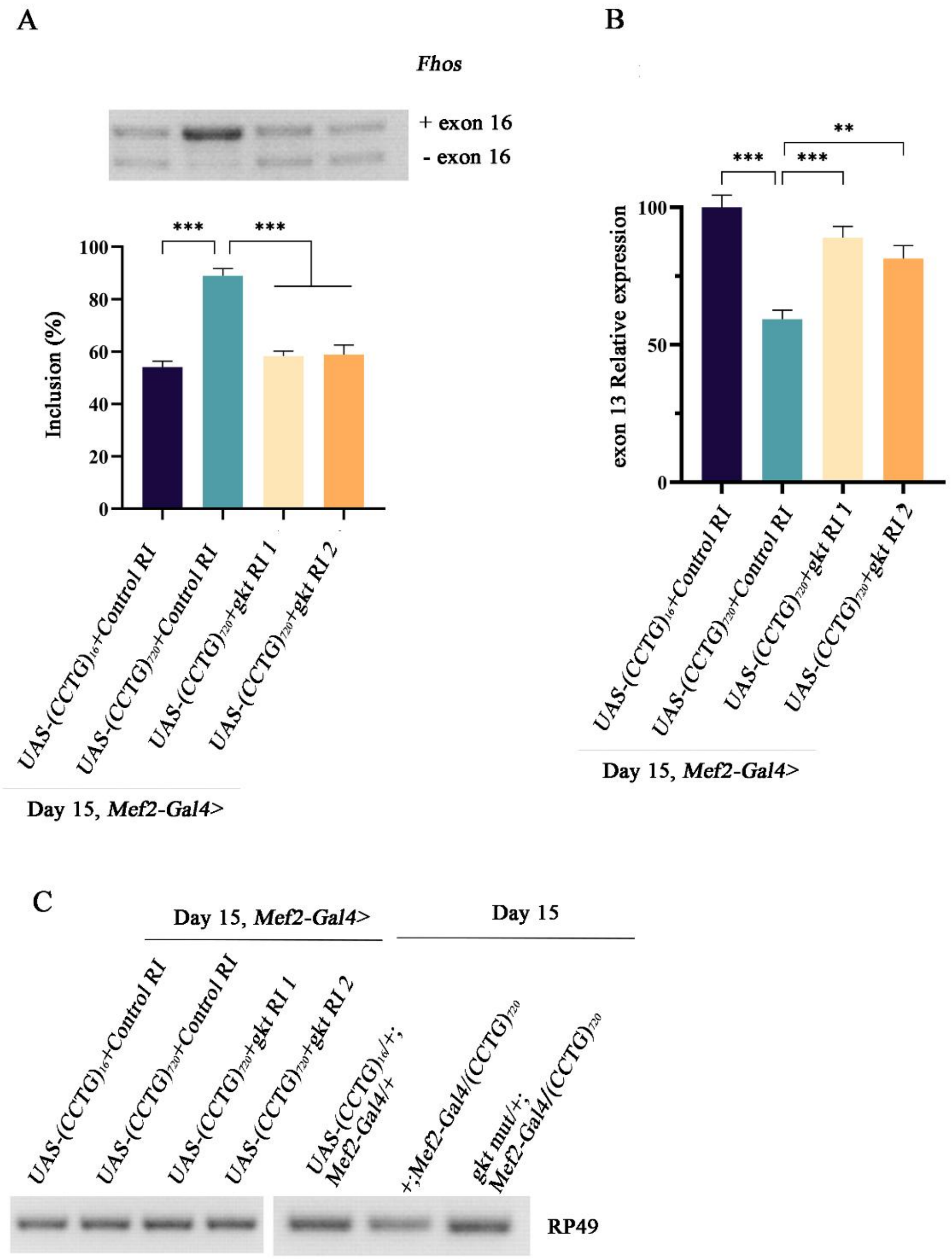

Appendix Figure S4. TDP1/*gkt* loss-of-function rescued alternative splicing in DM2. (A) Representative gel image of endogenous *Fhos* exon 10 with RT-PCR in flies at 15-days-old. Tissue from ten animals per sample. Lower panel, quantification of *Fhos* exon 10 inclusion from three biological replicates. Data are mean ± SD. Two-tailed, unpaired t-test. ***$P = 0.0005$ $(CCTG)_{16}$ versus $(CCTG)_{720}$, ***$P = 0.0007$ $(CCTG)_{720}$ versus $(CCTG)_{720}$ + *gkt RI 1*, ***$P = 0.0003$ $(CCTG)_{720}$ versus $(CCTG)_{720}$ + *gkt RI 2*. (B) Quantification of *Serca* exon 13 expression in flies at 15-days-old from three biological replicates. Data are mean ± SD. Two-tailed, unpaired t-test. ***$P = 0.0002$ $(CCTG)_{16}$ versus $(CCTG)_{720}$, ***$P = 0.0006$ $(CCTG)_{720}$ versus $(CCTG)_{720}$ + *gkt RI 1*, **$P = 0.0026$ $(CCTG)_{720}$ versus $(CCTG)_{720}$ + *gkt RI 2*. (C) Representative gel image of

endogenous *RP49* with RT-PCR in flies at 15-days-old. Tissue from ten animals per sample.

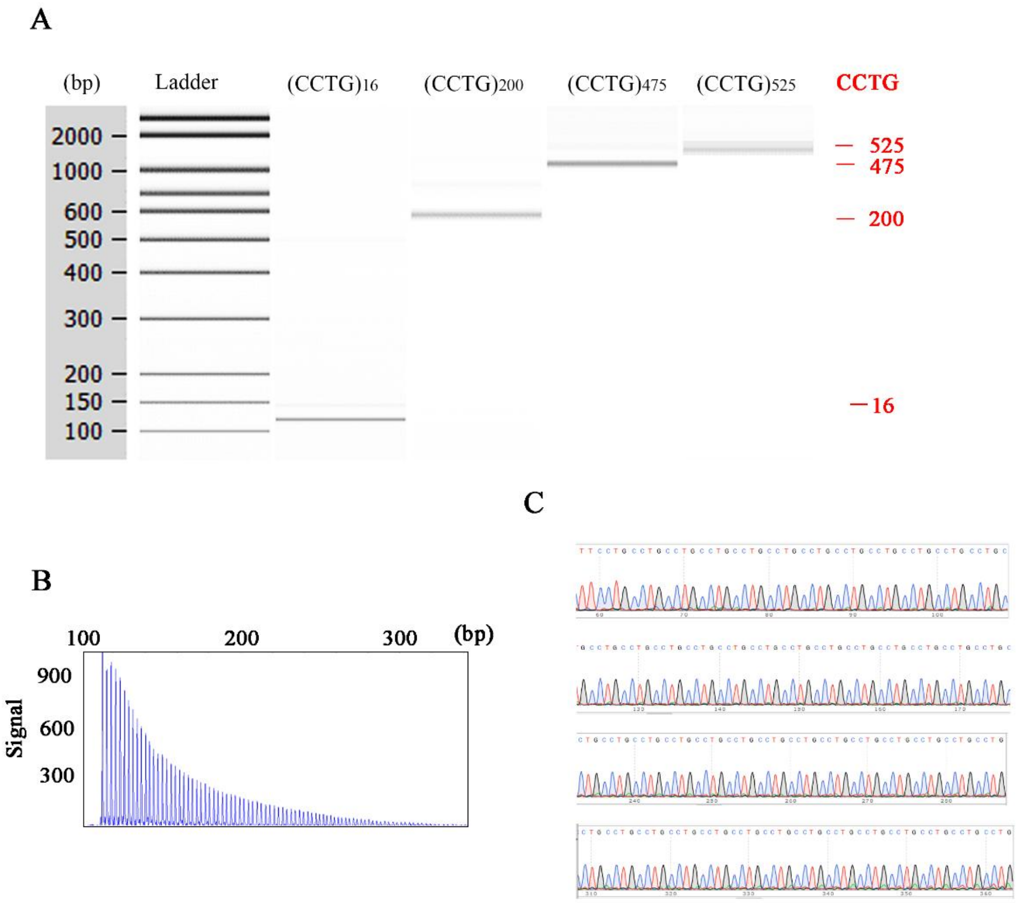

Appendix Figure S5. Validation of the CCTG Repeat Amplification PCR Protocol. (A) Representative gel image of PCR across *UAS-(CCTG)*$_{16}$, *UAS-(CCTG)*$_{200}$, *UAS-(CCTG)*$_{475}$ and *UAS-(CCTG)*$_{525}$ at 1-days-old. Tissue from three animals per sample. (B) Representative capillary electropherogram of CCTG Repeat-Primed PCR of *UAS-(CCTG)*$_{200}$ at 1-days-old. Tissue from three animals per sample. (C) Sanger Sequencing of vector containing (CCTG)$_{475}$.

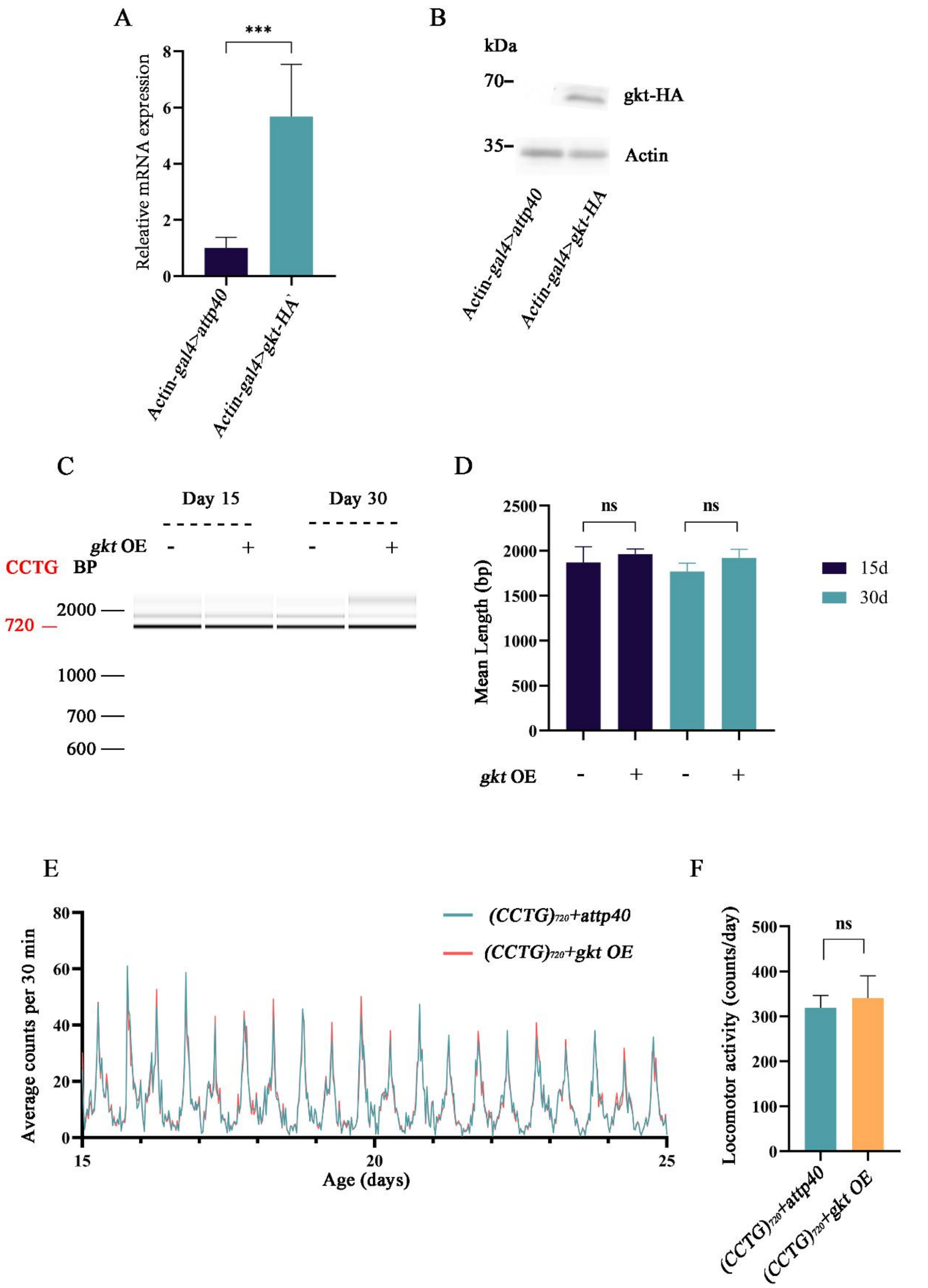

Appendix Figure S6. TDP1/gkt overexpression did not affect CCTG repeat size. (A) Relative mRNA level of *gkt* across different genotypes at 7-days-old. Data are mean ± SD. Two-tailed, unpaired t-test. ****P* = 0.0006. (B) Immunoblot against HA across different genotypes at 7-days-old. (C) Representative gel image of PCR across *Mef2-Gal4>UAS-(CCTG)*$_{720}$ *+attp40* and *Mef2-Gal4>UAS-(CCTG)*$_{720}$*+UAS-gkt-HA* at 15-days-old and 30-days-old. Tissue from three animals per sample. (D) Quantification of mean length of CCTG repeats from three biological replicates.

Data are mean ± SD. Two-tailed, unpaired t-test. (E) The average counts of per 30min in flies across different genotypes from 15-days-old to the 25-days-old. Fifteen animals per sample. (F) Quantification of the average counts per day in flies across different genotypes from 15-days-old to the 25-days-old. Five biological replicates. Data are mean ± SD. Two-tailed, unpaired t-test.

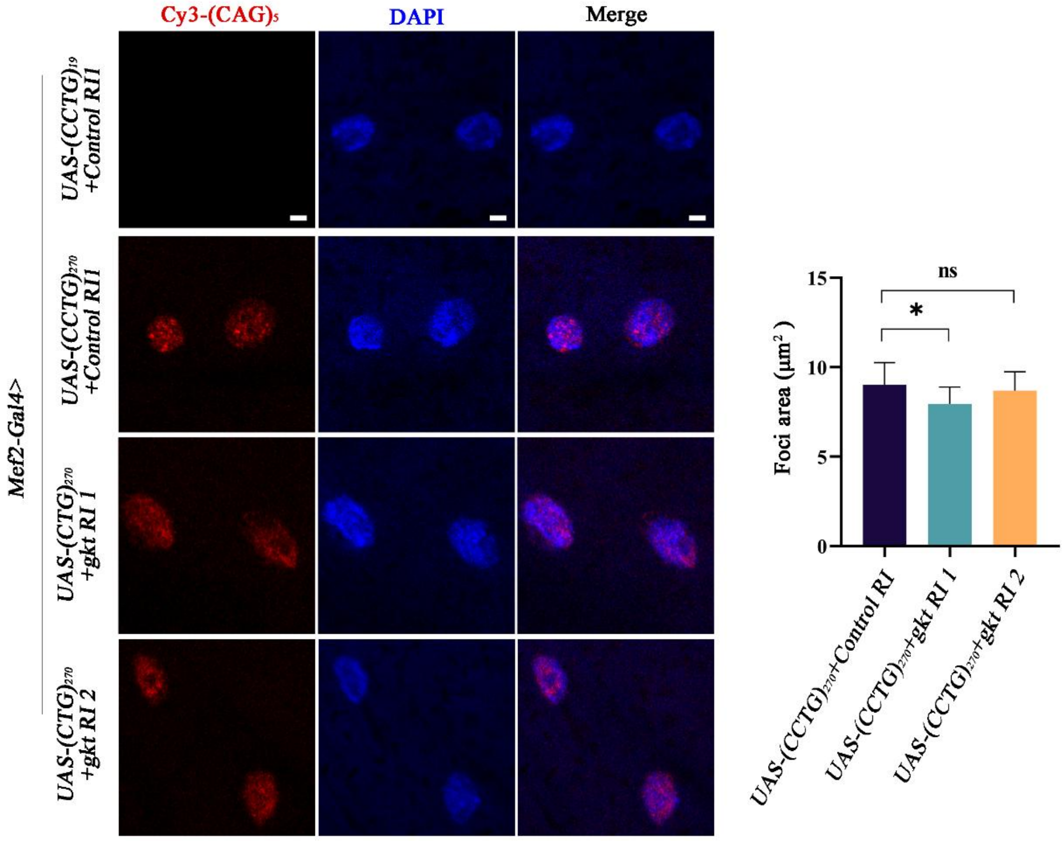

Appendix Figure S7. *TDP1/gkt* knockdown reduced CUG toxic RNA aggregation. (A) Representative Confocal images of CUG-repeat foci with $(CAG)_5$ probe across different genotypes at body-wall muscles of third instar larvae. Scale bars 5 μm. (B) Quantification at least ten animals of each genotype. Data are mean ± SD. Two-tailed, unpaired t-test. *P = 0.0433.
