# Supplementary material for: Modulating CCTG repeat expansion toxicity in DM2 Drosophila model through TDP1 inhibition
Source: EMBO Mol Med. 2025 Mar 25;17(5):967–92. doi: 10.1038/s44321-025-00217-3 (PMC12081759; doi:10.1038/s44321-025-00217-3)
Supplement: Supplementary file 6 — Movie EV1 [file 44321_2025_217_MOESM6_ESM.zip › movie EV1/legend.docx]

Movie EV1

Climbing assay of 15-day-old male fruit flies

1-4, *Mef2-Gal4> (CCTG)_16_ + control RI*, *Mef2-Gal4>(CCTG)_720_ + control RI*, *Mef2-Gal4>(CCTG)_720_ + gkt RI 1*, and *Mef2-Gal4>(CCTG)_720_ + gkt RI 2*.
