# Supplementary figures and images for: Modulating CCTG repeat expansion toxicity in DM2 Drosophila model through TDP1 inhibition
Source: EMBO Mol Med. 2025 Mar 25;17(5):967–92. doi: 10.1038/s44321-025-00217-3 (PMC12081759; doi:10.1038/s44321-025-00217-3)

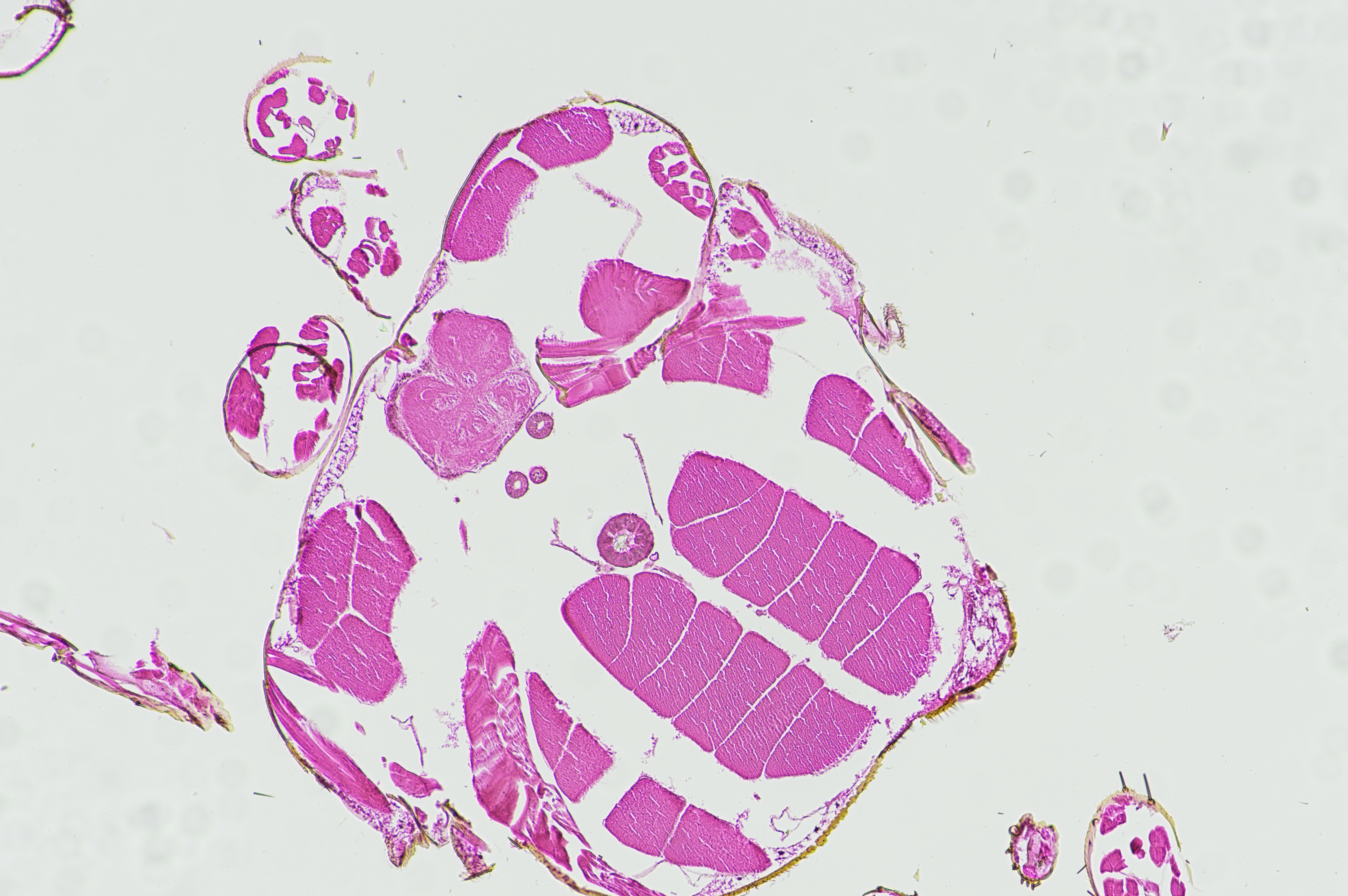

Supplement: Supplementary file 8 — Source data Fig. 2 [file 44321_2025_217_MOESM8_ESM.zip › figure 2/F2 D/16+control RI.tif]

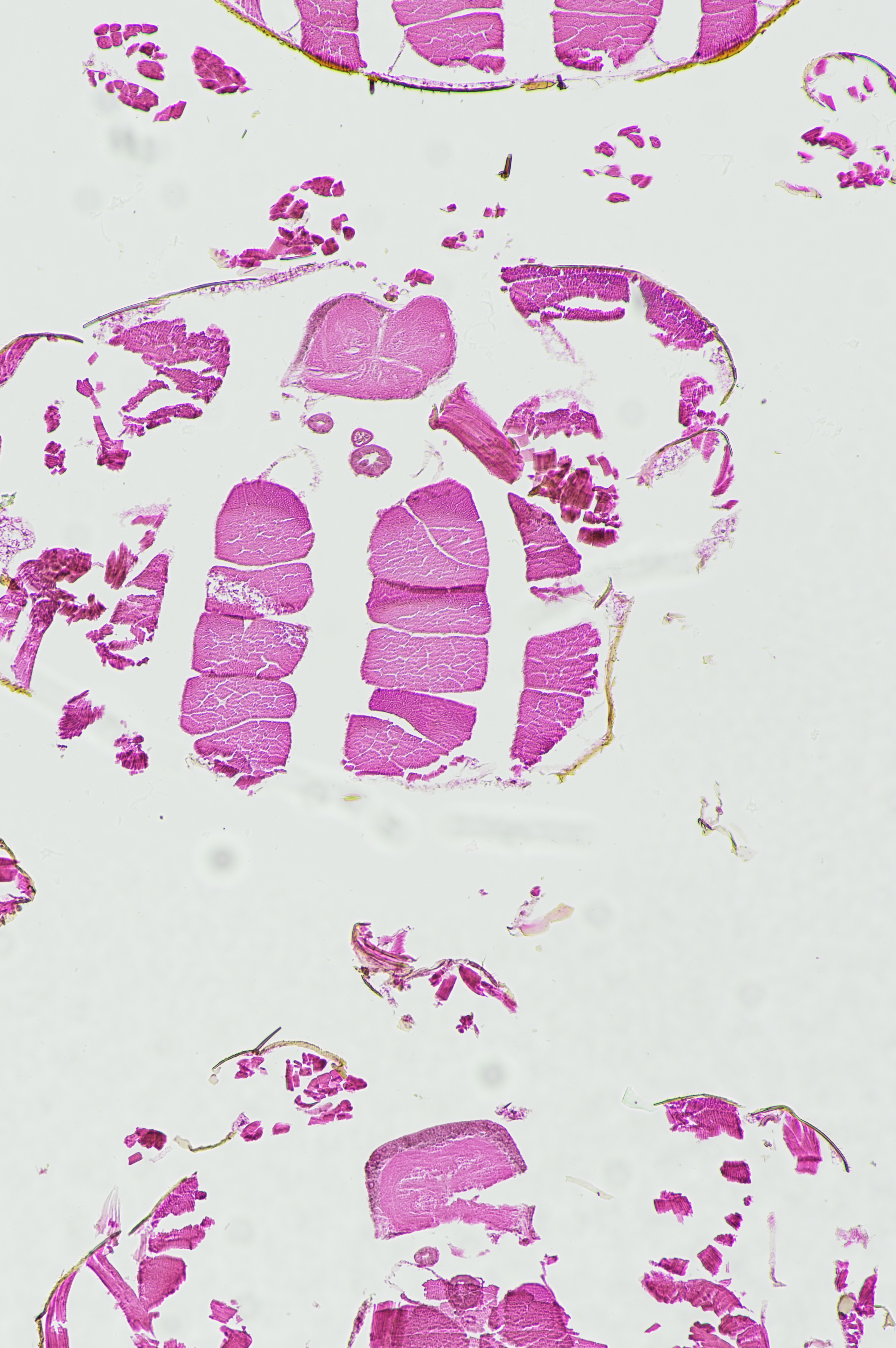

Supplement: Supplementary file 8 — Source data Fig. 2 [file 44321_2025_217_MOESM8_ESM.zip › figure 2/F2 D/720+control RI 2.tif]

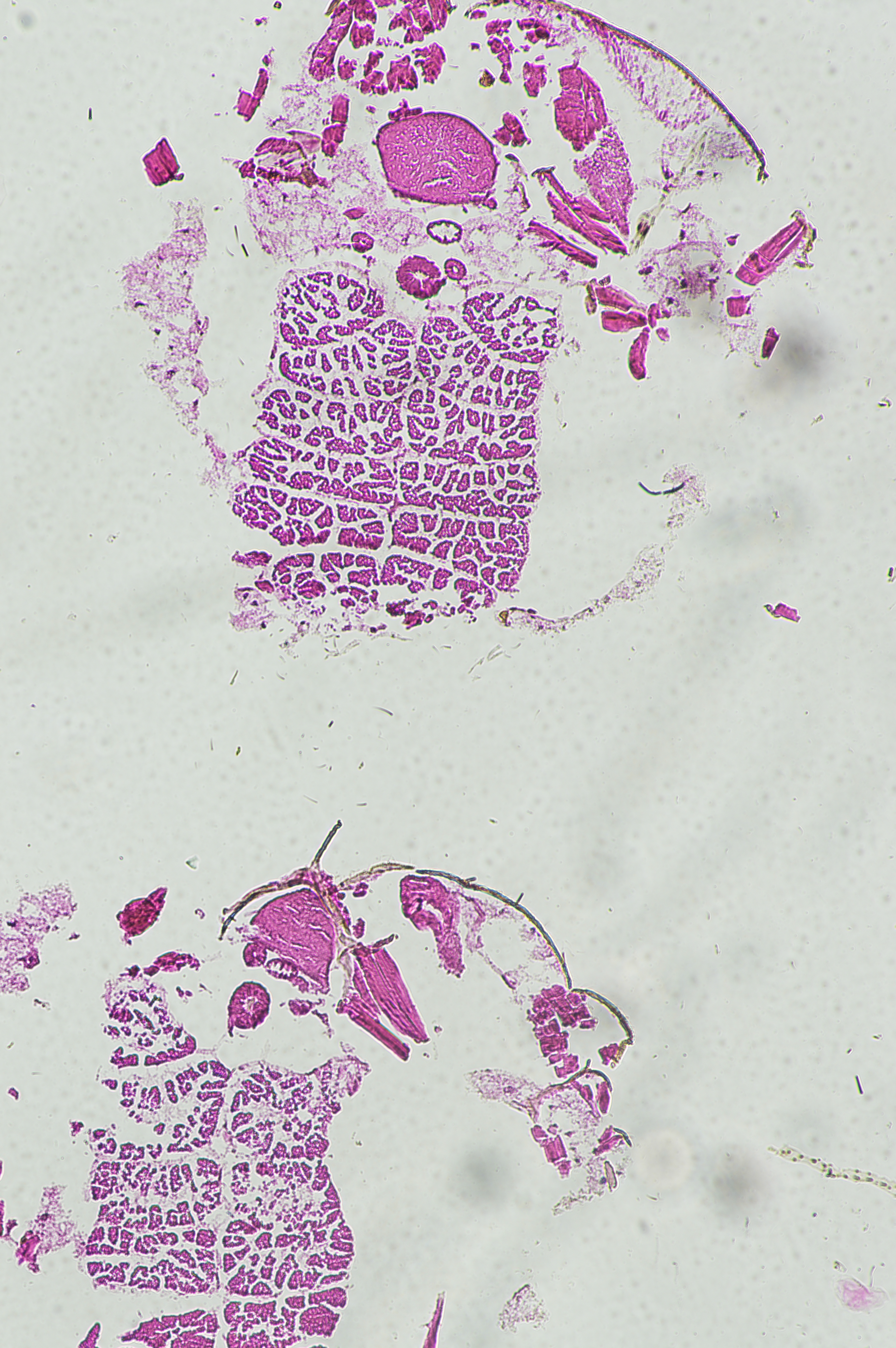

Supplement: Supplementary file 8 — Source data Fig. 2 [file 44321_2025_217_MOESM8_ESM.zip › figure 2/F2 D/720+control RI.tif]

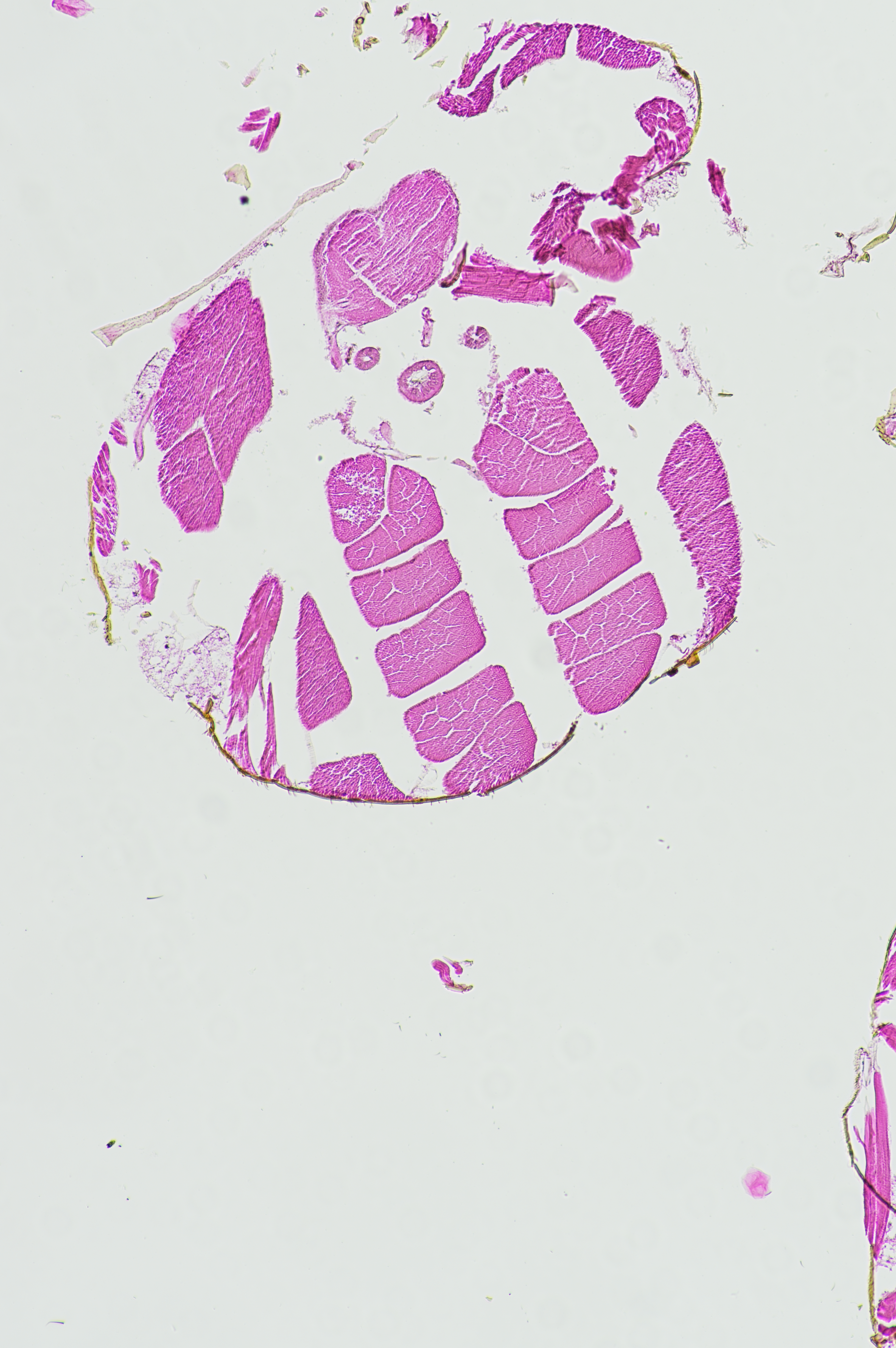

Supplement: Supplementary file 8 — Source data Fig. 2 [file 44321_2025_217_MOESM8_ESM.zip › figure 2/F2 D/720+gkt RI 1.tif]

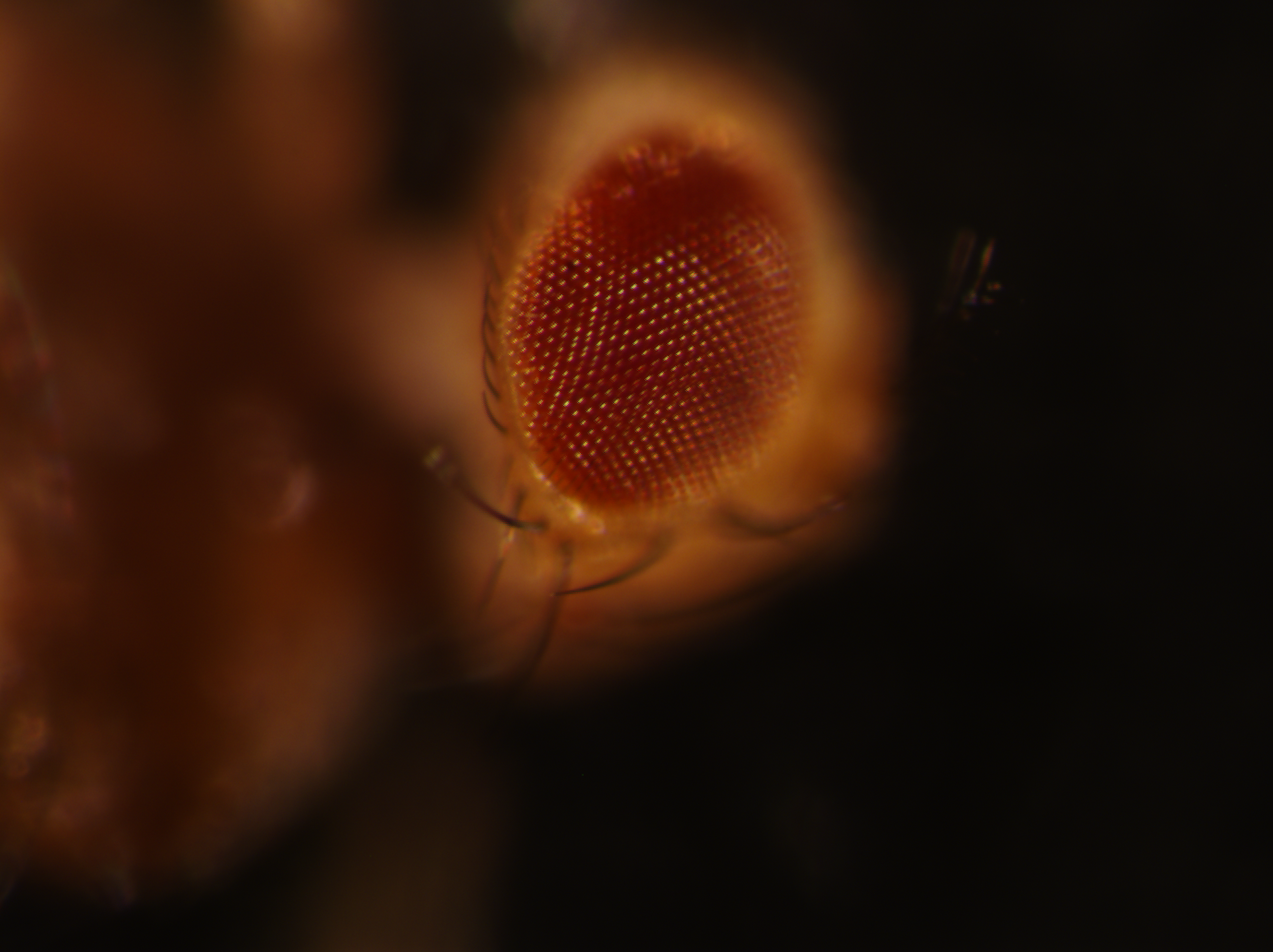

Supplement: Supplementary file 8 — Source data Fig. 2 [file 44321_2025_217_MOESM8_ESM.zip › figure 2/F2 F/16-LM.tif]

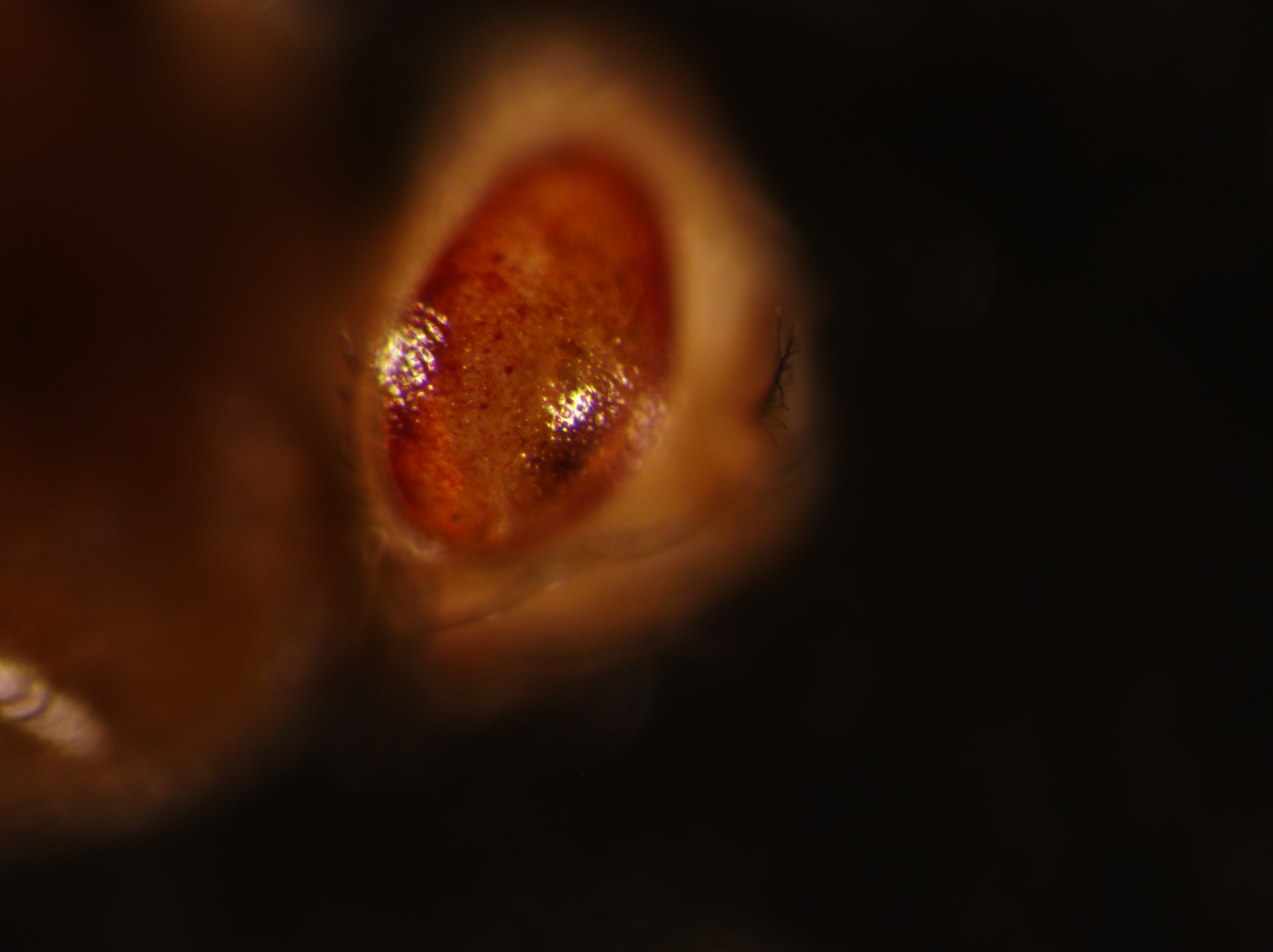

Supplement: Supplementary file 8 — Source data Fig. 2 [file 44321_2025_217_MOESM8_ESM.zip › figure 2/F2 F/720-LM.tif]

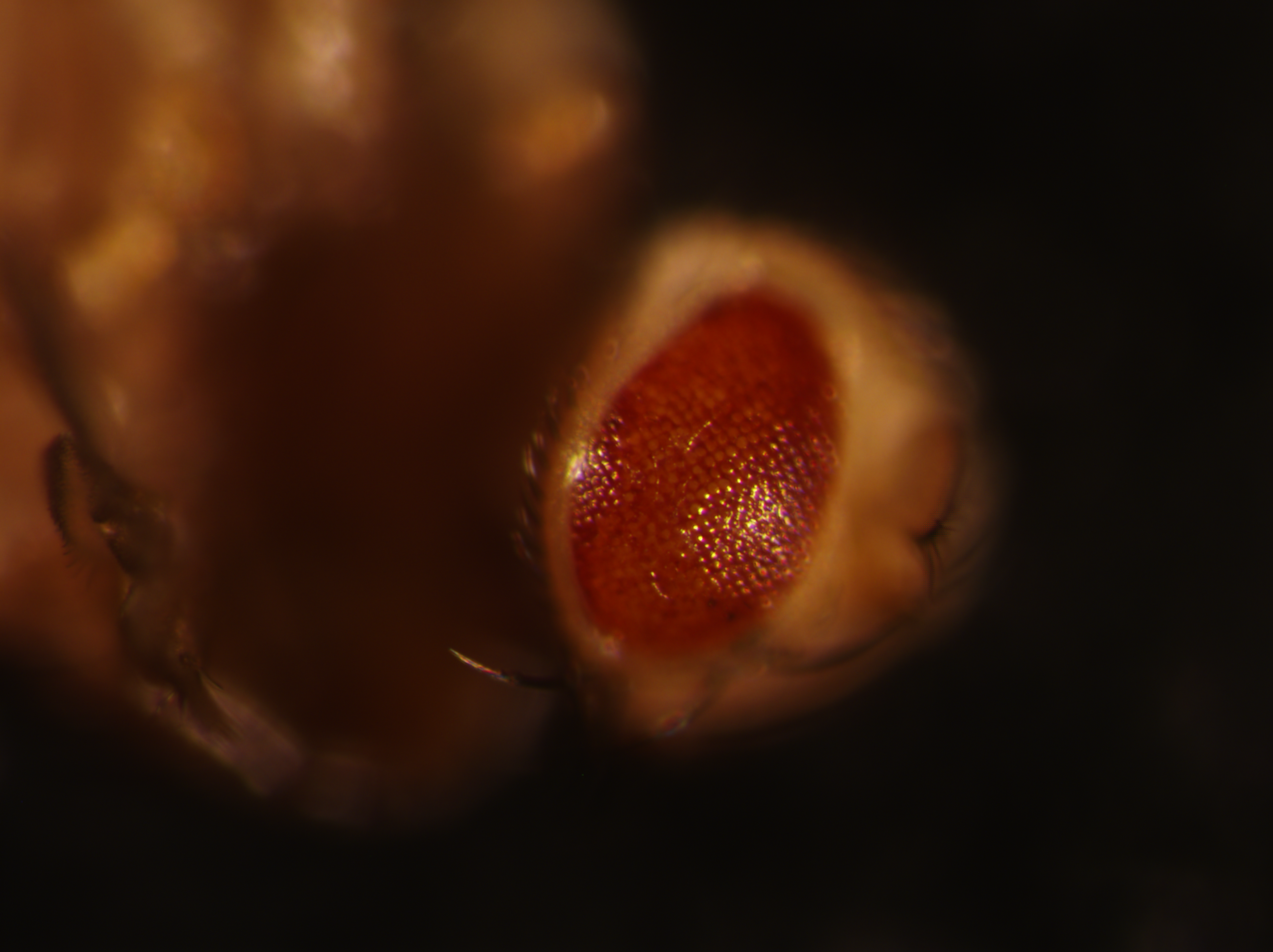

Supplement: Supplementary file 8 — Source data Fig. 2 [file 44321_2025_217_MOESM8_ESM.zip › figure 2/F2 F/gkt RI 1-LM.tif]

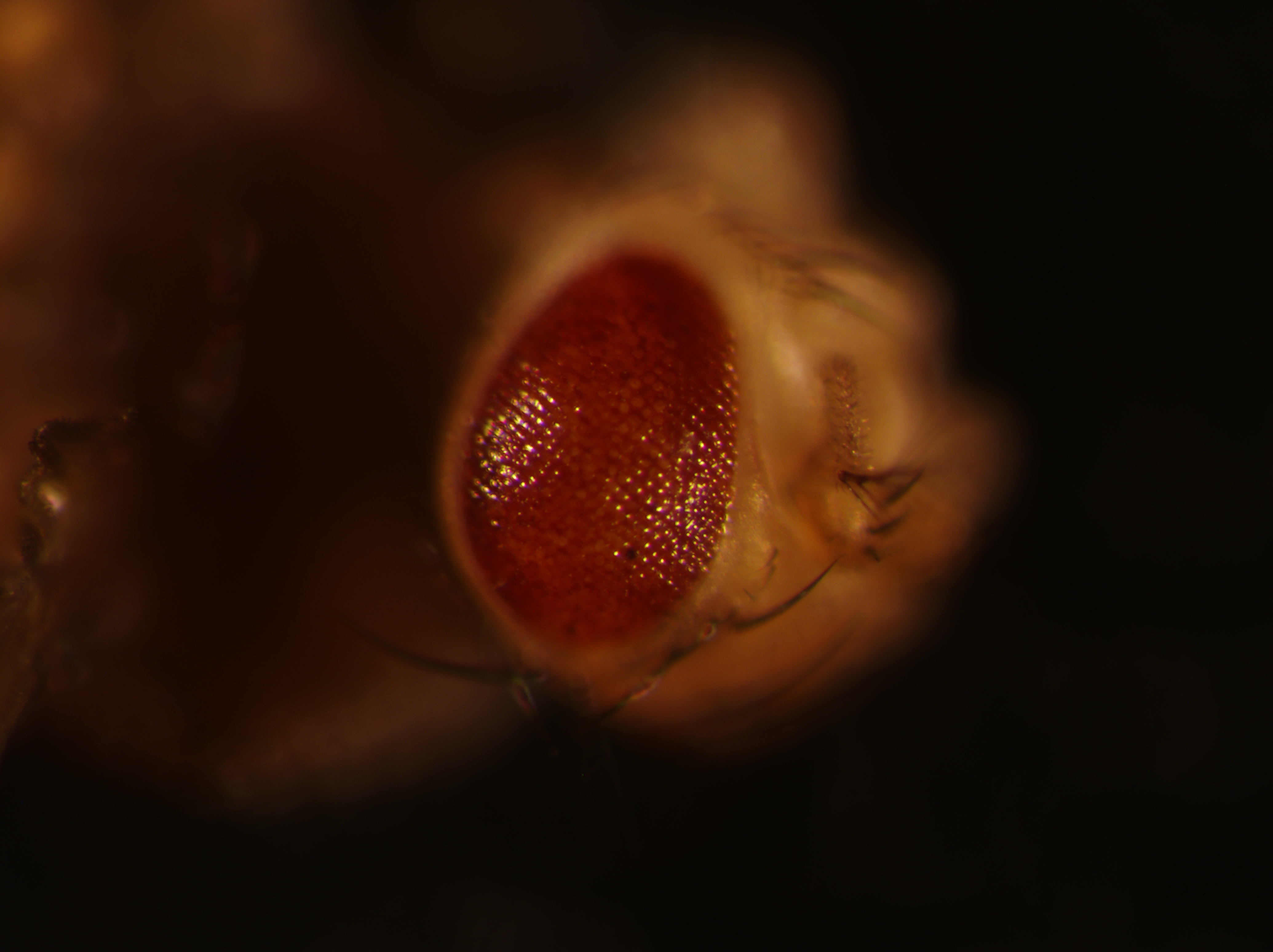

Supplement: Supplementary file 8 — Source data Fig. 2 [file 44321_2025_217_MOESM8_ESM.zip › figure 2/F2 F/gkt RI 2-LM.tif]

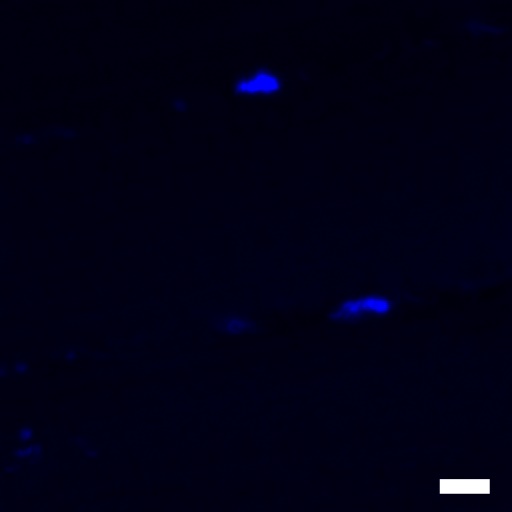

Supplement: Supplementary file 9 — Source data Fig. 3 [file 44321_2025_217_MOESM9_ESM.zip › figure 3/F3 A/16-DAPI.jpg]

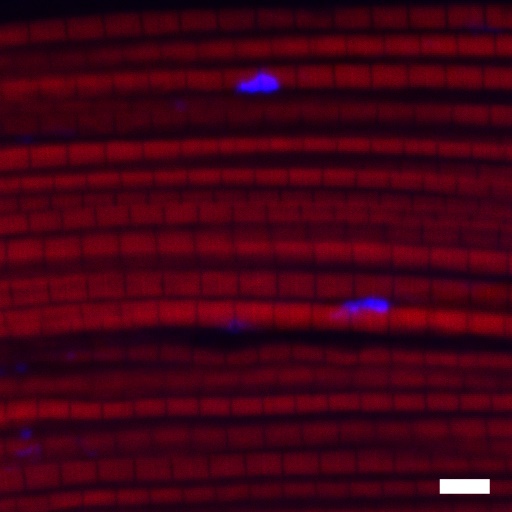

Supplement: Supplementary file 9 — Source data Fig. 3 [file 44321_2025_217_MOESM9_ESM.zip › figure 3/F3 A/16-pha+DAPI.jpg]

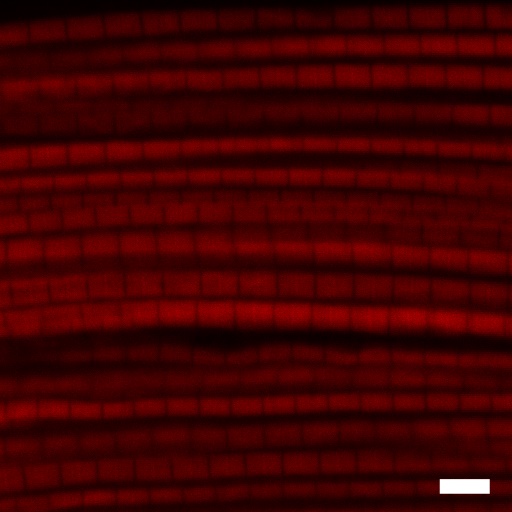

Supplement: Supplementary file 9 — Source data Fig. 3 [file 44321_2025_217_MOESM9_ESM.zip › figure 3/F3 A/16-pha.jpg]

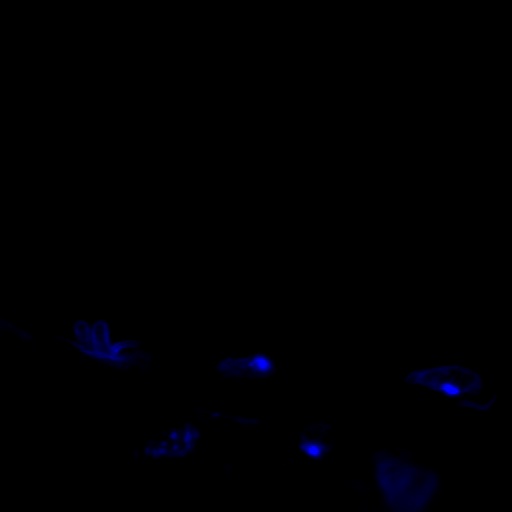

Supplement: Supplementary file 9 — Source data Fig. 3 [file 44321_2025_217_MOESM9_ESM.zip › figure 3/F3 A/720-DAPI.jpg]

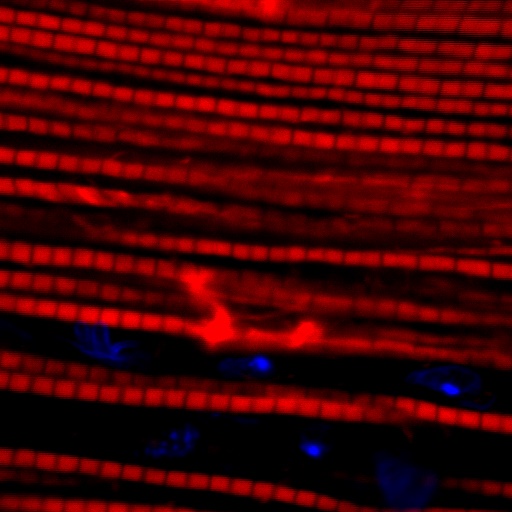

Supplement: Supplementary file 9 — Source data Fig. 3 [file 44321_2025_217_MOESM9_ESM.zip › figure 3/F3 A/720-pha+DAPI.jpg]

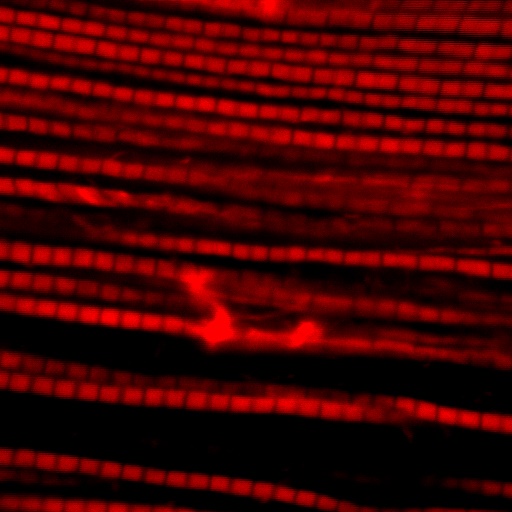

Supplement: Supplementary file 9 — Source data Fig. 3 [file 44321_2025_217_MOESM9_ESM.zip › figure 3/F3 A/720-pha.jpg]

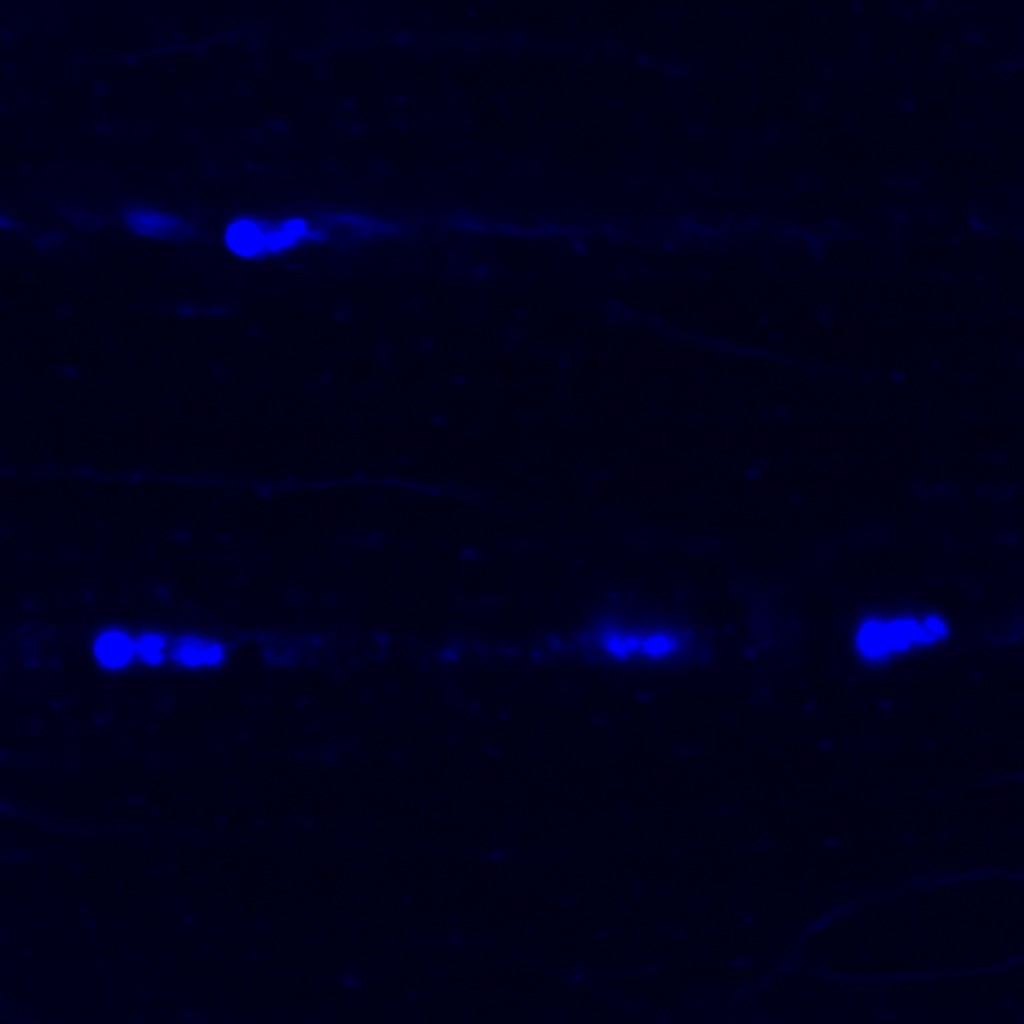

Supplement: Supplementary file 9 — Source data Fig. 3 [file 44321_2025_217_MOESM9_ESM.zip › figure 3/F3 A/gkt RI 1-DAPI.jpg]

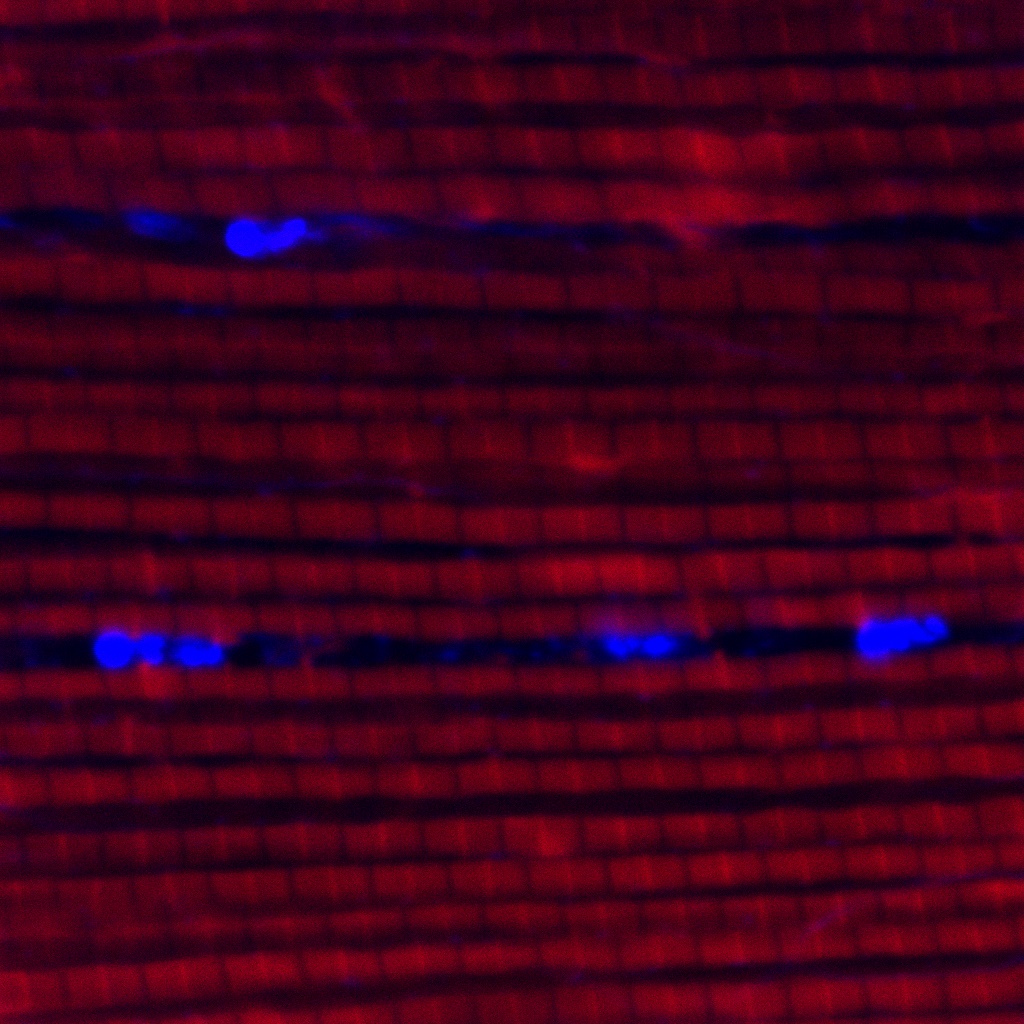

Supplement: Supplementary file 9 — Source data Fig. 3 [file 44321_2025_217_MOESM9_ESM.zip › figure 3/F3 A/gkt RI 1-pha+DAPI.jpg]

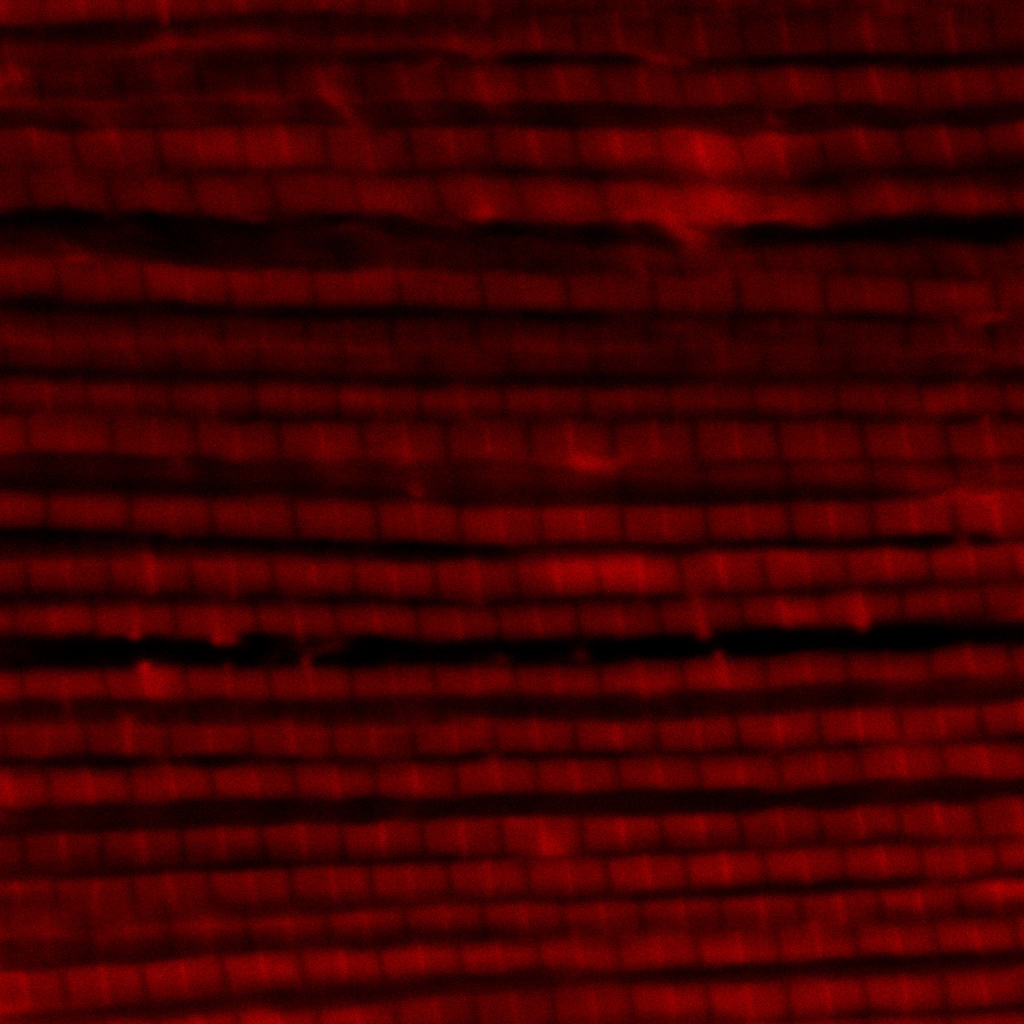

Supplement: Supplementary file 9 — Source data Fig. 3 [file 44321_2025_217_MOESM9_ESM.zip › figure 3/F3 A/gkt RI 1-pha.jpg]

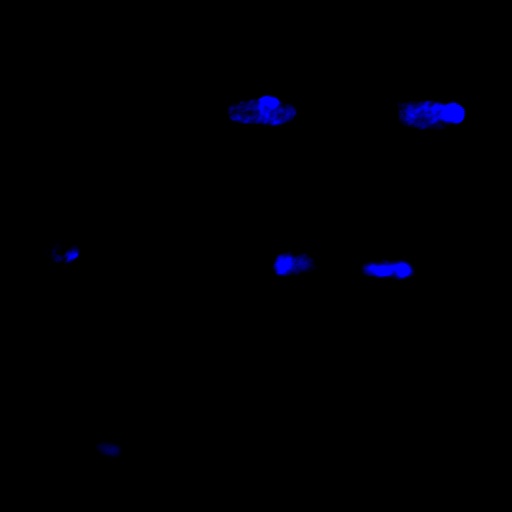

Supplement: Supplementary file 9 — Source data Fig. 3 [file 44321_2025_217_MOESM9_ESM.zip › figure 3/F3 A/gkt RI 2-DAPI.jpg]

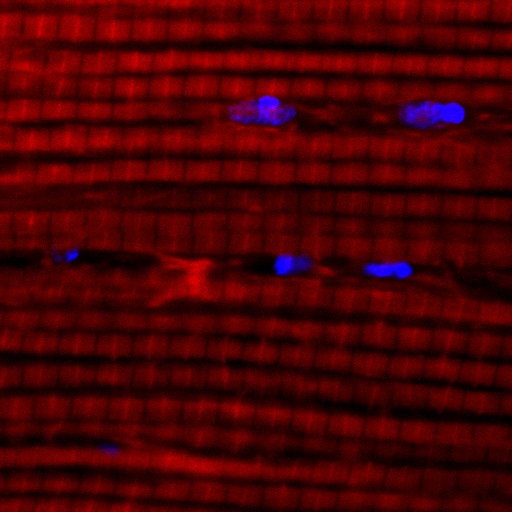

Supplement: Supplementary file 9 — Source data Fig. 3 [file 44321_2025_217_MOESM9_ESM.zip › figure 3/F3 A/gkt RI 2-pha+DAPI.jpg]

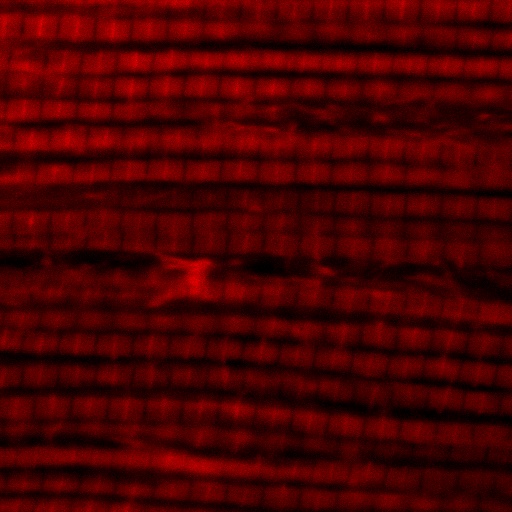

Supplement: Supplementary file 9 — Source data Fig. 3 [file 44321_2025_217_MOESM9_ESM.zip › figure 3/F3 A/gkt RI 2-pha.jpg]

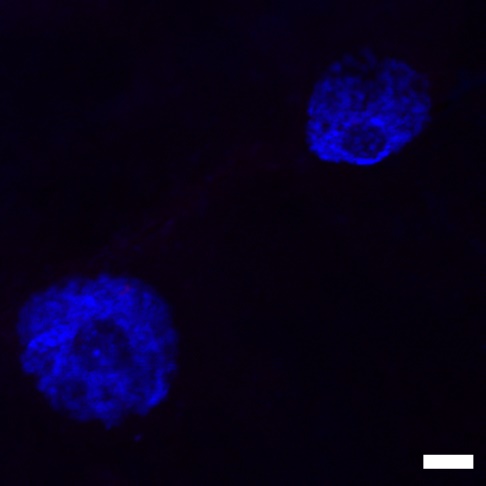

Supplement: Supplementary file 9 — Source data Fig. 3 [file 44321_2025_217_MOESM9_ESM.zip › figure 3/F3 C/16_CY3+DAPI.jpg]

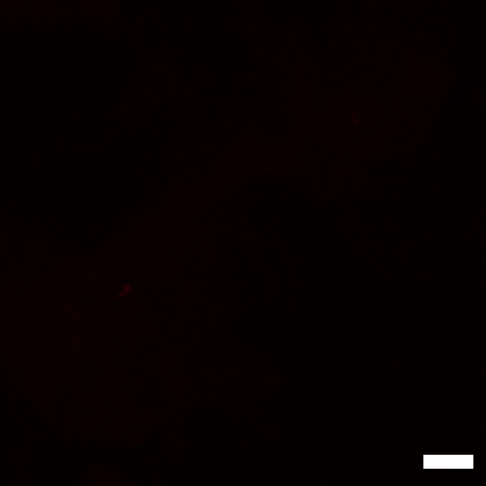

Supplement: Supplementary file 9 — Source data Fig. 3 [file 44321_2025_217_MOESM9_ESM.zip › figure 3/F3 C/16_CY3.jpg]

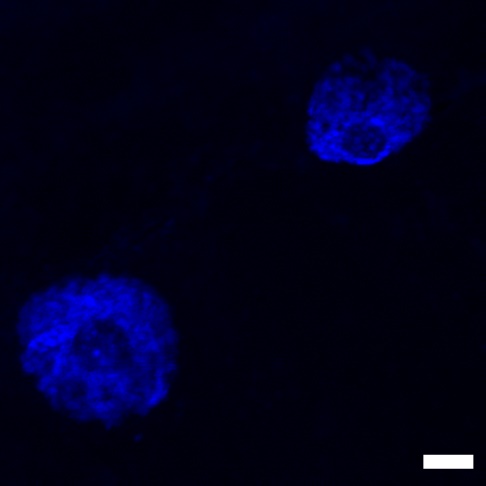

Supplement: Supplementary file 9 — Source data Fig. 3 [file 44321_2025_217_MOESM9_ESM.zip › figure 3/F3 C/16_DAPI.jpg]

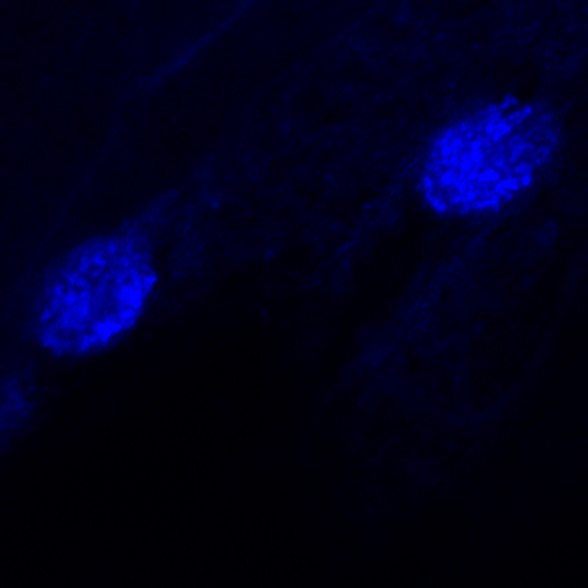

Supplement: Supplementary file 9 — Source data Fig. 3 [file 44321_2025_217_MOESM9_ESM.zip › figure 3/F3 C/720_ CY3+DAPI.jpg]

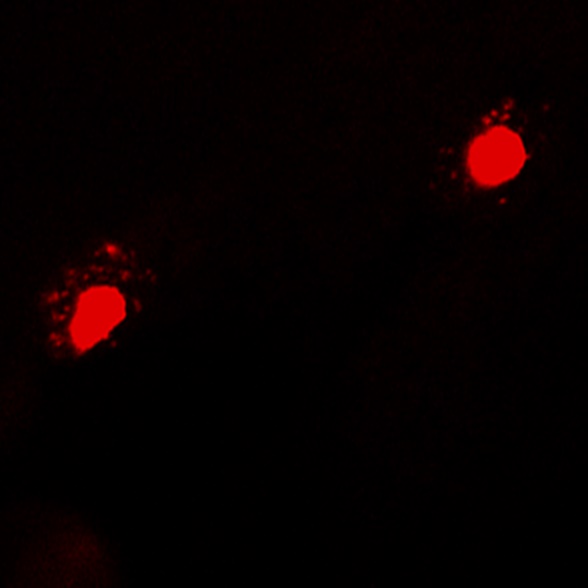

Supplement: Supplementary file 9 — Source data Fig. 3 [file 44321_2025_217_MOESM9_ESM.zip › figure 3/F3 C/720_ CY3.jpg]

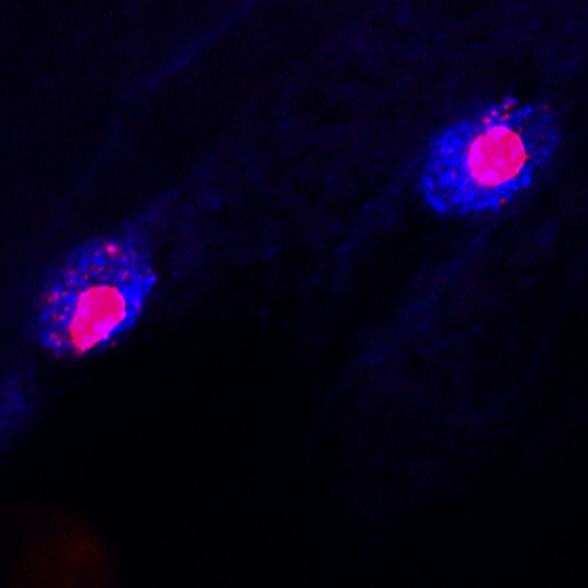

Supplement: Supplementary file 9 — Source data Fig. 3 [file 44321_2025_217_MOESM9_ESM.zip › figure 3/F3 C/720_ DAPI.jpg]

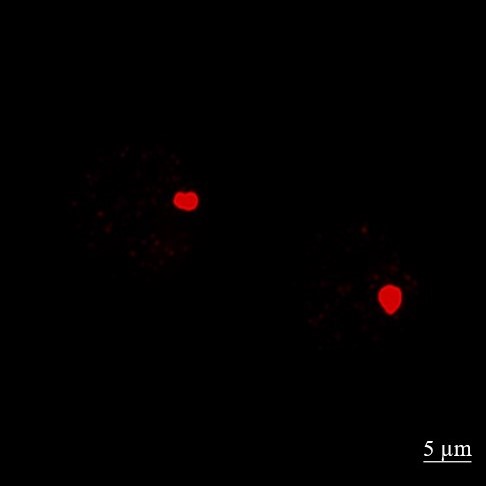

Supplement: Supplementary file 9 — Source data Fig. 3 [file 44321_2025_217_MOESM9_ESM.zip › figure 3/F3 C/gkt RI 1_CY3.jpg]

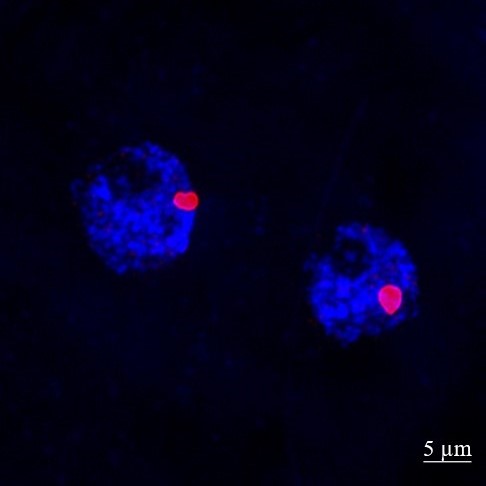

Supplement: Supplementary file 9 — Source data Fig. 3 [file 44321_2025_217_MOESM9_ESM.zip › figure 3/F3 C/gkt RI 1_DAPI+CY3.jpg]

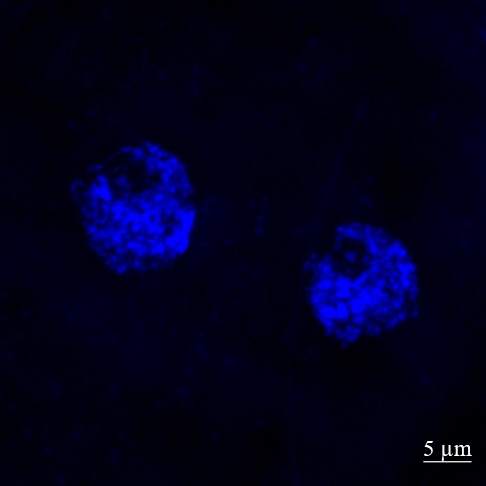

Supplement: Supplementary file 9 — Source data Fig. 3 [file 44321_2025_217_MOESM9_ESM.zip › figure 3/F3 C/gkt RI 1_DAPI.jpg]

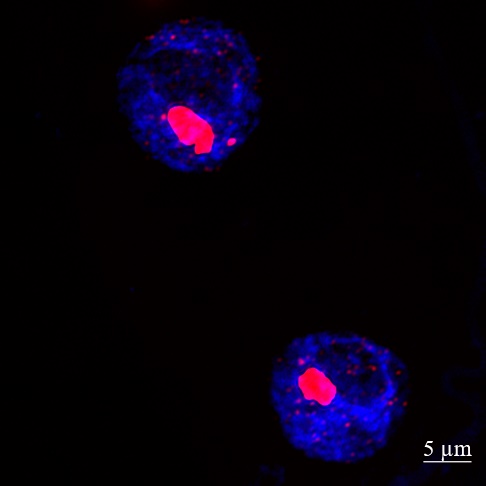

Supplement: Supplementary file 9 — Source data Fig. 3 [file 44321_2025_217_MOESM9_ESM.zip › figure 3/F3 C/gkt RI 2_CY3+DAPI.jpg]

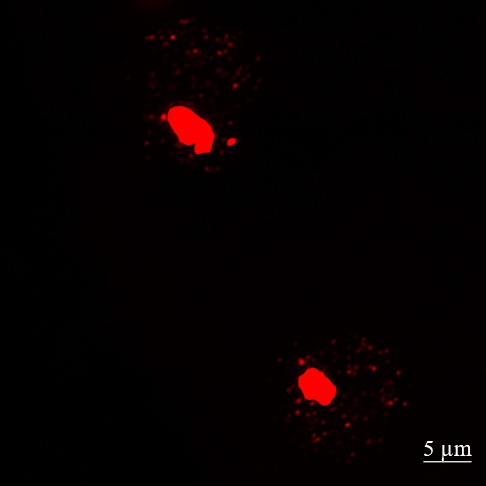

Supplement: Supplementary file 9 — Source data Fig. 3 [file 44321_2025_217_MOESM9_ESM.zip › figure 3/F3 C/gkt RI 2_CY3.jpg]

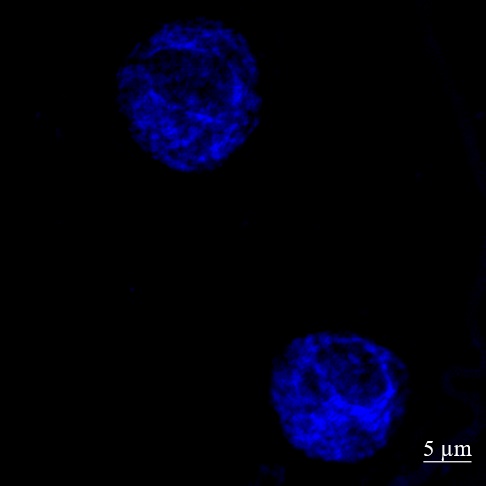

Supplement: Supplementary file 9 — Source data Fig. 3 [file 44321_2025_217_MOESM9_ESM.zip › figure 3/F3 C/gkt RI 2_DAPI.jpg]

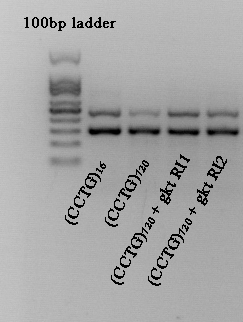

Supplement: Supplementary file 9 — Source data Fig. 3 [file 44321_2025_217_MOESM9_ESM.zip › figure 3/F3 E/F3 E upper.tif]

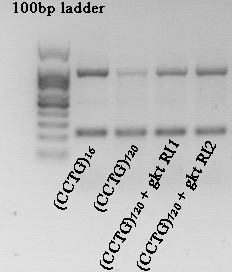

Supplement: Supplementary file 9 — Source data Fig. 3 [file 44321_2025_217_MOESM9_ESM.zip › figure 3/F3 F/F3 F upper.tif]

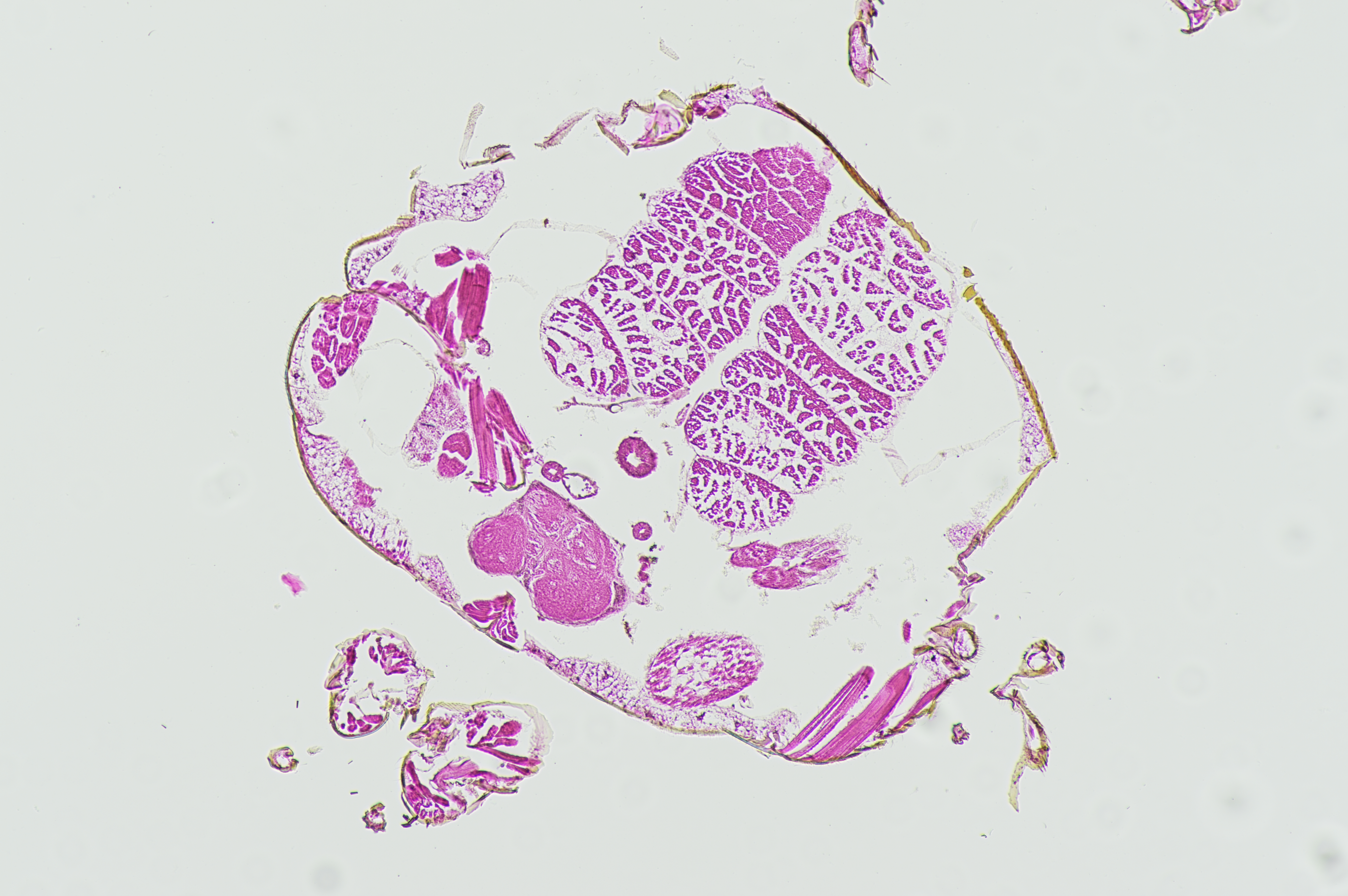

Supplement: Supplementary file 12 — Source data Fig. 6 [file 44321_2025_217_MOESM12_ESM.zip › figure 6/F6 D/Amikacin.tif]

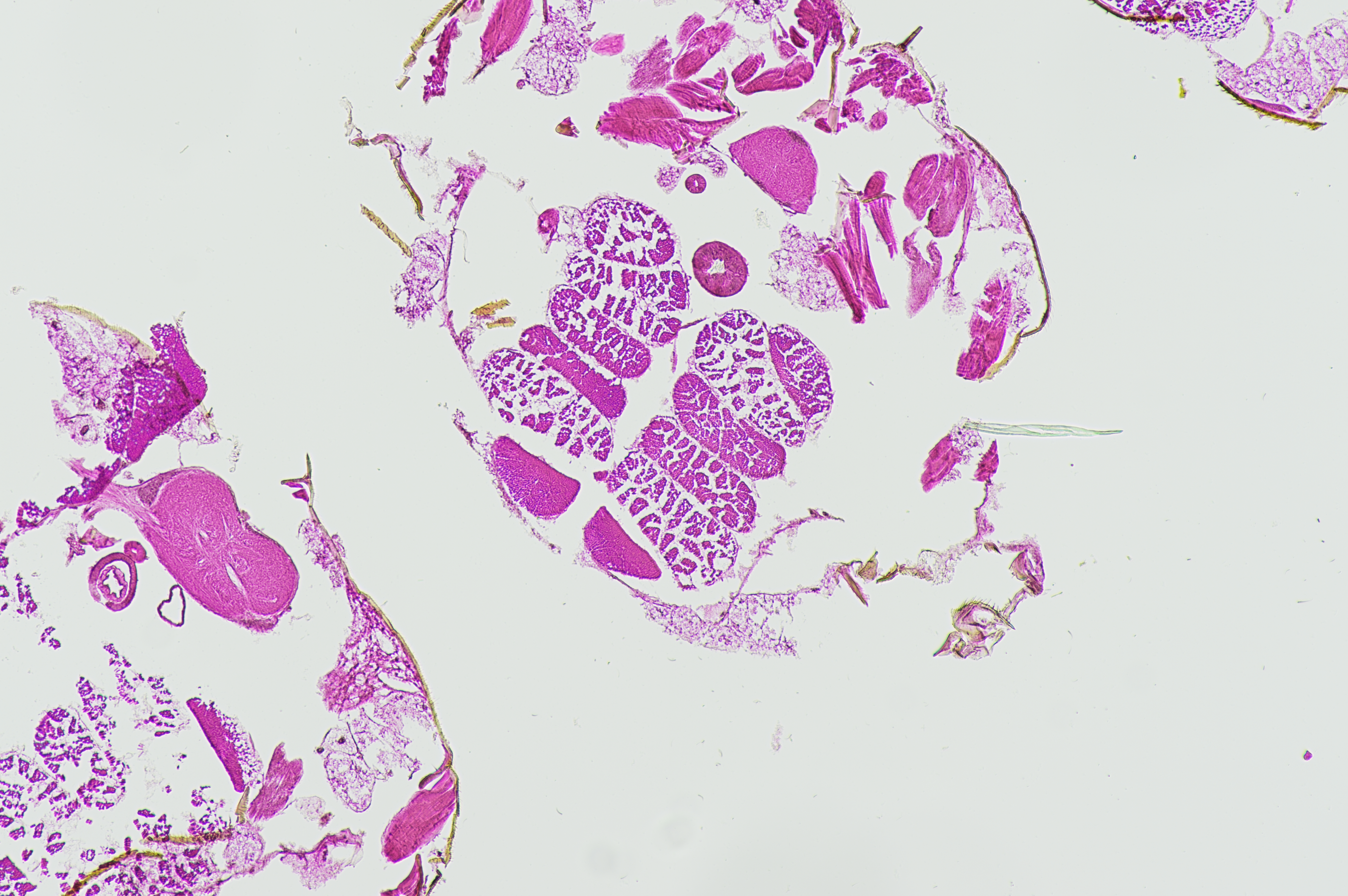

Supplement: Supplementary file 12 — Source data Fig. 6 [file 44321_2025_217_MOESM12_ESM.zip › figure 6/F6 D/CPT.tif]

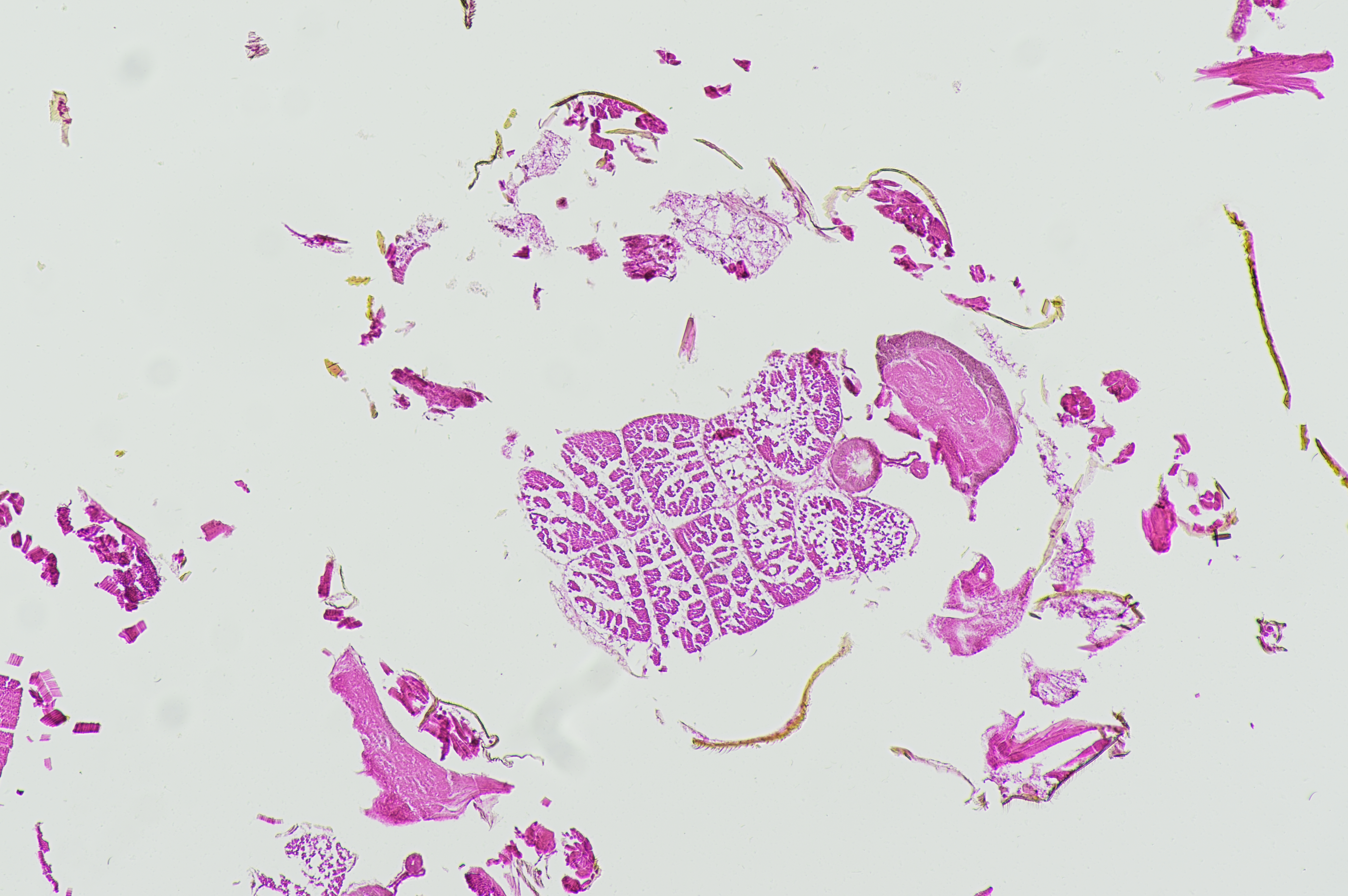

Supplement: Supplementary file 12 — Source data Fig. 6 [file 44321_2025_217_MOESM12_ESM.zip › figure 6/F6 D/DMSO.tif]

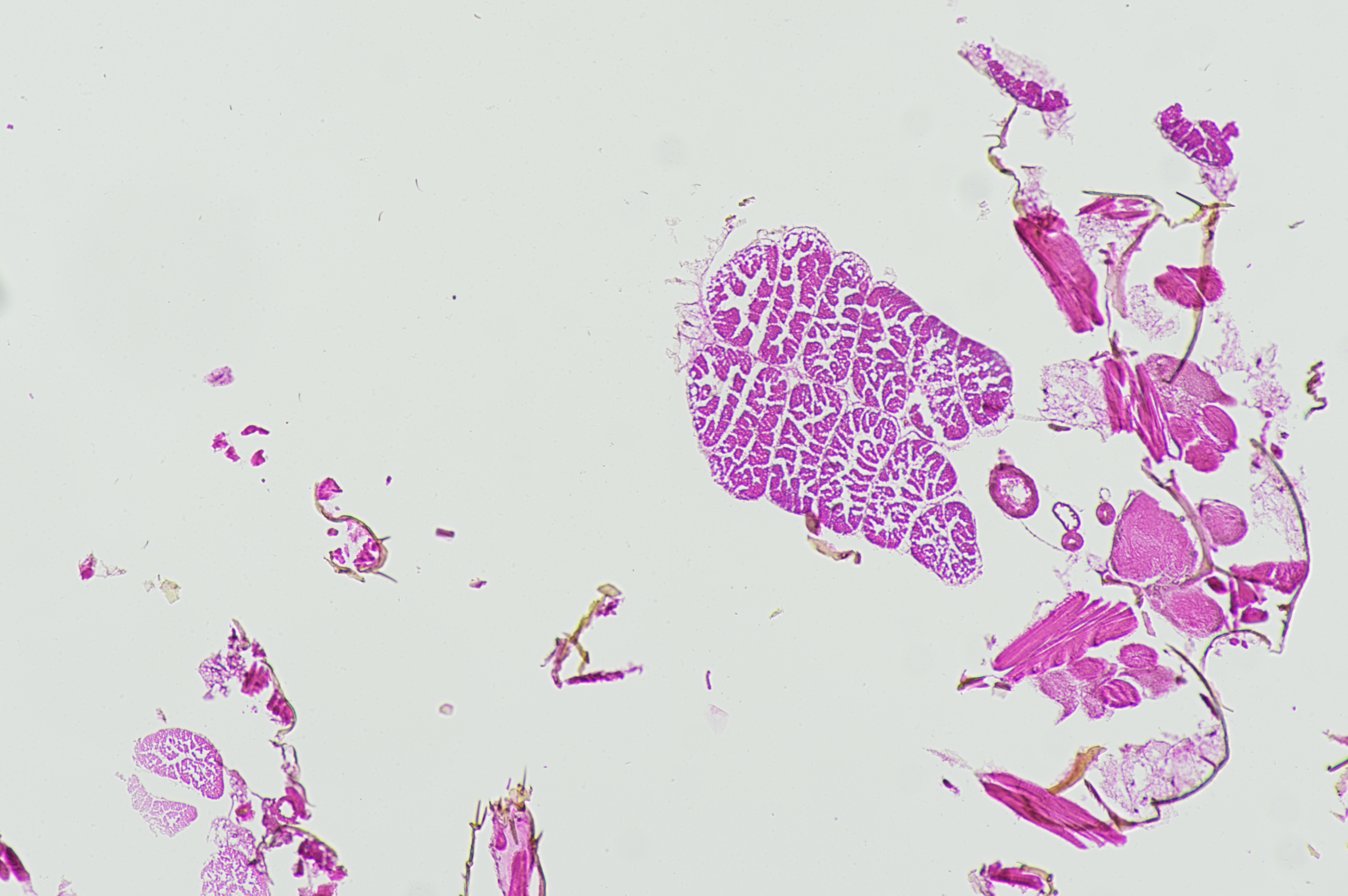

Supplement: Supplementary file 12 — Source data Fig. 6 [file 44321_2025_217_MOESM12_ESM.zip › figure 6/F6 D/Lawsone.tif]

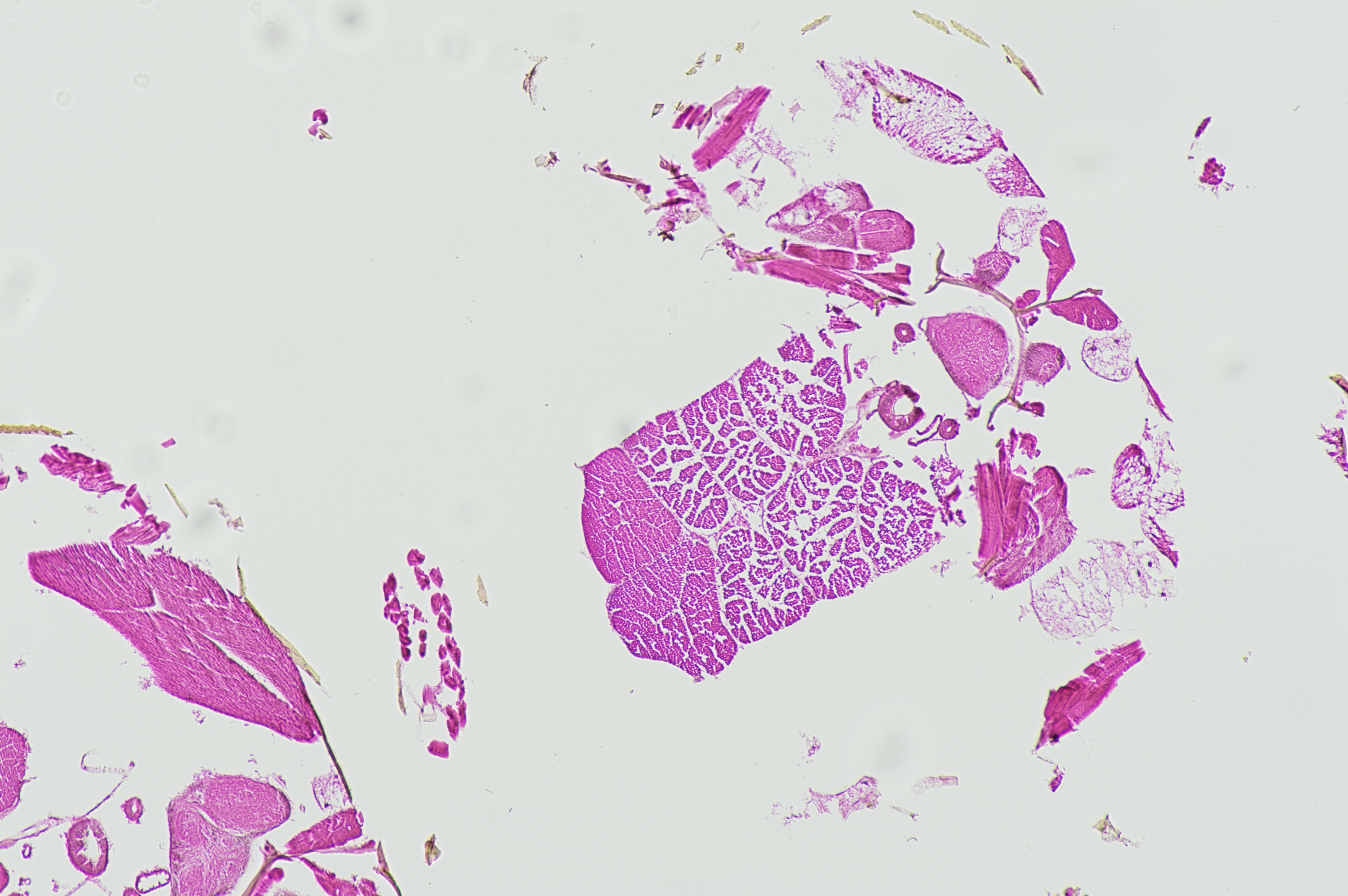

Supplement: Supplementary file 12 — Source data Fig. 6 [file 44321_2025_217_MOESM12_ESM.zip › figure 6/F6 D/SPI-112.tif]

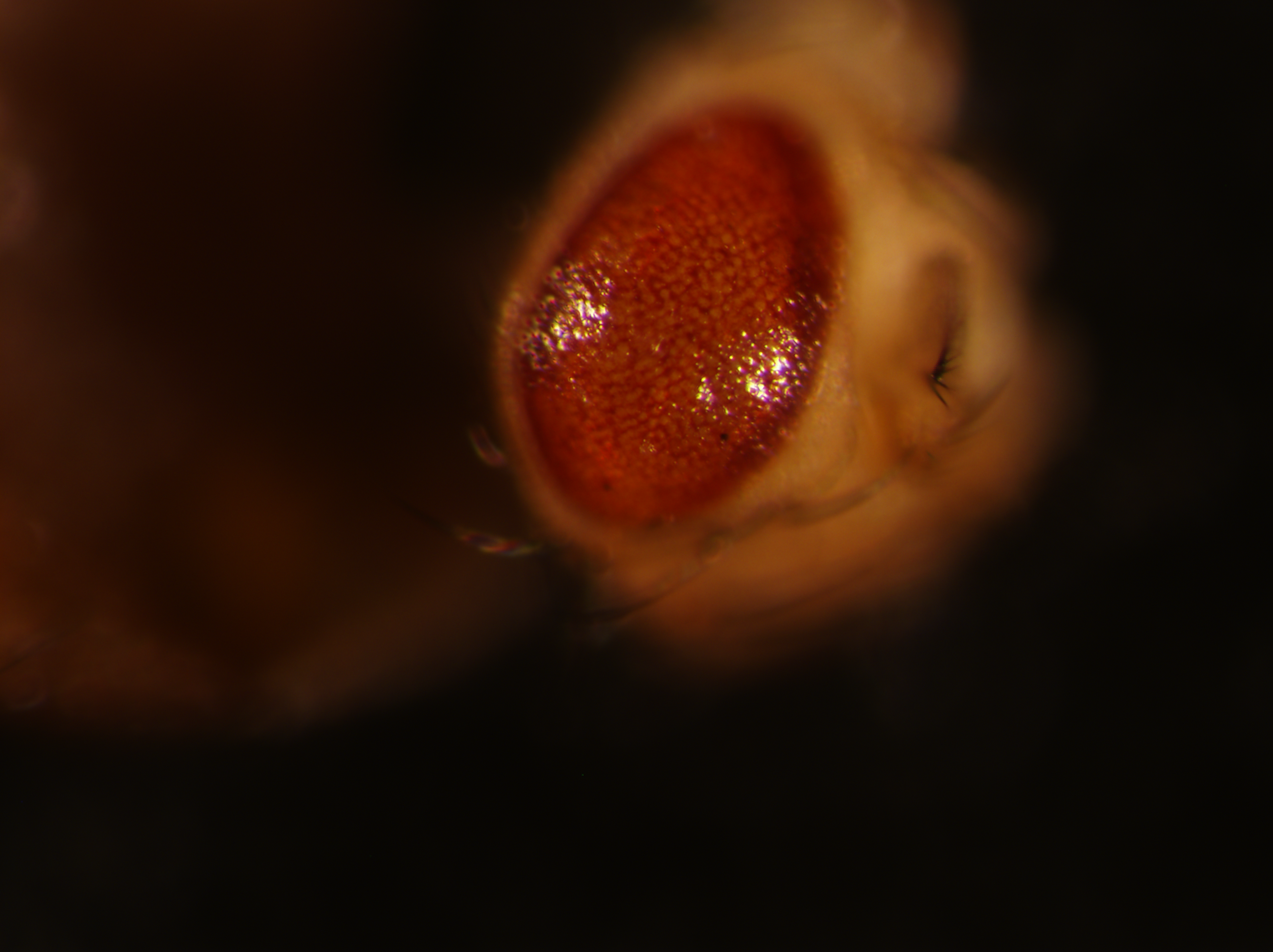

Supplement: Supplementary file 12 — Source data Fig. 6 [file 44321_2025_217_MOESM12_ESM.zip › figure 6/F6 F/Amikacin-LM.tif]

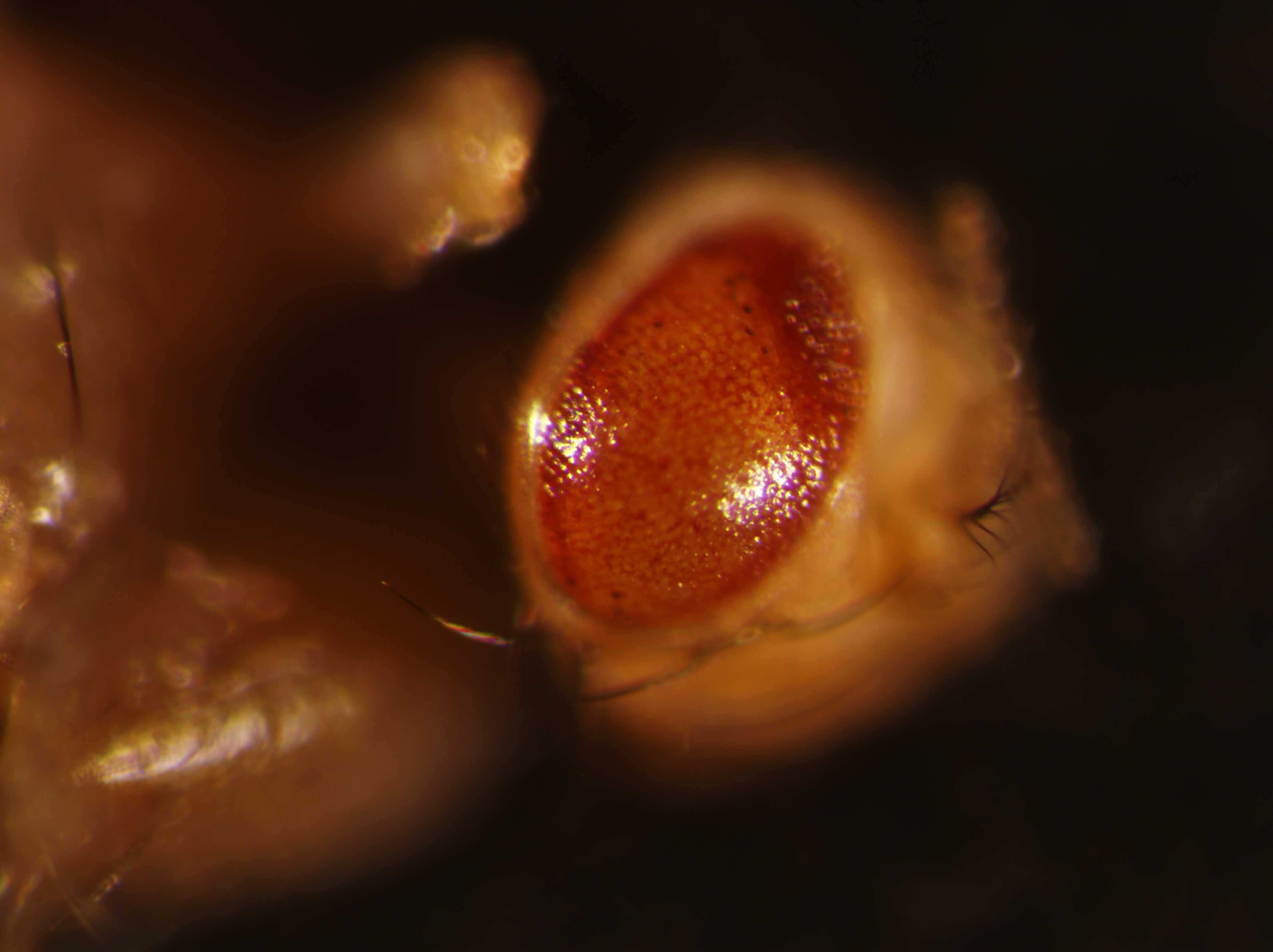

Supplement: Supplementary file 12 — Source data Fig. 6 [file 44321_2025_217_MOESM12_ESM.zip › figure 6/F6 F/CPT-LM.tif]

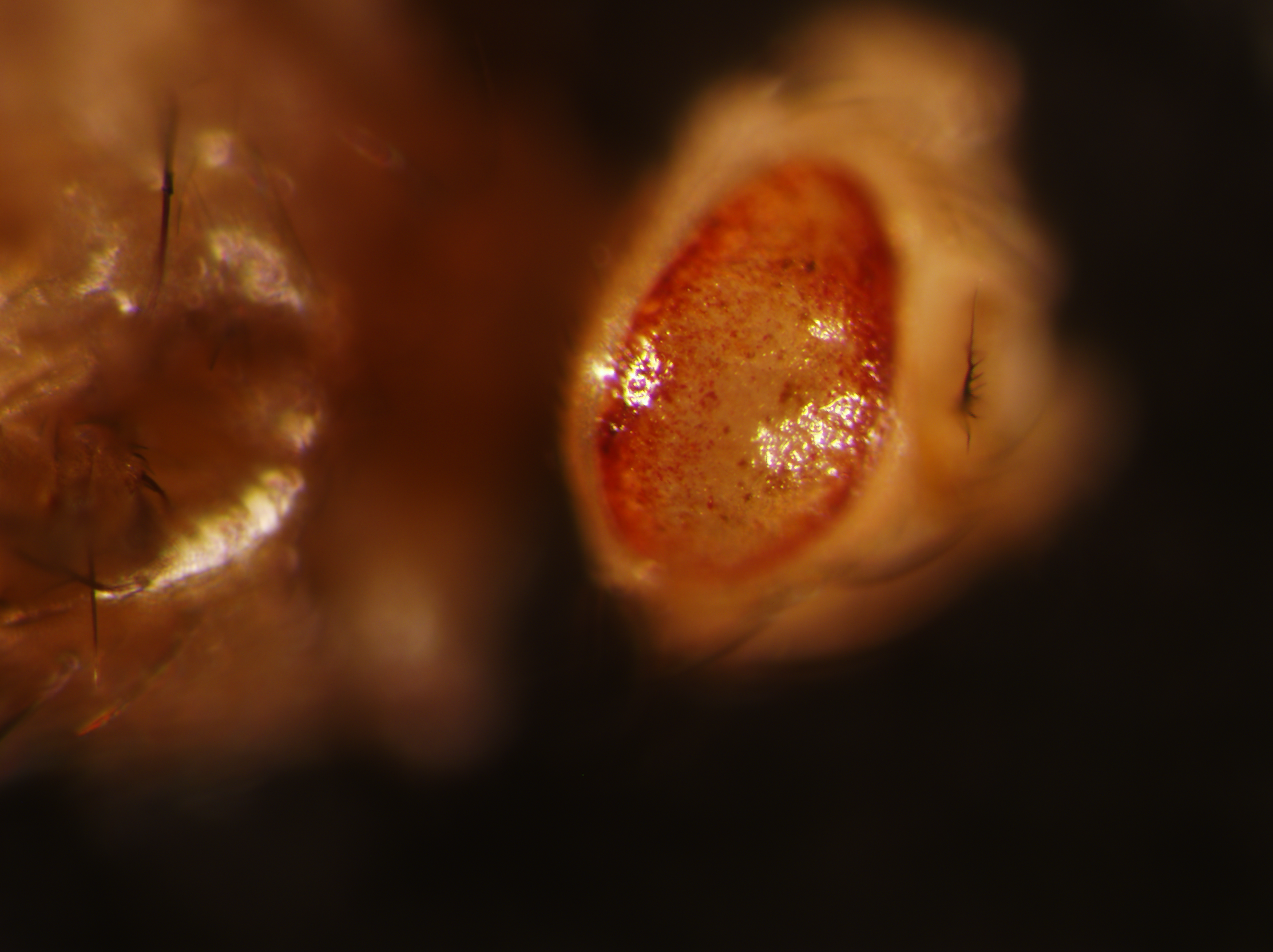

Supplement: Supplementary file 12 — Source data Fig. 6 [file 44321_2025_217_MOESM12_ESM.zip › figure 6/F6 F/DMSO-LM.tif]

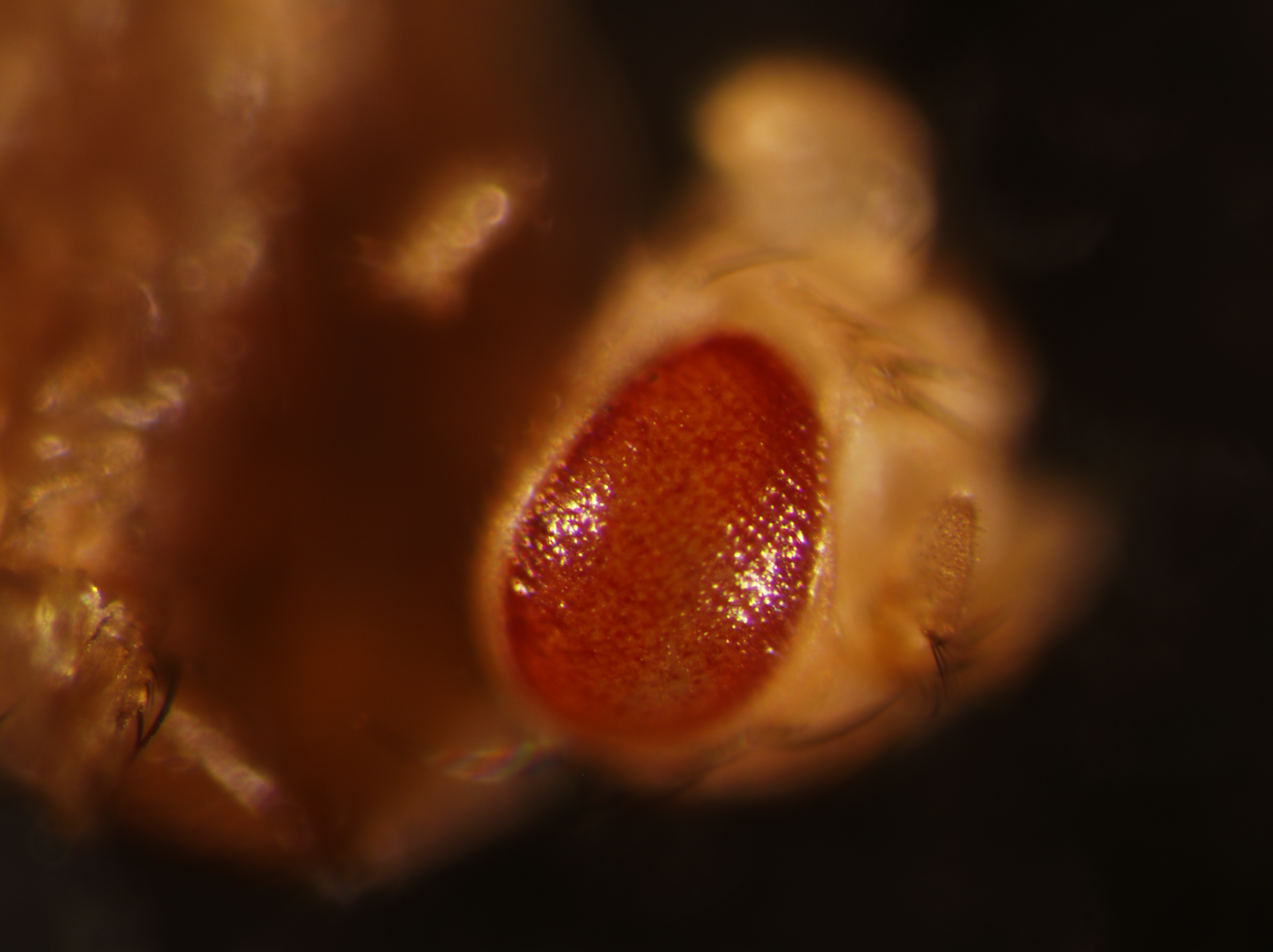

Supplement: Supplementary file 12 — Source data Fig. 6 [file 44321_2025_217_MOESM12_ESM.zip › figure 6/F6 F/Lawsone-LM.tif]

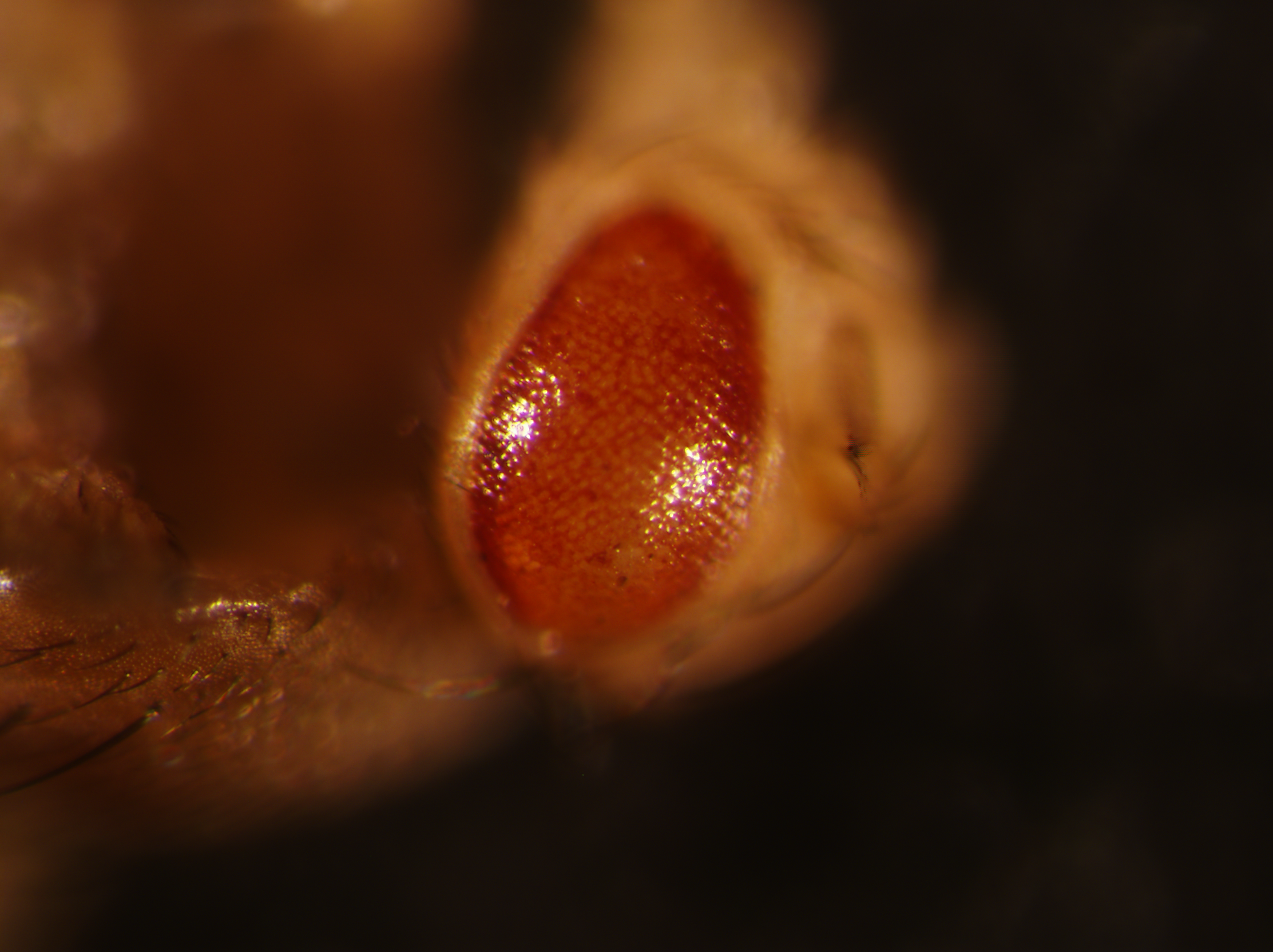

Supplement: Supplementary file 12 — Source data Fig. 6 [file 44321_2025_217_MOESM12_ESM.zip › figure 6/F6 F/SPI-112-LM.tif]

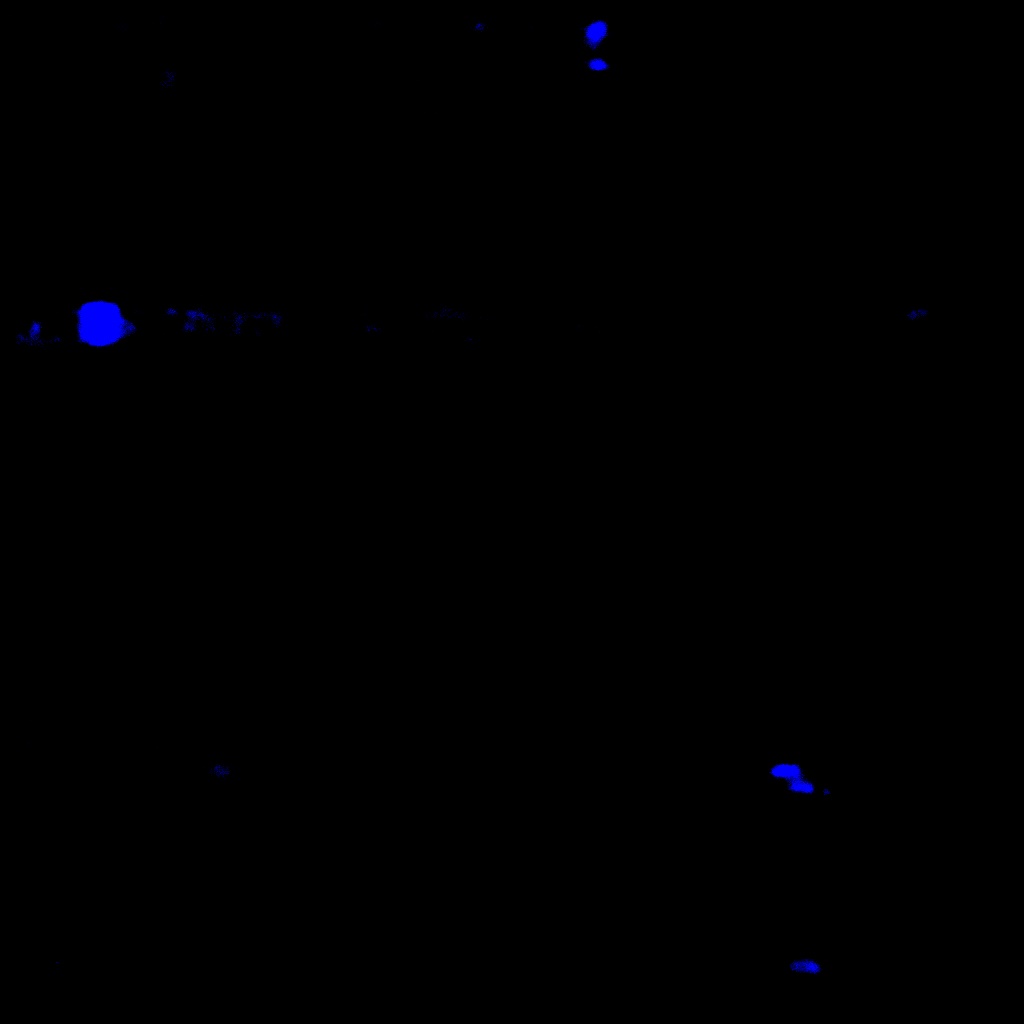

Supplement: Supplementary file 12 — Source data Fig. 6 [file 44321_2025_217_MOESM12_ESM.zip › figure 6/F6 H/Amikacin_DAPI.jpg]

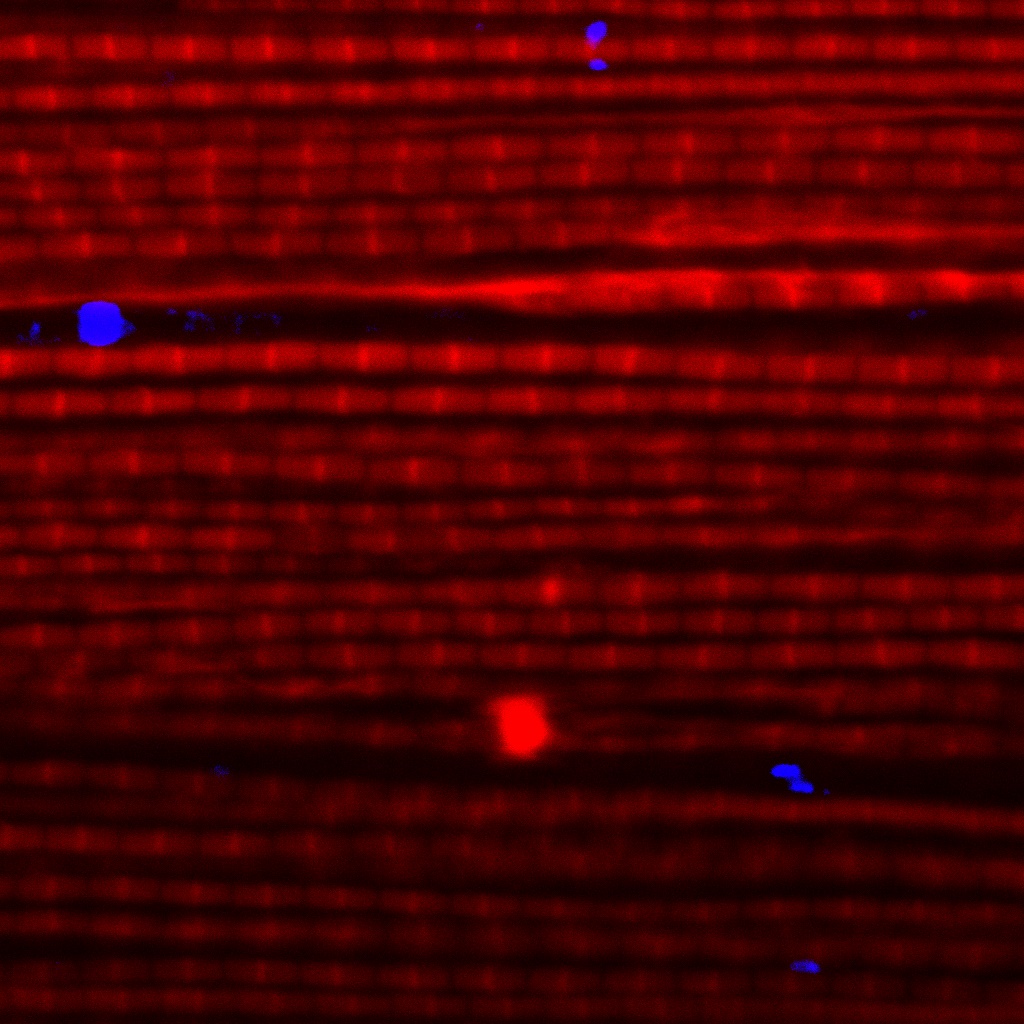

Supplement: Supplementary file 12 — Source data Fig. 6 [file 44321_2025_217_MOESM12_ESM.zip › figure 6/F6 H/Amikacin_pha+DAPI.jpg]

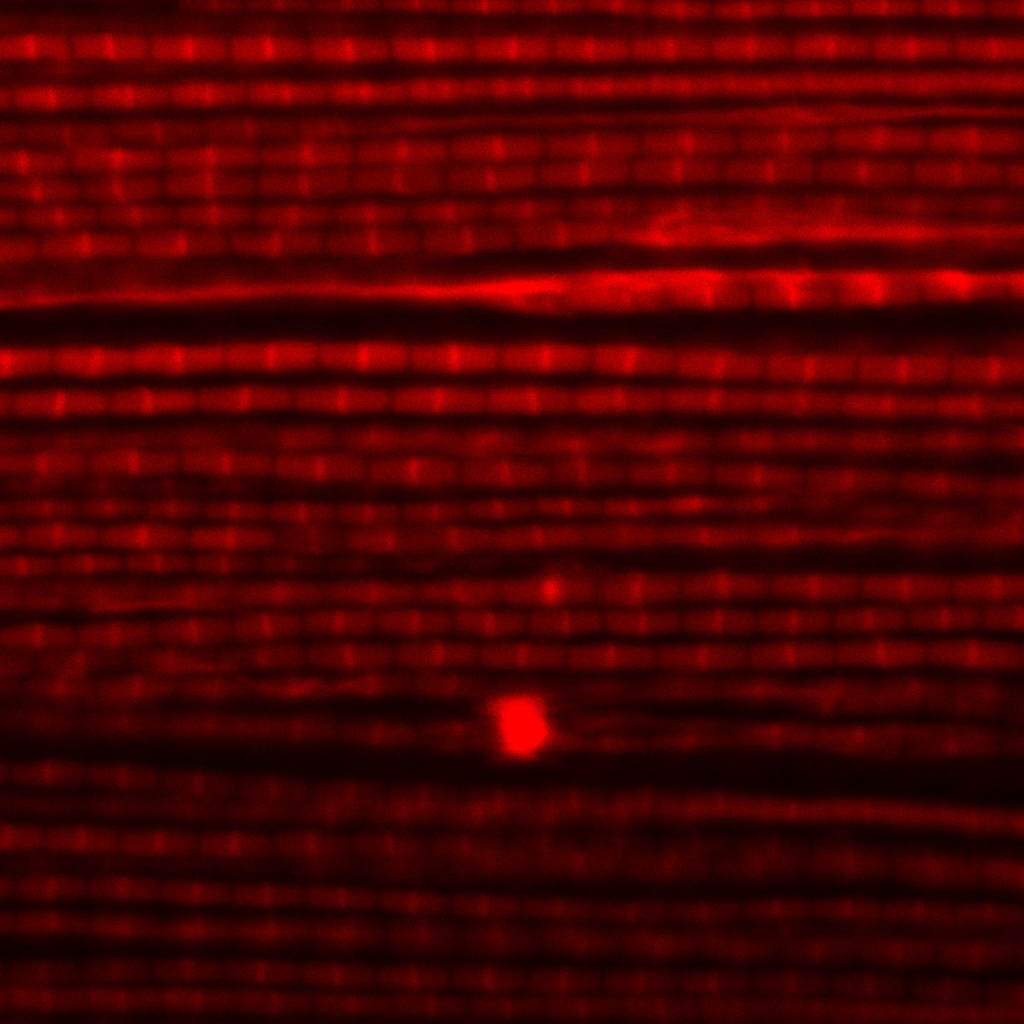

Supplement: Supplementary file 12 — Source data Fig. 6 [file 44321_2025_217_MOESM12_ESM.zip › figure 6/F6 H/Amikacin_pha.jpg]

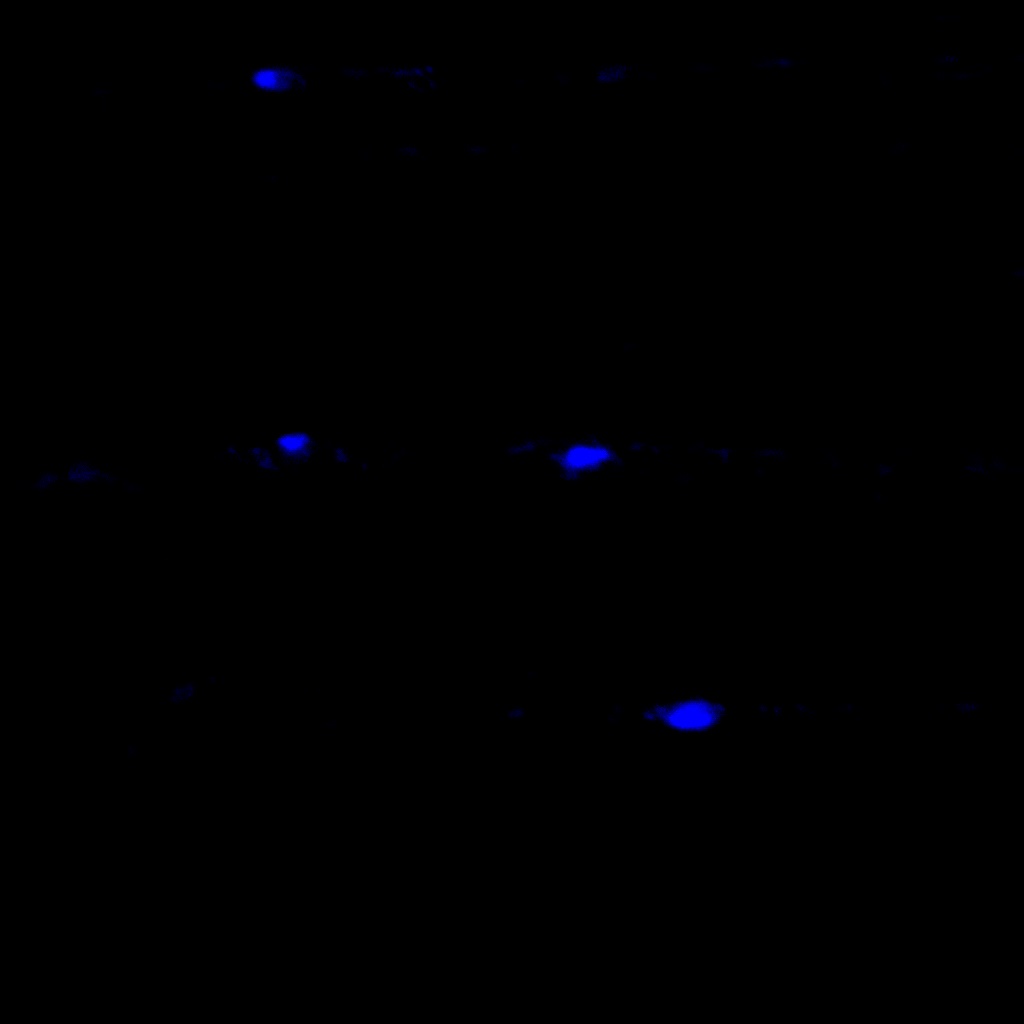

Supplement: Supplementary file 12 — Source data Fig. 6 [file 44321_2025_217_MOESM12_ESM.zip › figure 6/F6 H/CPT_DAPI.jpg]

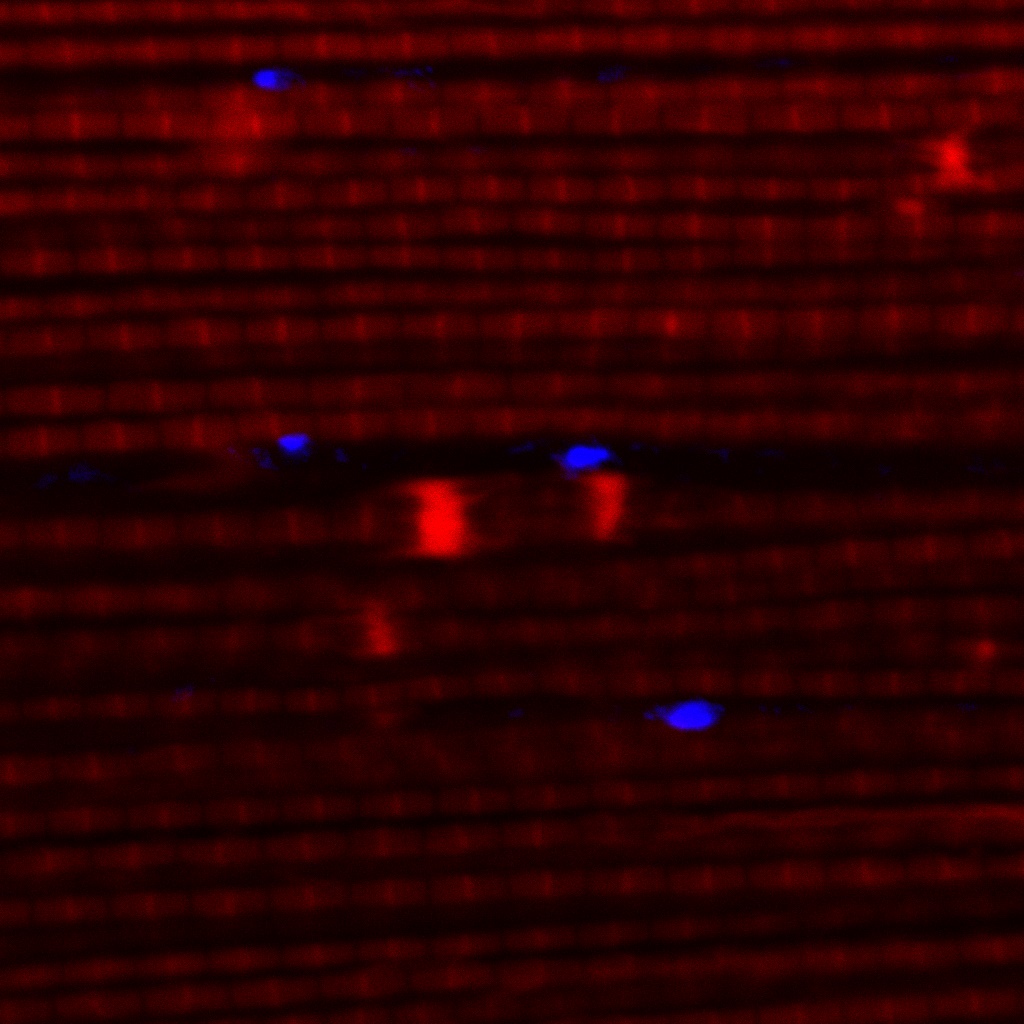

Supplement: Supplementary file 12 — Source data Fig. 6 [file 44321_2025_217_MOESM12_ESM.zip › figure 6/F6 H/CPT_pha+DAPI.jpg]

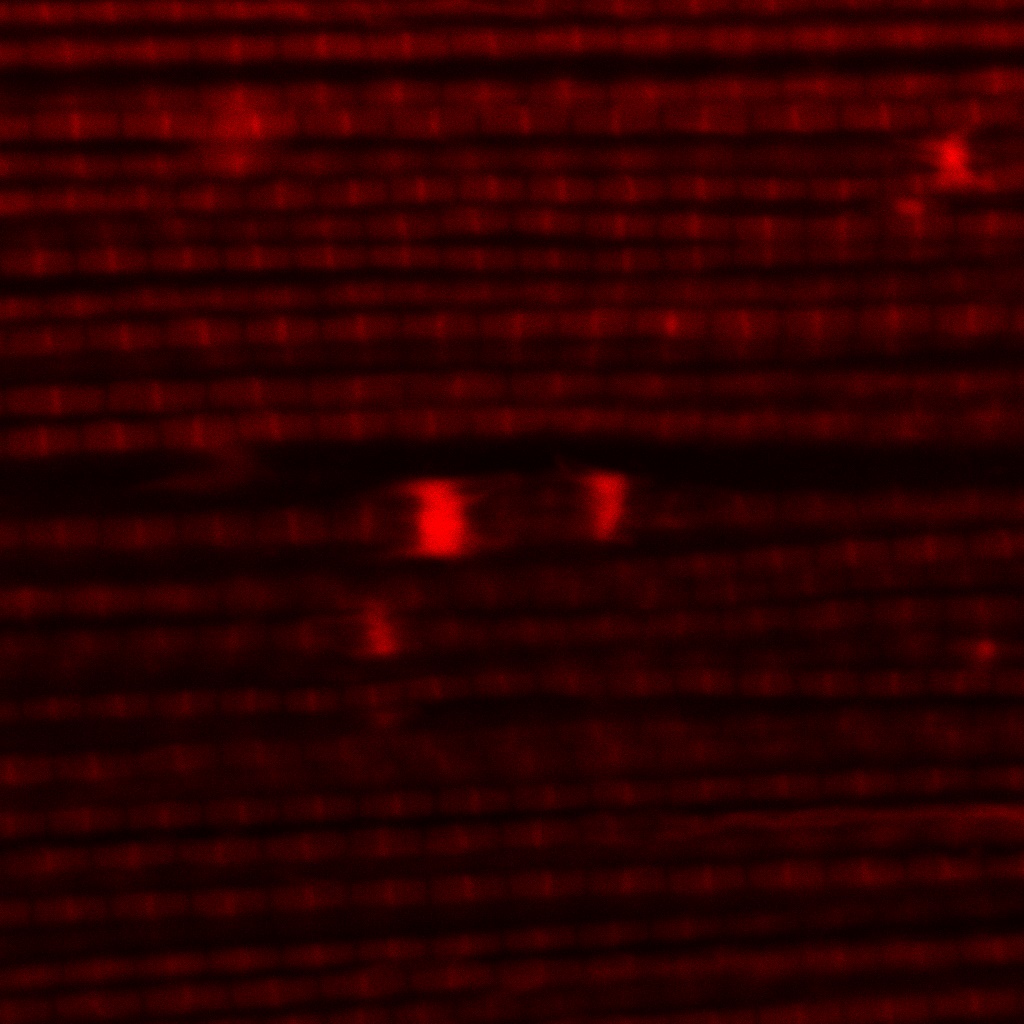

Supplement: Supplementary file 12 — Source data Fig. 6 [file 44321_2025_217_MOESM12_ESM.zip › figure 6/F6 H/CPT_pha.jpg]

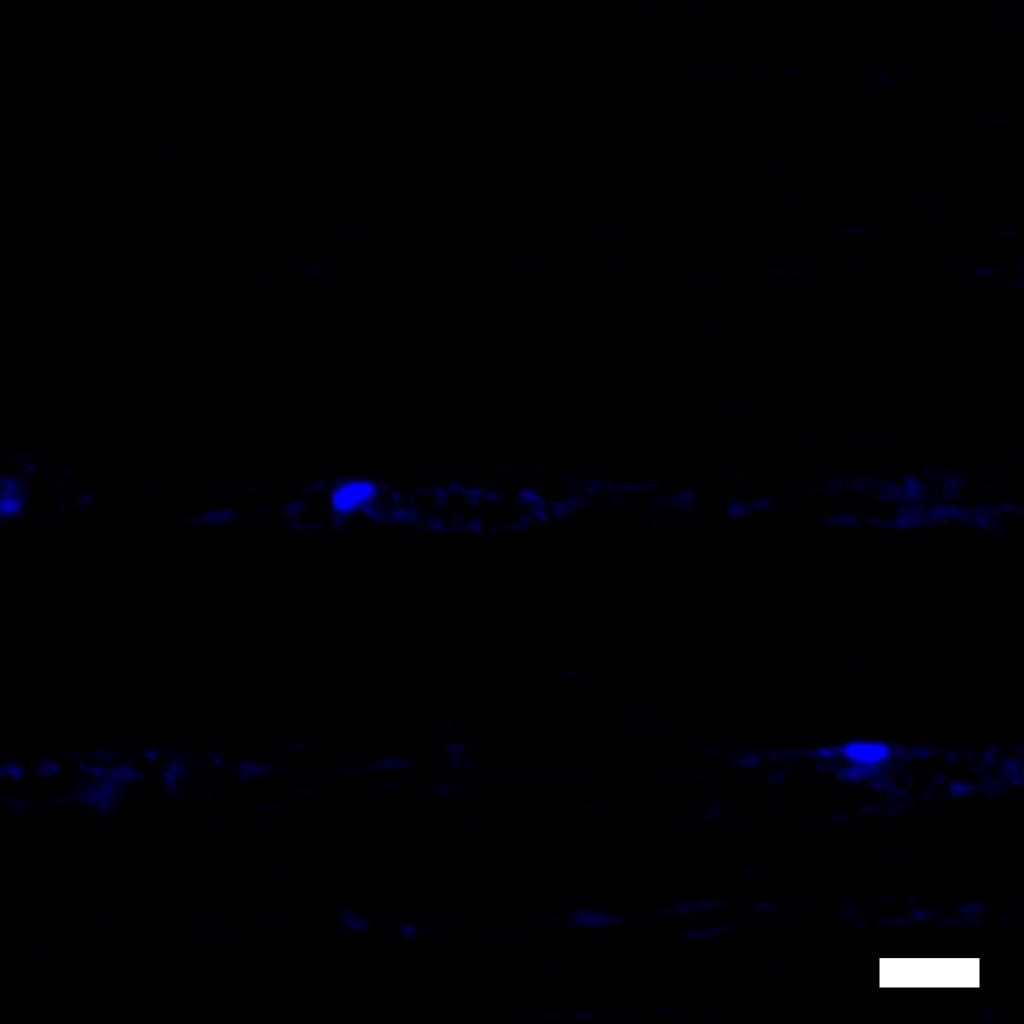

Supplement: Supplementary file 12 — Source data Fig. 6 [file 44321_2025_217_MOESM12_ESM.zip › figure 6/F6 H/DMSO_DAPI.jpg]

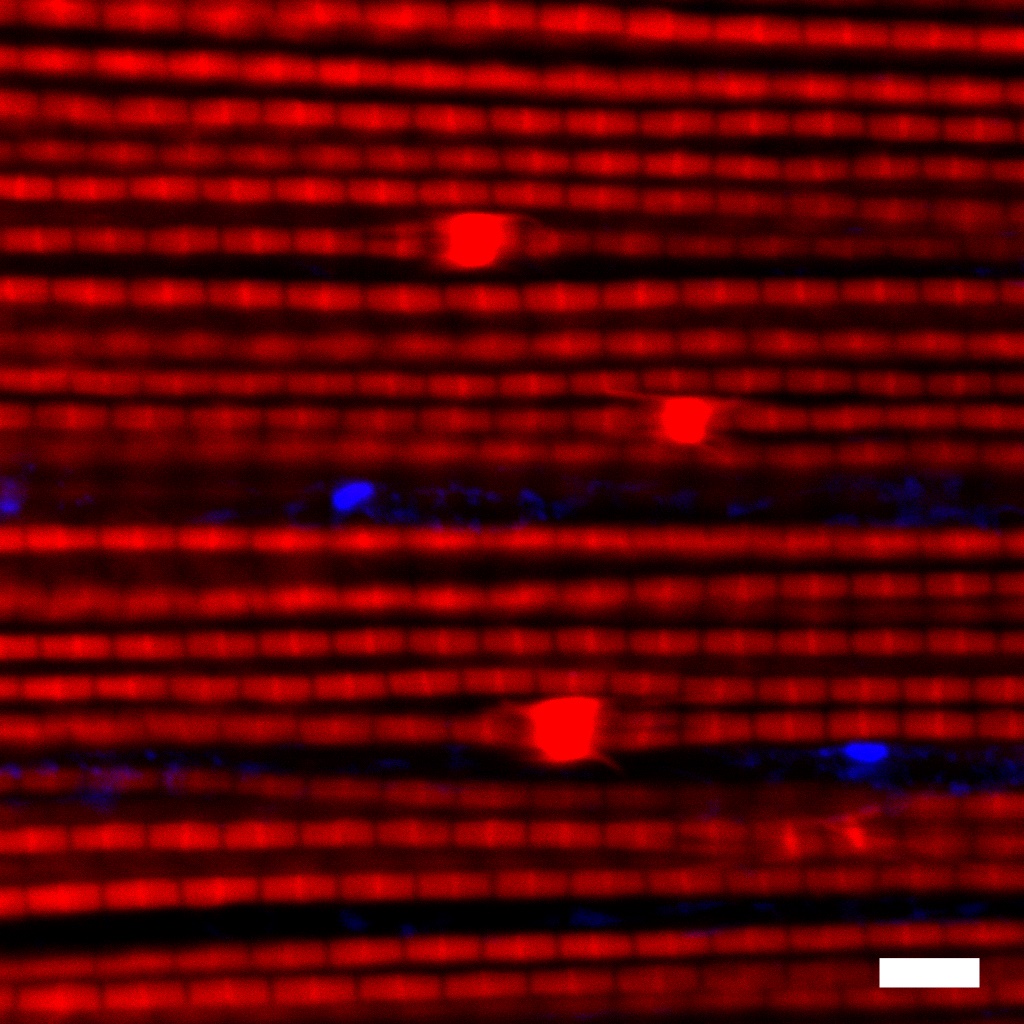

Supplement: Supplementary file 12 — Source data Fig. 6 [file 44321_2025_217_MOESM12_ESM.zip › figure 6/F6 H/DMSO_pha+DAPI.jpg]

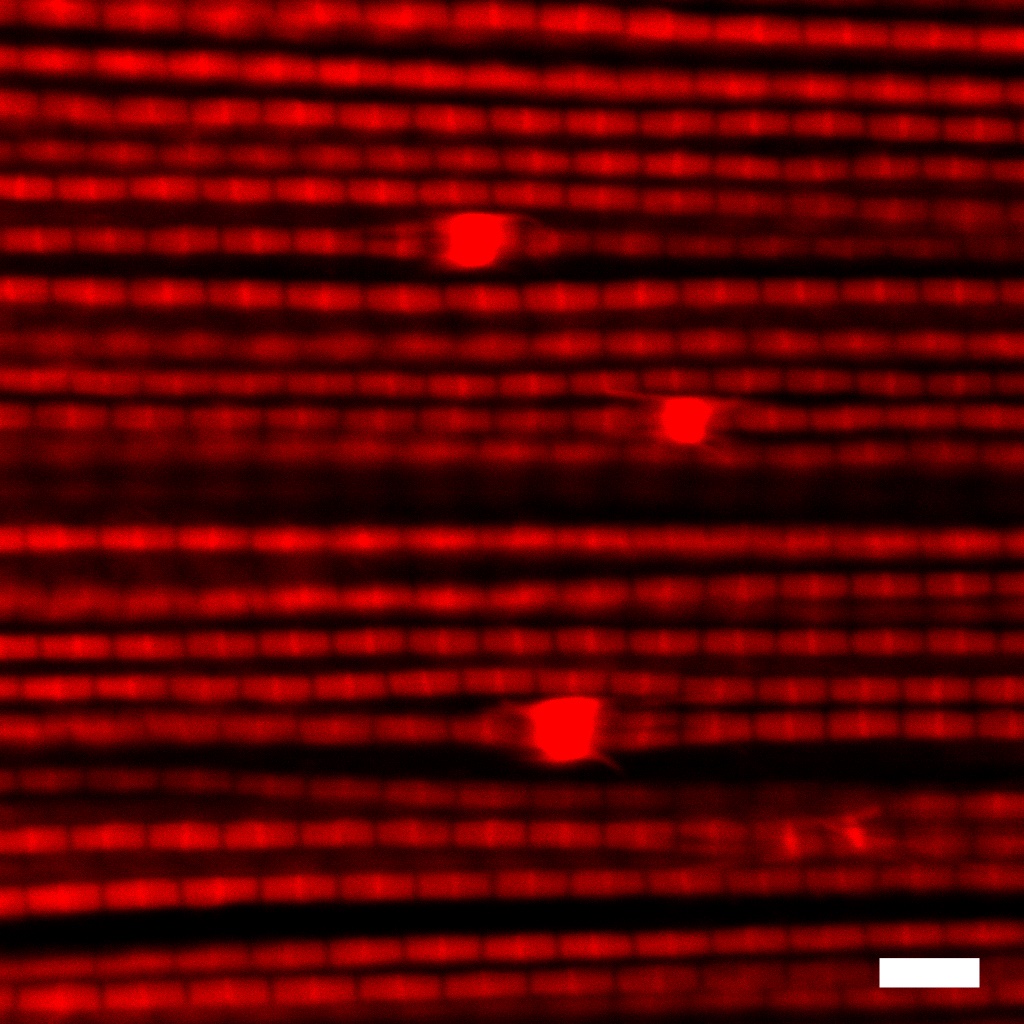

Supplement: Supplementary file 12 — Source data Fig. 6 [file 44321_2025_217_MOESM12_ESM.zip › figure 6/F6 H/DMSO_pha.jpg]

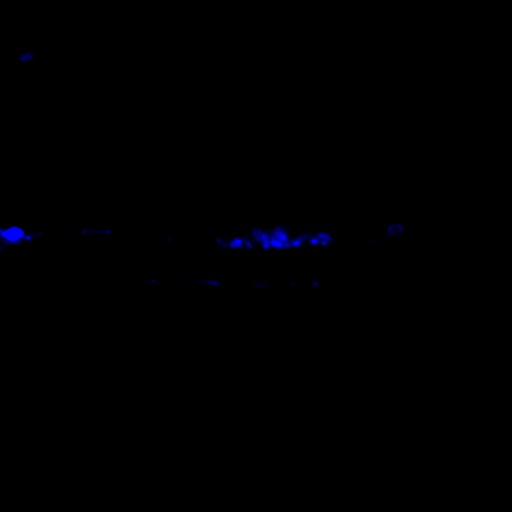

Supplement: Supplementary file 12 — Source data Fig. 6 [file 44321_2025_217_MOESM12_ESM.zip › figure 6/F6 H/Lawsone_DAPI.jpg]

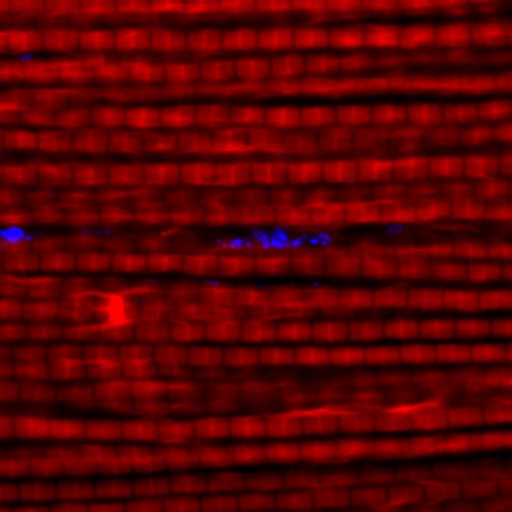

Supplement: Supplementary file 12 — Source data Fig. 6 [file 44321_2025_217_MOESM12_ESM.zip › figure 6/F6 H/Lawsone_pha+DAPI.jpg]

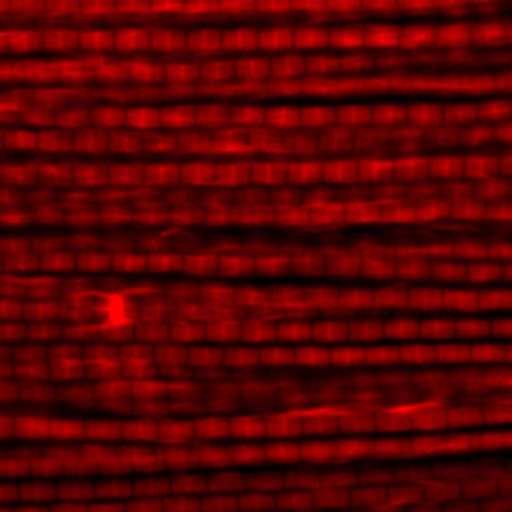

Supplement: Supplementary file 12 — Source data Fig. 6 [file 44321_2025_217_MOESM12_ESM.zip › figure 6/F6 H/Lawsone_pha.jpg]

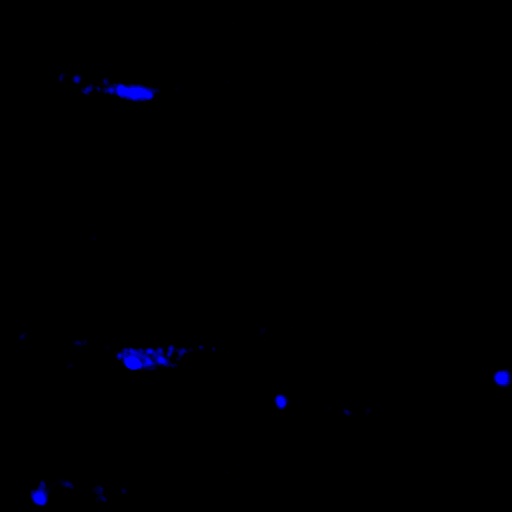

Supplement: Supplementary file 12 — Source data Fig. 6 [file 44321_2025_217_MOESM12_ESM.zip › figure 6/F6 H/SPI-112_DAPI.jpg]

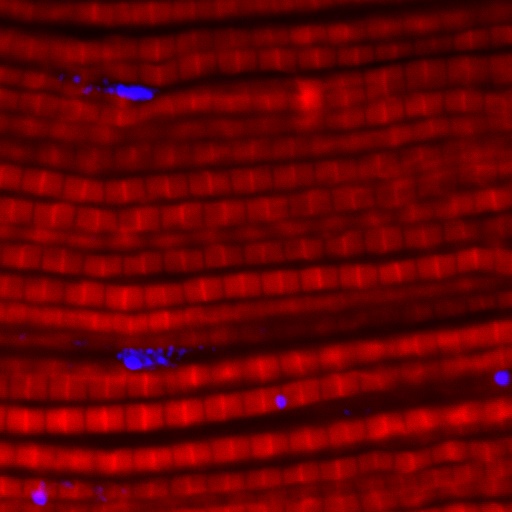

Supplement: Supplementary file 12 — Source data Fig. 6 [file 44321_2025_217_MOESM12_ESM.zip › figure 6/F6 H/SPI-112_pha+DAPI.jpg]

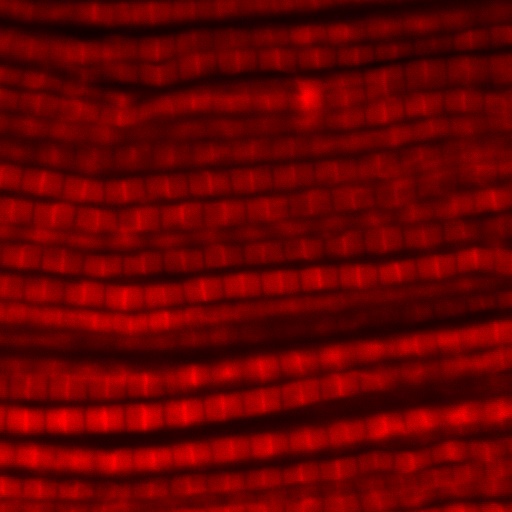

Supplement: Supplementary file 12 — Source data Fig. 6 [file 44321_2025_217_MOESM12_ESM.zip › figure 6/F6 H/SPI-112_pha.jpg]

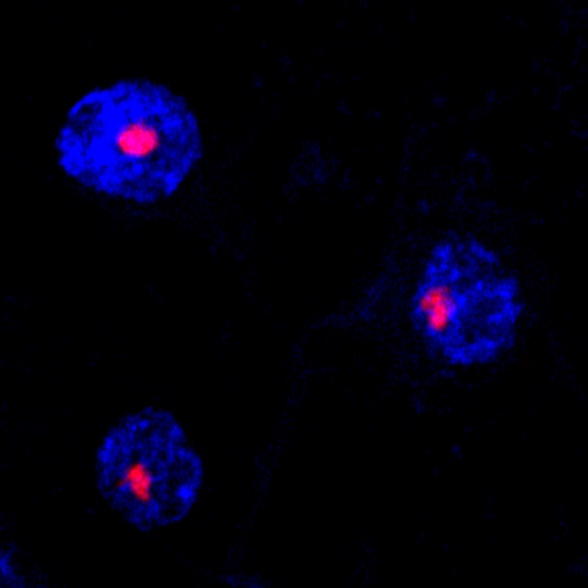

Supplement: Supplementary file 12 — Source data Fig. 6 [file 44321_2025_217_MOESM12_ESM.zip › figure 6/F6 J/Amikacin_Cy3+DAPI.jpg]

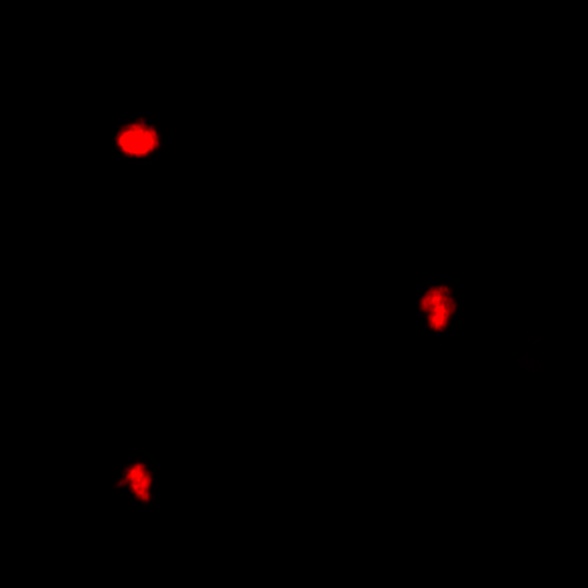

Supplement: Supplementary file 12 — Source data Fig. 6 [file 44321_2025_217_MOESM12_ESM.zip › figure 6/F6 J/Amikacin_Cy3.jpg]

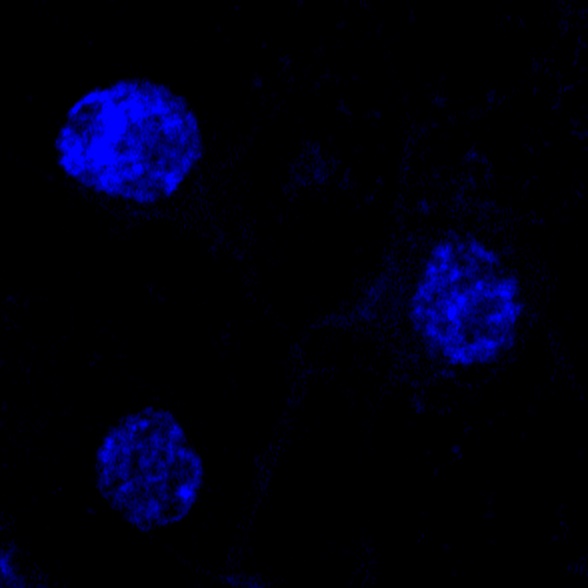

Supplement: Supplementary file 12 — Source data Fig. 6 [file 44321_2025_217_MOESM12_ESM.zip › figure 6/F6 J/Amikacin_DAPI.jpg]

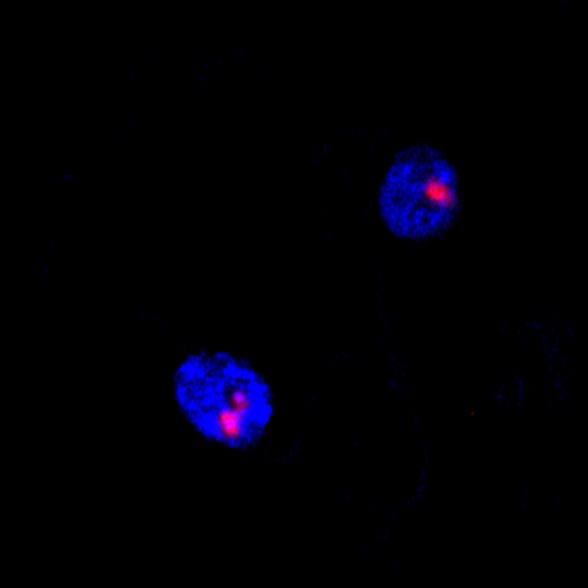

Supplement: Supplementary file 12 — Source data Fig. 6 [file 44321_2025_217_MOESM12_ESM.zip › figure 6/F6 J/CPT_Cy3+DAPI.jpg]

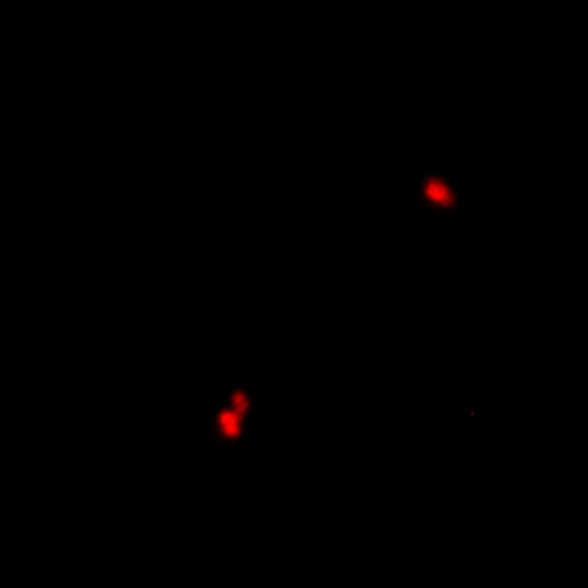

Supplement: Supplementary file 12 — Source data Fig. 6 [file 44321_2025_217_MOESM12_ESM.zip › figure 6/F6 J/CPT_Cy3.jpg]

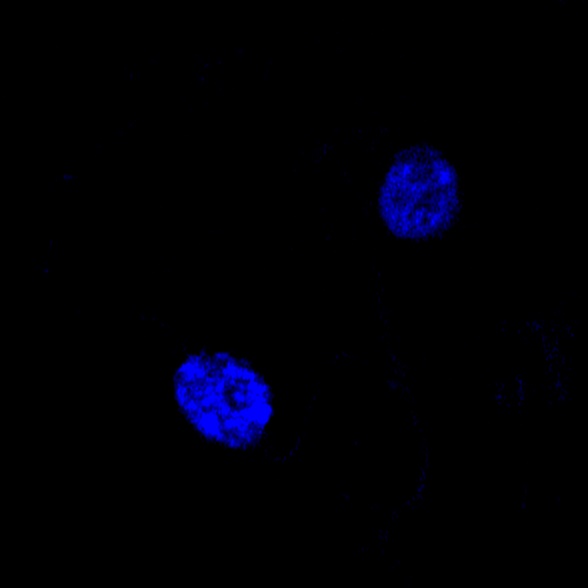

Supplement: Supplementary file 12 — Source data Fig. 6 [file 44321_2025_217_MOESM12_ESM.zip › figure 6/F6 J/CPT_DAPI.jpg]

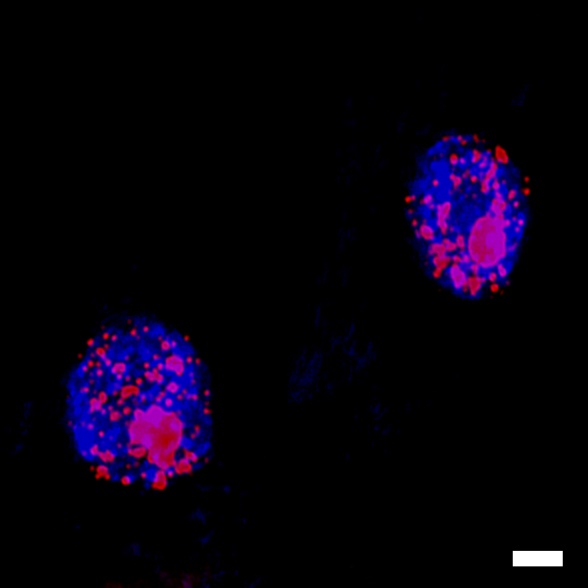

Supplement: Supplementary file 12 — Source data Fig. 6 [file 44321_2025_217_MOESM12_ESM.zip › figure 6/F6 J/DMSO_Cy3+DAPI.jpg]

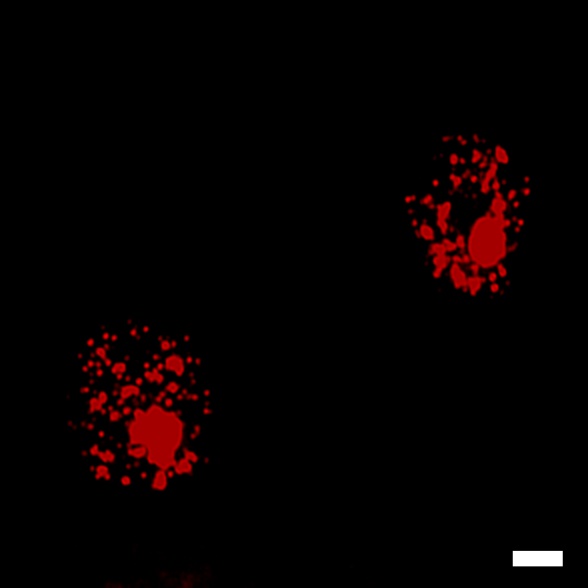

Supplement: Supplementary file 12 — Source data Fig. 6 [file 44321_2025_217_MOESM12_ESM.zip › figure 6/F6 J/DMSO_Cy3.jpg]

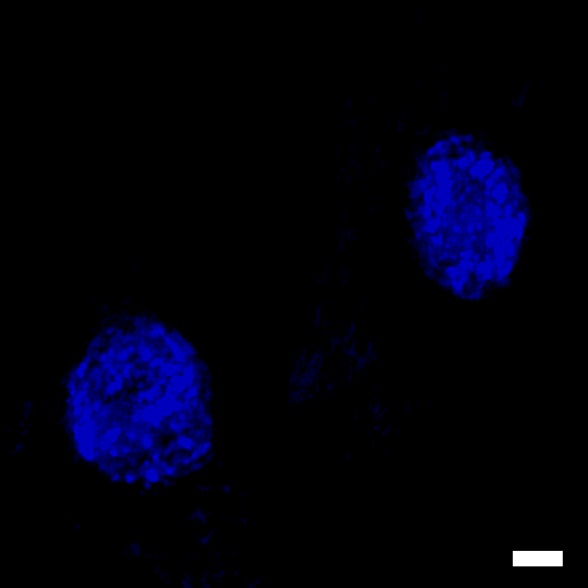

Supplement: Supplementary file 12 — Source data Fig. 6 [file 44321_2025_217_MOESM12_ESM.zip › figure 6/F6 J/DMSO_DAPI.jpg]

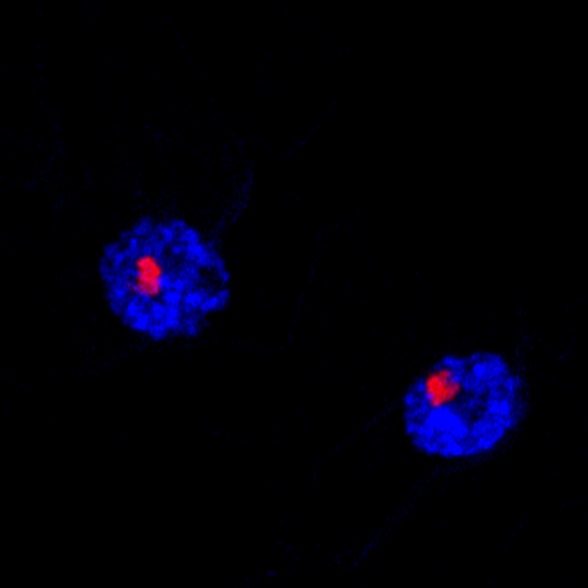

Supplement: Supplementary file 12 — Source data Fig. 6 [file 44321_2025_217_MOESM12_ESM.zip › figure 6/F6 J/Lawsone_Cy3+DAPI.jpg]

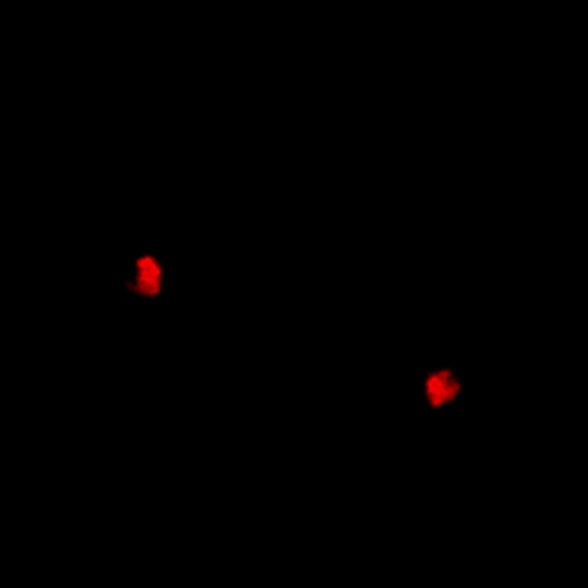

Supplement: Supplementary file 12 — Source data Fig. 6 [file 44321_2025_217_MOESM12_ESM.zip › figure 6/F6 J/Lawsone_Cy3.jpg]

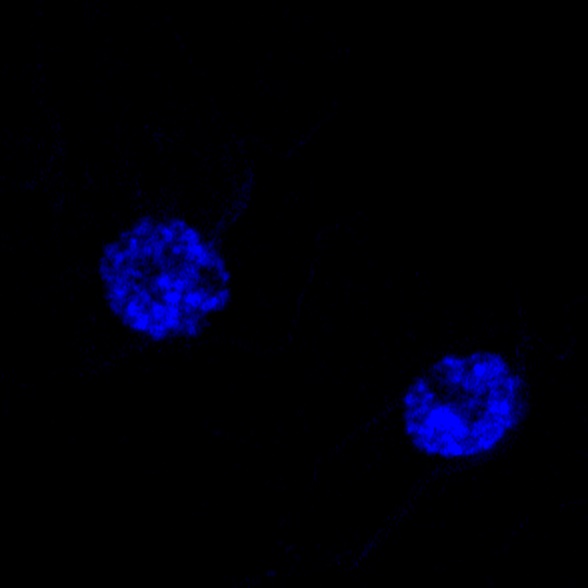

Supplement: Supplementary file 12 — Source data Fig. 6 [file 44321_2025_217_MOESM12_ESM.zip › figure 6/F6 J/Lawsone_DAPI.jpg]

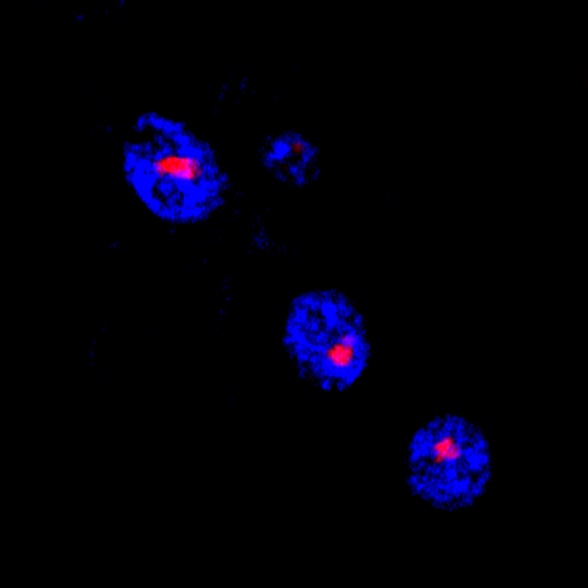

Supplement: Supplementary file 12 — Source data Fig. 6 [file 44321_2025_217_MOESM12_ESM.zip › figure 6/F6 J/SPI-112_Cy3+DAPI.jpg]

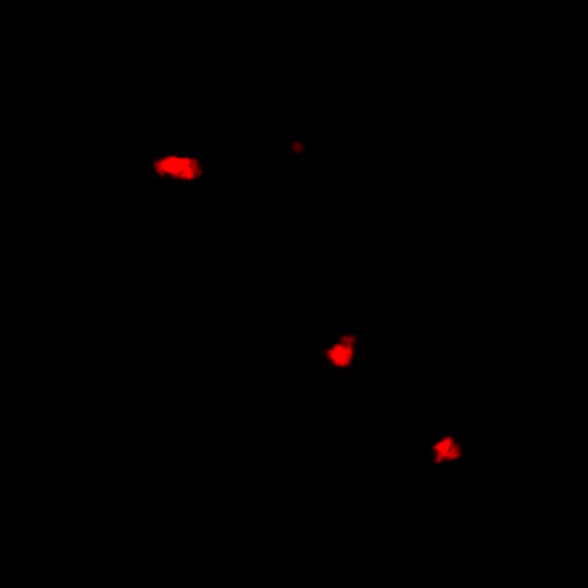

Supplement: Supplementary file 12 — Source data Fig. 6 [file 44321_2025_217_MOESM12_ESM.zip › figure 6/F6 J/SPI-112_Cy3.jpg]

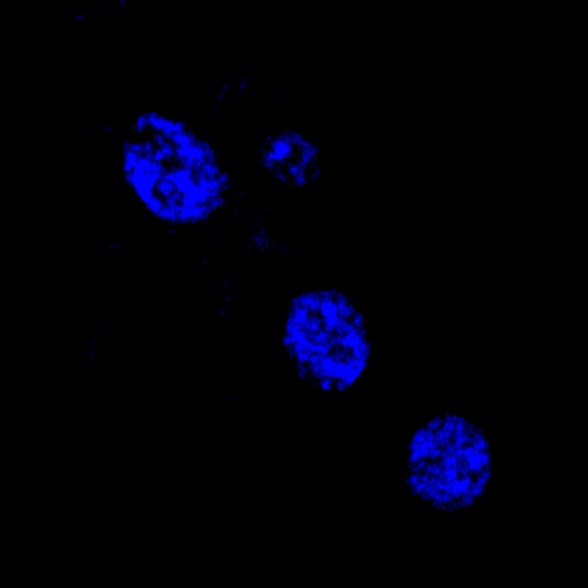

Supplement: Supplementary file 12 — Source data Fig. 6 [file 44321_2025_217_MOESM12_ESM.zip › figure 6/F6 J/SPI-112_DAPI.jpg]

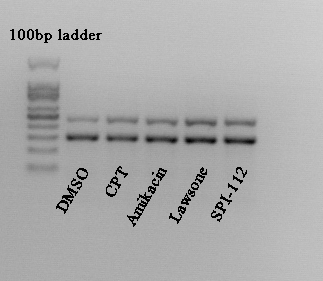

Supplement: Supplementary file 12 — Source data Fig. 6 [file 44321_2025_217_MOESM12_ESM.zip › figure 6/F6 L/F6 L upper.tif]

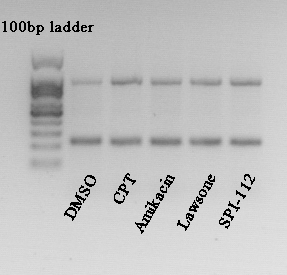

Supplement: Supplementary file 12 — Source data Fig. 6 [file 44321_2025_217_MOESM12_ESM.zip › figure 6/F6 M/F6 M upper.tif]
