# Supplementary material for: Modulating CCTG repeat expansion toxicity in DM2 Drosophila model through TDP1 inhibition
Source: EMBO Mol Med. 2025 Mar 25;17(5):967–92. doi: 10.1038/s44321-025-00217-3 (PMC12081759; doi:10.1038/s44321-025-00217-3)
Supplement: Supplementary file 13 — Expanded View Figures [file 44321_2025_217_MOESM13_ESM.pdf]

Expanded View Figures

Figure EV1. TDP1/gkt mutation alleviated neurodegeneration and muscle atrophy in DM2 flies.

(**A**) Quantification of the average counts per day in flies across different genotypes from 15-days-old to the 25-days-old. Five biological replicates. Data are mean ± SD. Two-tailed, unpaired *t* test. (**B**, **C**) The average counts of per 30 min in flies across different genotypes from 15-days-old to the 25-days-old. Fifteen animals per sample. (C) Quantification from five biological replicates. Data are mean ± SD. Two-tailed, unpaired t-test. ****$P < 0.0001$ $(CCTG)_{720}$ versus $(CCTG)_{720}$ + *gkt mut*. (**D**, **E**) Representative images of paraffin-embedded adult thoraces showing IFMs of flies at 1, 7, 15-days-old. Scale bars 100 μm. (**E**) Quantification at least ten independent thoraces of each sample/experiment, 3 experiments. Data are mean ± SD. Two-tailed, unpaired *t* test. **$P = 0.084$ 1-day-old versus 7-days-old $(CCTG)_{720}$, ***$P = 0.00042$ 7-days-old versus 15-days-old $(CCTG)_{720}$. (**F–H**) Representative images of paraffin-embedded adult thoraces showing IFMs of flies at 15-days-old. Black arrows, holes and fragmentations. Scale bars 100 μm. (**G**, **H**) Quantification at least ten independent thoraces of each sample/experiment, three experiments. Data are mean ± SD. Two-tailed, unpaired *t* test. ****$P < 0.0001$ $(CCTG)_{16}$ versus $(CCTG)_{720}$, ****$P < 0.0001$ $(CCTG)_{720}$ versus $(CCTG)_{720}$ + *gkt mut*. (**I**) Quantification of climbing index across different genotypes at 15-days-old from five biological replicates. Data are mean ± SD. Two-tailed, unpaired *t* test. ****$P < 0.0001$ $(CCTG)_{16}$ versus $(CCTG)_{720}$, ****$P < 0.0001$ $(CCTG)_{720}$ versus $(CCTG)_{720}$ + *gkt RI 1*, ****$P < 0.0001$ $(CCTG)_{720}$ versus $(CCTG)_{720}$ + *gkt RI 2*. (**J**, **K**) Representative images of the compound eyes across different genotypes at 15-days-old. Top, LM images. Middle and Bottom, SEM images. Scale bars 20 μm. (**K**) Quantification at least sixty independent eyes of each genotype/experiment, three experiments. Data are mean ± SD. Two-tailed, unpaired *t* test. ****$P < 0.0001$.

 © The Author(s)

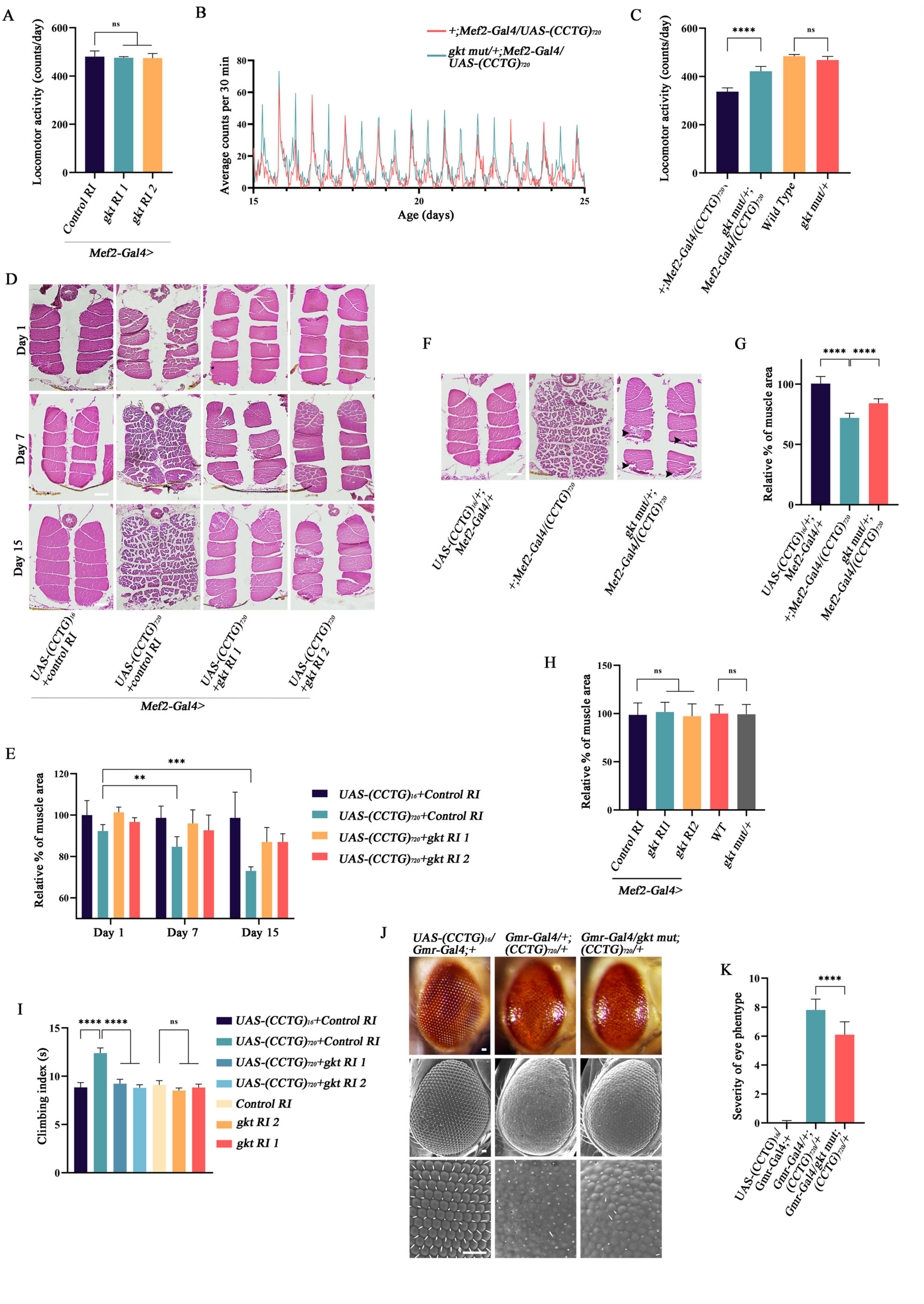
A
Locomotor activity (counts/day)
ns
Control RI
gkt RI 1
gkt RI 2
Mef2-Gal4>
B
+;Mef2-Gal4/UAS-(CCTG)720
gkt mut/+;Mef2-Gal4/UAS-(CCTG)720
Average counts per 30 min
Age (days)
C
Locomotor activity (counts/day)
****
ns
+;Mef2-Gal4/(CCTG)720
gkt mut/+;Mef2-Gal4/(CCTG)720
Wild Type
gkt mut/+
D
Day 1
Day 7
Day 15
UAS-(CCTG)16+control RI
UAS-(CCTG)720+control RI
UAS-(CCTG)720+gkt RI 1
UAS-(CCTG)720+gkt RI 2
Mef2-Gal4>
E
Relative % of muscle area
**
***
Day 1
Day 7
Day 15
UAS-(CCTG)16+Control RI
UAS-(CCTG)720+Control RI
UAS-(CCTG)720+gkt RI 1
UAS-(CCTG)720+gkt RI 2
F
UAS-(CCTG)16/+; Mef2-Gal4/+
+;Mef2-Gal4/(CCTG)720
gkt mut/+; Mef2-Gal4/(CCTG)720
G
Relative % of muscle area
**** ****
H
Relative % of muscle area
ns
Control RI
gkt RI1
gkt RI2
WT
gkt mut/+
Mef2-Gal4>
I
Climbing index (s)
**** ****
ns
UAS-(CCTG)16+Control RI
UAS-(CCTG)720+Control RI
UAS-(CCTG)720+gkt RI 1
UAS-(CCTG)720+gkt RI 2
Control RI
gkt RI 2
gkt RI 1
J
UAS-(CCTG)16/Gmr-Gal4;+
Gmr-Gal4/+;(CCTG)720/+
Gmr-Gal4/gkt mut;(CCTG)720/+
K
Severity of eye phentype
****
UAS-(CCTG)16/Gmr-Gal4;+
Gmr-Gal4/+;(CCTG)720/+
Gmr-Gal4/gkt mut;(CCTG)720/+

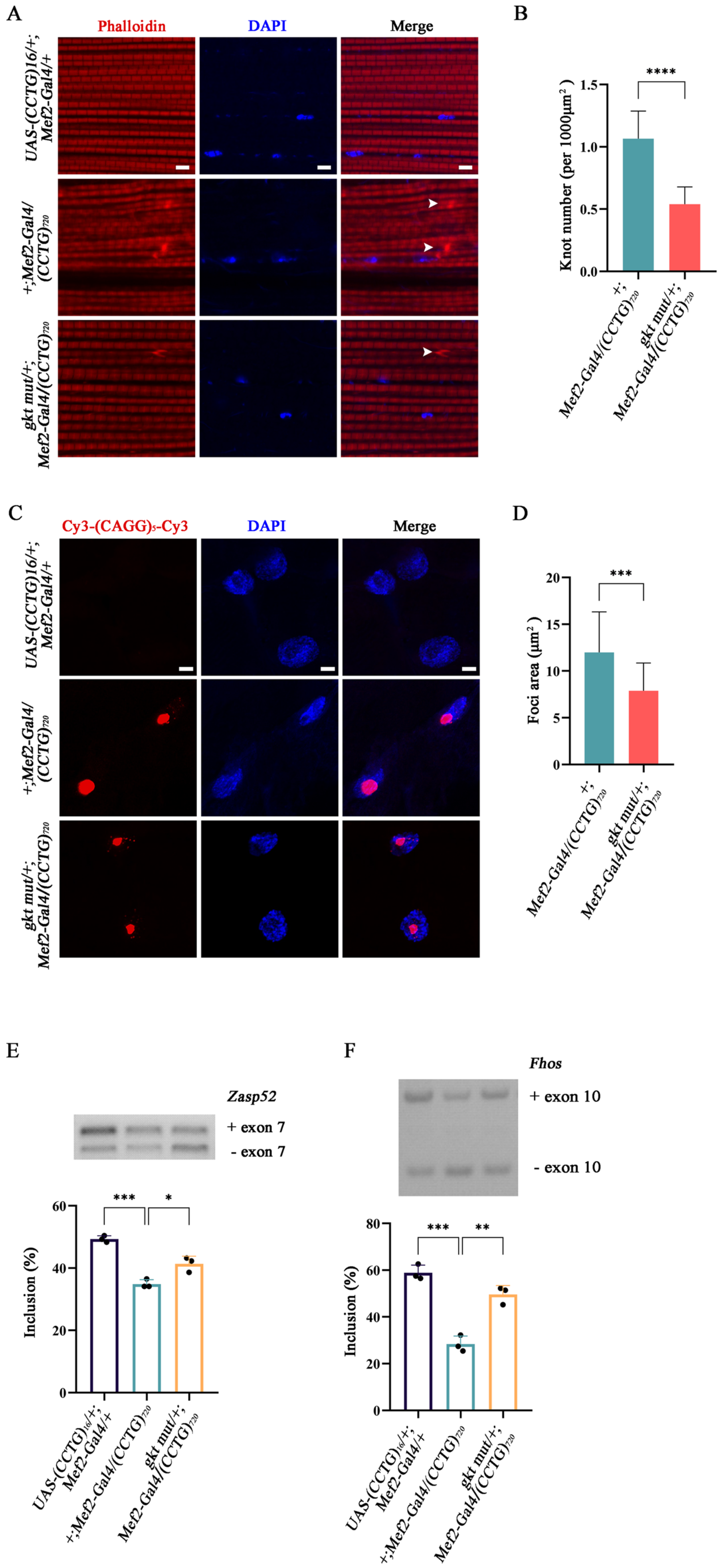
A
Phalloidin
DAPI
Merge
UAS-(CCTG)16/+; Mef2-Gal4/+
+;Mef2-Gal4/(CCTG)720
gkt mut/+; Mef2-Gal4/(CCTG)720
B
****
Knot number (per 1000μm²)
1.5
1.0
0.5
0.0
+; Mef2-Gal4/(CCTG)720
gkt mut/+; Mef2-Gal4/(CCTG)720
C
Cy3-(CAGG)5-Cy3
DAPI
Merge
UAS-(CCTG)16/+; Mef2-Gal4/+
+;Mef2-Gal4/(CCTG)720
gkt mut/+; Mef2-Gal4/(CCTG)720
D
***
Foci area (μm²)
20
15
10
5
0
+; Mef2-Gal4/(CCTG)720
gkt mut/+; Mef2-Gal4/(CCTG)720
E
Zasp52
+ exon 7
- exon 7
***
*
Inclusion (%)
60
40
20
0
UAS-(CCTG)16/+; Mef2-Gal4/+
+;Mef2-Gal4/(CCTG)720
gkt mut/+; Mef2-Gal4/(CCTG)720
F
Fhos
+ exon 10
- exon 10
***
**
Inclusion (%)
80
60
40
20
0
UAS-(CCTG)16/+; Mef2-Gal4/+
+;Mef2-Gal4/(CCTG)720
gkt mut/+; Mef2-Gal4/(CCTG)720

© The Author(s)

Figure EV2. TDP1/gkt mutation rescues molecular pathology of DM2.

(**A**, **B**) Representative IF images of thoracic muscle fibers across different genotypes at 15-days-old. White arrows, action blobs. Scale bars 5 µm. (**B**) Quantification at least ten animals of each genotype. Data are mean ± SD. Two-tailed, unpaired *t* test. ****$P < 0.0001$. (**C**, **D**) Representative Confocal images of CCUG-repeat foci with $(CAGG)_5$ probe across different genotypes at body-wall muscles of third instar larvae. Scale bars 5 µm. (**D**) Quantification at least ten animals of each genotype/experiment, three experiments. Data are mean ± SD. Two-tailed, unpaired *t* test. ***$P = 0.0003$. (**E**) Representative gel image of endogenous *Zasp52* exon 7 with RT-PCR in flies at 15-days-old. Tissue from ten animals per sample/experiments, 5 experiments. Lower panel, quantification of *Zasp52* exon 7 inclusion from three biological replicates. Data are mean ± SD. Two-tailed, unpaired *t* test. *$P = 0.0162$, ***$P = 0.0001$. (**F**) Representative gel image of endogenous *Fhos* exon 10 with RT-PCR in flies at 15-days-old. Tissue from ten animals per sample/experiment, 5 experiments. Lower panel, quantification of *Fhos* exon 10 inclusion from three biological replicates. Data are mean ± SD. Two-tailed, unpaired *t* test. **$P = 0.021$,***$P = 0.0004$.

© The Author(s)

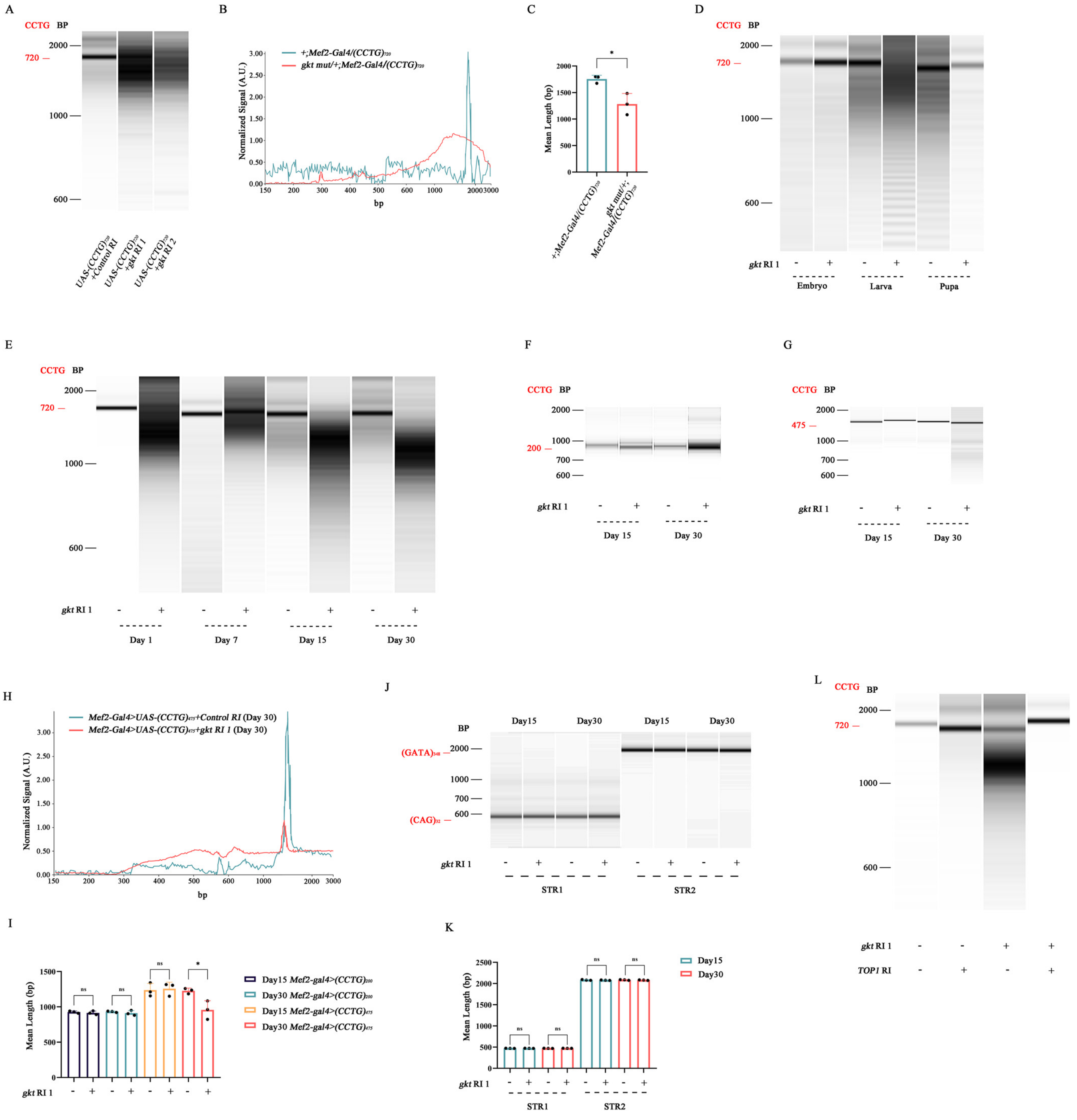

Figure EV3. TDP1/gkt loss of function induce large CCTG repeats contraction but not affect stability of endogenous STR loci.

(**A**) Representative gel image of PCR across different genotypes at 15-days-old from bioanalyzer. Tissue from three animals per sample. (**B**, **C**) Bioanalyzer quantitation of CCTG repeat lengths across different genotypes at 15-days-old. Tissue from three animals per sample. (**B**) Quantification of mean length of CCTG repeats from three biological replicates. Data are mean ± SD. Two-tailed, unpaired *t* test. $^*P = 0.0182$. (**D**) Representative gel image of PCR across different genotypes at embryo, larva, and pupa. Embryo from ten animals per sample. Larva and pupa from five animals per sample. (**E**) Representative gel image of PCR across *Mef2-Gal4 > UAS-(CCTG)*$_{720}$ +*Control RI* and *Mef2-Gal4 > UAS-(CCTG)*$_{720}$+*gkt RI 1* at 1, 7, 15, 30-days-old. Tissue from three animals per sample. (**F**) Representative gel image of PCR across *Mef2-Gal4 > UAS-(CCTG)*$_{200}$ +*Control RI* and *Mef2-Gal4 > UAS-(CCTG)*$_{200}$+*gkt RI 1* at 15-days-old and 30-days-old. Tissue from three animals per sample. (**G**) Representative gel image of PCR across *Mef2-Gal4 > UAS-(CCTG)*$_{475}$ +*Control RI* and *Mef2-Gal4 > UAS-(CCTG)*$_{475}$+*gkt RI 1* at 15-days-old and 30-days-old. Tissue from three animals per sample. (**H**) Bioanalyzer quantitation of CCTG repeat lengths across *Mef2-Gal4 > UAS-(CCTG)*$_{475}$ +*Control RI* and *Mef2-Gal4 > UAS-(CCTG)*$_{475}$+*gkt RI 1* at 30-days-old. Tissue from three animals per sample. (**I**) Quantification of mean length of CCTG repeats from three biological replicates. Data are mean ± SD. Two-tailed, unpaired *t* test. $^*P = 0.0263$. (**J**, **K**) Representative gel image of STR1 and STR2 PCR across *Mef2-Gal4 > UAS-(CCTG)*$_{720}$ +*Control RI* and *Mef2-Gal4 > UAS-(CCTG)*$_{720}$+*gkt RI 1* at 15-days-old and 30-days-old. Tissue from three animals per sample. (**K**) Quantification of mean length of GATA and CAG repeats from three biological replicates. Two-tailed, unpaired *t* test. (**L**) Representative gel image of across different genotype at 15-days-old. Tissue from three animals per sample.

© The Author(s)

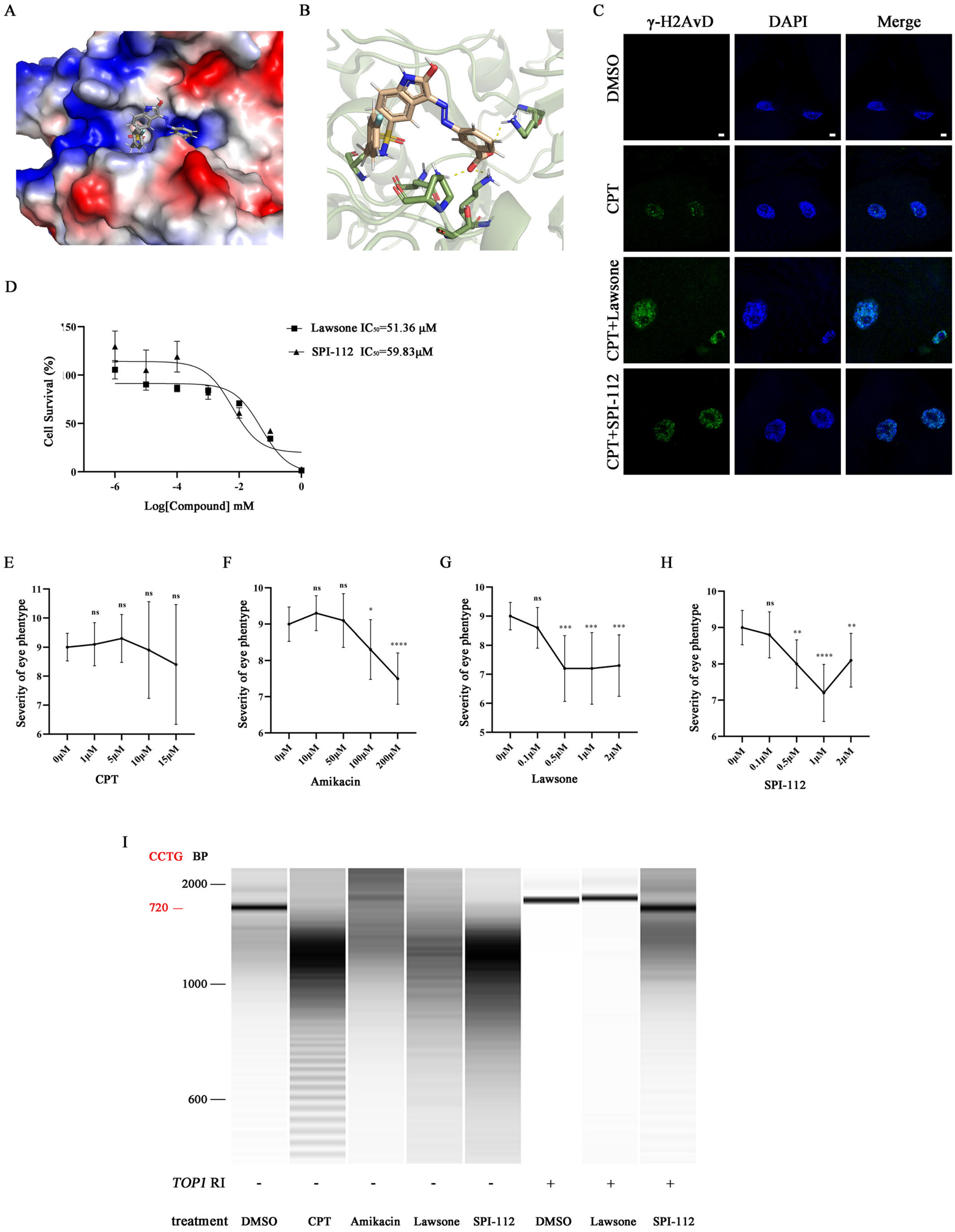
A
B
C
γ-H2AvD
DAPI
Merge
DMSO
CPT
CPT+Lawsone
CPT+SPI-112
D
Lawsone IC50=51.36 μM
SPI-112 IC50=59.83μM
Cell Survival (%)
Log[Compound] mM
E
F
G
H
Severity of eye phentype
CPT
Amikacin
Lawsone
SPI-112
0μM
1μM
5μM
10μM
15μM
50μM
100μM
200μM
0.1μM
0.5μM
2μM
ns
I
CCTG
BP
2000
720
1000
600
TOP1 RI
treatment
DMSO
CPT
Amikacin
Lawsone
SPI-112

Figure EV4. Small molecules predicted to bind TDP1 and cytotoxicity.

(**A**) Surface electrostatic potentials of predicted crystal structure of human TDP1/SPI-112 complex. Red depicts a positive partial charge on the surface, blue depicts negative partial charge and gray shows neutral/lipophilic areas. (**B**) Docking model of Lawsone bind to TDP1 and SPI-112. Dotted yellow line: hydrogen bond, Dotted green line: Pi bond. Blue: N atom, Red: O atom, White: H atom, Green: protein skeleton, Gold: compound skeleton. (**C**) Representative IF images of body-wall muscles of third instar larvae from WT flies. Scale bars 5 μm. (**D**) Dose-survival analysis of Lawsone and SPI-112. The HEK293 cells were treated with purified TDP1 enzyme 0, 0.001, 0.01, 0.1, 1, 10, 100, 1000 μM for 72 h. Data are mean ± SD from three biological replicates. IC_{50} values were calculated using log(inhibitor) compared with normalized survival rate (variable slope), using the program Prism, and are given in graphs. (**E**) Quantitative analysis of compound eye phenotypic severity of *Gmr-Gal4 > UAS-(CCTG)*$_{720}$ after treatment with 0, 1, 5, 10, 15 μM CPT, at least twenty independent eyes of each sample/experiment, 3 experiments. Flies treated with 20 μM CPT were lethal. Data are mean ± SD. Two-tailed, unpaired *t* test. (**F**) Quantitative analysis of compound eye phenotypic severity of *Gmr-Gal4 > UAS-(CCTG)*$_{720}$ after treatment with 0, 10, 50, 100, 200 μM Amikacin, at least twenty independent eyes of each sample/experiment, 3 experiments. Flies treated with 400 μM Amikacin were lethal. Data are mean ± SD. Two-tailed, unpaired *t* test. *$P = 0.0314$, ****$P < 0.0001$. (**G**) Quantitative analysis of compound eye phenotypic severity of *Gmr-Gal4 > UAS-(CCTG)*$_{720}$ after treatment with 0, 0.1, 0.5, 1, 2 μM Lawsone, at least twenty independent eyes of each sample/experiment, 3 experiments. Data are mean ± SD. Two-tailed, unpaired *t* test. ***$P = 0.0002$ 0 μM versus 0.5 μM, ***$P = 0.0004$ 0 μM versus 1 μM, ***$P = 0.0002$ 0 μM versus 2 μM. (**H**) Quantitative analysis of compound eye phenotypic severity of *Gmr-Gal4 > UAS-(CCTG)*$_{720}$ after treatment with 0, 0.1, 0.5, 1, 2 μM SPI-112, at least twenty independent eyes of each sample/experiment, 3 experiments. Data are mean ± SD. Two-tailed, unpaired *t* test. **$P = 0.0011$ 0 μM versus 0.5 μM, ****$P < 0.0001$ 0 μM versus 1 μM, **$P = 0.0044$ 0 μM versus 2 μM. (**I**) Representative gel image of across different genotype with treatment of different compounds at 15-days-old. Tissue from three animals per sample.

 © The Author(s)

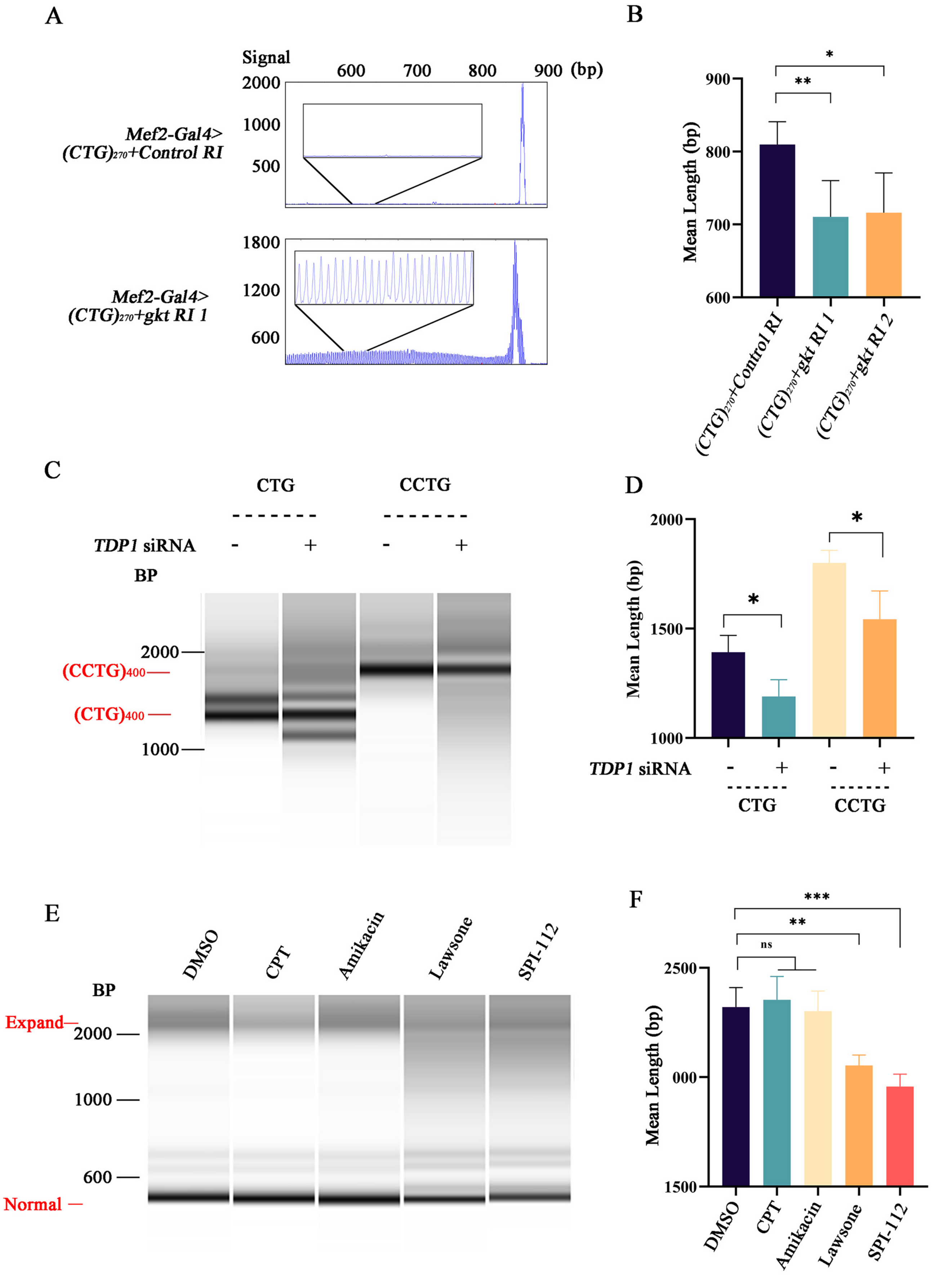
A
Signal
600 700 800 900 (bp)
2000
1000
500
Mef2-Gal4>
(CTG)270+Control RI
1800
1200
600
Mef2-Gal4>
(CTG)270+gkt RI 1
B
Mean Length (bp)
900
800
700
600
*
**
(CTG)270+Control RI
(CTG)270+gkt RI 1
(CTG)270+gkt RI 2
C
CTG
CCTG
TDP1 siRNA
- + - +
BP
2000
(CCTG)400
(CTG)400
1000
D
Mean Length (bp)
2000
1500
1000
*
*
TDP1 siRNA
- + - +
CTG
CCTG
E
DMSO
CPT
Amikacin
Lawsone
SPI-112
BP
Expand
2000
1000
600
Normal
F
Mean Length (bp)
2500
000
1500
***
**
ns
DMSO
CPT
Amikacin
Lawsone
SPI-112

Figure EV5. TDP1/gkt inhibition induce CTG/CCTG repeats contraction in other repeat expansion models.

(**A**, **B**) Representative capillary electropherograms across different genotypes at 60-days-old. Tissue from three animals. (**B**) Quantification of mean length of CTG repeats from five biological replicates. Data are mean ± SD. Two-tailed, unpaired *t* test. *$P = 0.\ 0103$, **$P = 0.\ 0054$. (**C**, **D**) Representative gel image of PCR across different genotypes Hek293 cell line following 7 days of treatment from bioanalyzer. (**D**) Quantification of mean length of CTG/CCTG repeats from three biological replicates. Data are mean ± SD. Two-tailed, unpaired *t* test. *$P = 0.0324$ $(CTG)_{400}$ cell line, *$P = 0.0341$ $(CCTG)_{400}$ cell line. (**E**, **F**) Representative gel image of DM1 patient-derived lymphocytes following 15 days treatment of different compounds from bioanalyzer. (**F**) Quantification of (**E**) from three biological replicates. Data are mean ± SD. One-way ANOVA. **$P = 0.0082$ DMSO versus Lawsone, ***$P = 0.001$ DMSO versus SPI-112.

© The Author(s)
